# Sports Dietitians Australia and Ultra Sports Science Foundation Joint Position Statement: A Practitioner Guide to the Prevention and Management of Exercise-Associated Gastrointestinal Perturbations and Symptoms

**DOI:** 10.1007/s40279-025-02186-6

**Published:** 2025-04-07

**Authors:** Ricardo J. S. Costa, Stephanie K. Gaskell, Kayla Henningsen, Nikki A. Jeacocke, Isabel G. Martinez, Alice Mika, Volker Scheer, Rachel Scrivin, Rhiannon M. J. Snipe, Alice M. Wallett, Pascale Young

**Affiliations:** 1 Department of Nutrition Dietetics and Food, Monash University, Level 1, 264 Ferntree Gully Road, Notting Hill, VIC 3168 Australia; 2 Australian Institute of Sport, Bruce, Canberra, Australia; 3 Ultra Sports Science Foundation, Pierre-Benite, France; 4 University of the Sunshine Coast, Sippy Downs, QLD Australia; 5 Toi Ohomai Institute of Technology, Tauranga, New Zealand; 6 School of Exercise and Nutrition Sciences, Deakin University, Burwood, VIC Australia

## Abstract

It is now well-established that exercise can disturb various aspects of gastrointestinal integrity and function. The pathophysiology of these perturbations, termed “exercise-induced gastrointestinal syndrome (EIGS),” can lead to exercise-associated gastrointestinal symptom (Ex-GIS) inconveniences. EIGS outcomes can impact physical performance and may lead to clinical manifestation warranting medical intervention, as well as systemic responses leading to fatality. Athlete support practitioners seek prevention and management strategies for EIGS and Ex-GIS. This current position statement aimed to critically appraise the role of EIGS and Ex-GIS prevention and management strategies to inform effective evidence-based practice and establish translational application. Intervention strategies with mostly consistent beneficial outcomes include macronutrient (i.e., carbohydrate and protein) intake and euhydration before and during exercise, dietary manipulation of fermentable oligo-, di-, and mono-saccharides and polyols (FODMAP), and gut training or feeding tolerance adjustments for the specific management of Ex-GIS from gastrointestinal functional issues. Strategies that may provide benefit and/or promising outcomes, but warrant further explorations include heat mitigating strategies and certain nutritional supplementation (i.e., prebiotics and phenols). Interventions that have reported negative outcomes included low-carbohydrate high-fat diets, probiotic supplementation, pharmaceutical administration, and feeding intolerances. Owing to individual variability in EIGS and Ex-GIS outcomes, athletes suffering from EIGS and/or support practitioners that guide athletes through managing EIGS, are encouraged to undertake gastrointestinal assessment during exercise to identify underlying causal and exacerbation factor/s, and adopt evidence-based strategies that provide individualized beneficial outcomes. In addition, abstaining from prevention and management strategies that present unclear and/or adverse outcomes is recommended.

**Supplementary Information:**

The online version contains supplementary material available at 10.1007/s40279-025-02186-6.

## Key Points

**Table:** 

Clinical and non-clinical manifestations of exercise-induced gastrointestinal syndrome (EIGS) and subsequent debilitating exercise-associated gastrointestinal symptoms (Ex-GIS) are a common feature of exercise adherence and are exacerbated by various extrinsic and intrinsic factors.
Macronutrient intake during exercise, maintenance of euhydration, dietary manipulations (i.e., FODMAP), and gut training have been shown to provide beneficial outcomes in EIGS and/or Ex-GIS management; while heat mitigating strategies and certain nutritional supplementation (i.e., prebiotics and phenols) have shown promising outcomes, but other dietary manipulations and nutritional supplementation appear less favorable.
Individual athlete assessment, to established main causal factors of EIGS and Ex-GIS, aids tailored and effective prevention or management strategies in translational practice.

## Background

It is now well established that taking part in exercise (i.e., hobby, fitness, and/or health) and sports activities (i.e., training and/or competition) can disturb various aspects of gastrointestinal integrity and function, leading to signs and symptoms of gastrointestinal abnormality. Early reports of abdominal pain and nausea in response to exercise stress have been documented in scientific literature for a century [1]. However, there is speculation that gastrointestinal complaints by those undertaking competitive sports activities have existed undocumented since the ancient and modern Olympiad, which takes into account the reported historical perspective of competitive sports across centuries [2] and what is currently known about how exercise stress impacts the gastrointestinal system [3]. Awareness and understanding of “how and why” exercise stress disturbs the gastrointestinal tract, and subsequently promotes gastrointestinal symptoms, only started to develop during the 1980–90s, with landmark exploratory investigations reporting high incidence and severity of gastrointestinal symptoms in endurance-based exercise [4–8]. From a professional practice perspective, gastrointestinal disturbances and associated symptoms in response to exercise are seen as a common outcome in active and athlete populations adhering to training and/or competition schedules, comprising a substantial case load for practitioners (e.g., sports dietitians, sport and exercise nutritionists, and sports medical practitioners) [9, 10]. Indeed, it is evident at a global level that an increasing number of athletes and their support crews (e.g., sport institutes, sport and fitness professionals, coaches, and/or health professionals) are seeking referral for assessment, intervention, and monitoring for such exercise-associated gastrointestinal issues, especially in the endurance and ultra-endurance sports scene [11–14]. In addition, a substantial number of athletes also access local event medical crew support and/or management information, with the aim of seeking prevention or management of their unwanted and potential performance-debilitating exercise-associated gastrointestinal issues [15–17].

Such exercise-induced gastrointestinal disturbances can range from minor to major symptomatic inconvenience that may impact exercise performance; including reduced workload, cessation of exercise, or withdrawal from activity [17–20]. In turn, these symptoms may potentially signal more serious clinical concerns warranting medical attention; which may include, but are not limited to, fecal blood loss and acute colitis, [21–25] gastroparesis with or without ileus, [12, 26–28] sepsis (i.e., endotoxemia and bacteremia) with subsequent systemic inflammatory response and linked to the pathophysiology of heatstroke, [29–33] and/or chronic inflammatory diseases of the gastrointestinal tract in susceptible predisposed individuals [3]. It is therefore not surprising that there has been an exponential growth in exercise gastroenterology research focusing on strategies to prevent or manage (i.e., attenuate or ameliorate the inevitable) the detrimental effects of exercise on the integrity and function of the gastrointestinal tract. Anecdotal evidence from case reports and referral platforms suggest that practitioners seek expertise in the scientific literature for evidence-based effective and efficient prevention and management strategies to support athletes that present with “exercise-associated gastrointestinal syndrome” (EIGS) and “exercise-associated gastrointestinal symptoms” (Ex-GIS). Therefore, this joint Sports Dietitians Australia (SDA) and Ultra Sports Science Foundation (USSF) position statement will focus on critically appraising the role of EIGS and Ex-GIS prevention and management strategies to provide guidance and recommendations for effective evidence-based practice and establish translational application. A scoping review style approach (i.e., PubMed, SPORTSdiscus, and Ovid Medline) was used to obtain and screen for relevant research studies (i.e., original investigation, field research, and case study, in healthy athlete or active populations) in the respective EIGS and/or Ex-GIS prevention or management strategy assigned to academics, researchers, and/or practitioners with established track records (i.e., research and/or professional practice) in the topic area. Only research papers that provided some level of research methodological competency and/or contributed to the consensus statement discussions were included. Gathered information was used by the author group to establish efficacy in prevention and management of disturbances to gastrointestinal integrity and function, systemic responses, and/or symptoms, with provisions towards a “grade of evidence” established by standardized assessment procedure and group consensus (Sect. 2).

### Exercise-Induced Gastrointestinal Syndrome (EIGS)

**Fig. 1:**
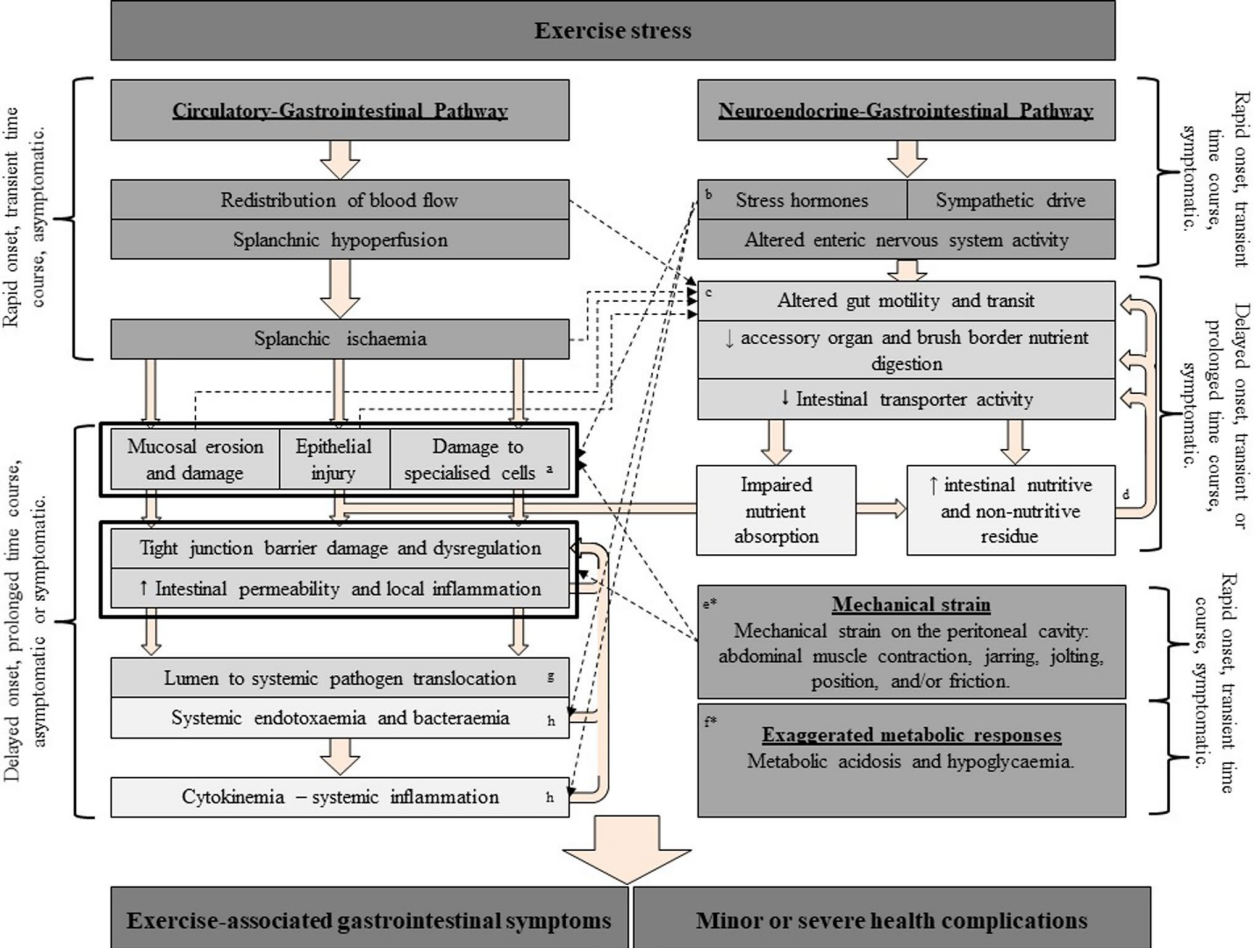
Updated schematic description of exercise-induced gastrointestinal syndrome (EIGS) and links to exercise-associated gastrointestinal symptoms (Ex-GIS). Text boxes: Darkest grey, instigation and final outcome; dark grey, primary causal mechanisms; medium grey, secondary outcomes to the causal mechanisms; light grey, subsequent follow-on outcomes in response to the secondary outcomes. Beige arrows indicate EIGS pathway flow and direction, and black arrows indicate intra-EIGS linkage. ^a^Specialized antimicrobial protein-secreting (i.e., Paneth cells) and mucus-producing (goblet cells) cells aid in preventing intestinal-originating pathogenic microorganisms entering systemic circulation. ^b^Increase in neuroendocrine activation and suppressed submucosal and myenteric plexuses may result in epithelial cell loss and subsequent perturbed epithelial tight junctions [49, 54]. ^c^Splanchnic hypoperfusion and subsequent intestinal ischemia and injury (including mucosal erosion) may result in direct (e.g., enteric nervous system and/or enteroendocrine cell) or indirect (e.g., braking mechanisms) alterations to gastrointestinal motility [27–29, 37, 55]. ^d^Gastrointestinal brake mechanisms: nutritive and non-nutritive residue along the small intestine, including the terminal ileum, results in neural and enteroendocrine negative feedback to gastric activity [44, 55–58]. ^e^Aggressive acute or low-grade, prolonged mechanical strain is proposed to contribute to disturbances to epithelial integrity (i.e., epithelial cell injury and tight junction dysregulation) and subsequent “knock-on” effects for gastrointestinal functional responses [50]. ^f^Metabolic acidosis associated with high intensity exercise and hypoglycemia associated with prolonged endurance exercise may prompt Ex-GIS [51–53]. ^g^Lumen originating to circulatory translocation of pathogenic agents may include, but is not limited to: whole bacteria, bacterial endotoxins (e.g., lipopolysaccharide, lipid A, flagella, and/or peptidoglycan), exocrine originated digestive enzymes, and/or food protein allergens [38]. ^h^Bacteria and bacterial endotoxin MAMPs and stress induced DAMPs are proposed to contribute toward the magnitude of systemic immune responses (e.g., systemic inflammatory profile) [59]. *Primary role in EIGS pathophysiology still warrants substantial exploration and investigation. EIGS, exercise-induced gastrointestinal syndrome; GIS, gastrointestinal symptoms; MAMPs, microorganism molecular patterns; DAMPs, danger-associated molecular patterns. Adapted with permission from “Systematic review: exercise-induced gastrointestinal syndrome- implication for health and disease,” by R.J.S. Costa, R.M.J. Snipe, C. Kitic, and P. Gibson, 2017, Alimentary Pharmacology and Therapeutics, 46(3), pp. 246–265. Copyright 2017 by John Wiley & Sons Ltd. [3]

The etiology and pathophysiology of EIGS is presented in Fig. 1 [3, 34]. EIGS comprises two primary pathophysiological pathways. The circulatory-gastrointestinal pathway describes the splanchnic hypoperfusion and gastrointestinal ischemia that occurs due to a redistribution of blood flow to skeletal muscle and peripheral circulation, addressing the metabolic and thermoregulatory demands of the exercise [35, 36]. Such typical physiological alterations in response to exercise may result in intestinal epithelial injury and hyperpermeability, as well as local and/or systemic inflammatory effects in response to translocated luminal originating pathogens, including but not limited to whole bacteria and/or bacterial endotoxins [33, 37–40]. The neuroendocrine-gastrointestinal pathway describes the stress response contribution to gastrointestinal integrity and functional disturbances, potentially via increases in sympathetic activation and stress hormone responses [26–29]. These neuroendocrine stress responses are synonymous with impaired gastrointestinal motility, transit, digestive function, and nutrient absorption [26–28, 41–46]. They are likely associated with negative impacts on the myenteric and/or submucosal plexuses of the enteric nervous system, and/or the independent intestinal smooth muscle activation of interstitial cells of Cajal (i.e., pace-maker cells of the gastrointestinal tract that are coupled with gastrointestinal smooth muscle and epithelial tissue) [47–49]. Two additional pathways have been proposed, but still warrant substantial investigation for clarity in pathophysiological contribution. These include the mechanical strain of exercise (i.e., jarring, jolting, macro- and micro-vibrations, and/or tissue friction) on the splanchnic area and exaggerated metabolic responses to exercise (i.e., increased pH). First, biomechanical abnormalities aligned with mechanical strain on the splanchnic area may impact the circulatory-gastrointestinal and neuroendocrine-gastrointestinal pathways and potentially lead to hypersensitivity of epithelial, connective, and/or surrounding tissues of the gastrointestinal tract [7, 50]. Second, metabolic acidosis associated with high intensity exercise and hypoglycemia associated with prolonged endurance exercise have been identified as culprits in exercise-associated nausea [51–53].

### Exercise-Associated Gastrointestinal Symptoms (Ex-GIS)

One of the predominant outcomes of EIGS, which has been established to affect exercise performance and may provide an indication of something more clinically sinister, is Ex-GIS. Several reviews have reported on the incidence and severity of Ex-GIS predominantly in observational field studies, specifically in endurance and ultra-endurance activities that focus on running and/or cycling modalities in both elite and recreational populations [17, 20, 60–62]. Reported studies applied baseline and/or retrospective post-race gastrointestinal symptoms (GIS) subjective assessment tools of different origins and applications (e.g., established or in-house fabrications, Likert-type or visual analog scale). Considering the difference in methodological approach, it is not surprising that a large variation in symptom incidence and severity is evident. These range from negligible Ex-GIS incidence in a recreational endurance running event, [61] 4% after marathon competition, [20] and ≥ 93% in response to ultra-endurance event participation, [19, 63] with severity ranging from minor inconvenience to severe that warranted event withdrawal. These and other reports provide some indication that longer-duration exercise, especially when conducted in hot ambient conditions, tends to promote higher incidence rates of Ex-GIS [17]. Moreover, longer duration exercise bouts that require exogenous feeding tend to induce more Ex-GIS incidence despite a lower exercise intensity, which has been linked to individual variation in feeding tolerance [20, 41–43, 64–66]. To improve consistency in Ex-GIS interpretation within exercise gastroenterology research, the use of a standardized exercise-specific validated and reliable GIS assessment tool has been recommended [67, 68].

The type of Ex-GIS reported, in accordance with the ROME criteria for symptom type (i.e., currently ROME IV criteria), [69, 70] can be classified as either gastro-esophageal (i.e., upper-GIS): belching, heartburn (gastroesophageal reflux), upper-abdominal bloating or pain, urge to regurgitate, and/or regurgitation; intestinal (i.e., lower-GIS): flatulence, lower abdominal bloating or pain, urge to defecate, abnormal defecation, and/or fecal blood loss; and/or other symptoms not otherwise specified (i.e., nausea and acute transient abdominal pain—stitch) [68]. Despite mainstream dogma proposing that lower-GIS symptoms (e.g., diarrhea) are more commonly linked to exercise, the scientific literature does not support this notion, with upper-GIS consistently being more commonly reported during and/or immediately after exercise, both in laboratory controlled and field-based experimental models [17, 20, 42, 71–73]. In addition, nausea appears to be a frequently reported symptom in ultra-endurance activities, [19, 20, 63, 74] which is rarely reported in lesser exercise loads or experimental models [20, 44, 61, 65, 72]. Taken together, the available research suggests that the magnitude (i.e., exercise duration, intensity, and environmental factors) of exertional stress and requirement for feeding during the exercise plays a key role in Ex-GIS incidence and severity. The incidence type, severity, time of onset, and when symptoms subside may provide some indication of relationships between Ex-GIS and EIGS pathophysiology (Fig. 1).

### EIGS and Ex-GIS Exacerbation Factors

Several extrinsic and intrinsic factors have now been identified and/or confirmed to exacerbate EIGS and Ex-GIS, ranging from minimal, to modest, to more extensive impact; which has been summarized in Table 1. Consensus impact was assigned on the basis of: (1) the number of research studies (i.e., cross-sectional analysis, exploratory, or intervention laboratory-controlled trials) investigating the impact of the exacerbation factor or factors on gastrointestinal and/or systemic perturbations to exercise; (2) the quality of experimental procedures and control of confounding factors in accordance with exercise gastroenterology research best-practice recommendations checklist [67]; (3) the magnitude of response (i.e., none, to modest, to clinically relevant minimal detectable change) of any identified gastrointestinal and/or systemic perturbations to exercise [33]. Assigned magnitude of impact on EIGS and/or Ex-GIS may include: unknown (?), minimal ( +), moderate (+ +), and extensive (+ + +). These factors and their potential magnitude of impact need to be considered when assessing the potential effectiveness of proposed prevention or management strategies for EIGS and Ex-GIS, and using such strategies for individual athlete therapeutic intervention. This is important, as these constitute potential confounding factors for exercise-associated disturbances to gastrointestinal integrity and function and systemic immune responses [12, 75].

**Table 1:** Summarized overview of extrinsic and intrinsic exacerbation factors of exercise-induced gastrointestinal syndrome (EIGS) and exercise-associated gastrointestinal symptoms (Ex-GIS). Critical explanation includes impact on markers of gastrointestinal integrity and function, systemic responses, symptomology, and degree of exposure required to instigate perturbation to minimal detectable change [33]

Factors	Impact	Description	References
**Extrinsic factors**			
Exercise duration	+++	- Increasing exercise duration, irrespective of intensity, results in increased EIGS integrity and systemic response biomarkers and Ex-GIS, likely attributed to splanchnic hypoperfusion and subsequent gastrointestinal ischemia - Exercise stress per se impairs gastrointestinal function without any duration impact (i.e., 1–3 h of exercise comparison), likely attributed to sympathetic drive and stress hormone responses, with or without splanchnic hypoperfusion effects - Considerable perturbations occur with ≥ 2 h exercise duration compared with < 2 h, but appears to plateau ≥ 2 h thereafter - Substantial Ex-GIS, with episodes of medical management, in prolonged endurance and ultra-endurance events. However, the full impact of ultra-endurance exercise activities on EIGS integrity/systemic and functional outcomes is still unknown and warrants further exploration	[17, 26, 28, 71, 76]
Exercise intensity	++	- Increasing exercise intensity (e.g., continuous ≥ 70% *V*O_2max_ or HIIT or resistance exercise of ≤ 2 h) results in modest increases in EIGS integrity and systemic response biomarkers, functional responses, and Ex-GIS	[33, 40, 77–80]
Exercise modality	+	- Field exploratory studies suggest running-based activities prompt greater gastrointestinal disturbance and Ex-GIS compared with swimming and cycling (i.e., with or without inclusion in triathlon events) modalities, proposed to be linked with the addition of mechanical strain on the splanchnic area - Controlled laboratory studies have not provided substantial evidence to suggest exercise modality differences (i.e., running vs. cycling) for markers of EIGS and Ex-GIS - Impact of other exercise activities with respective body positioning and mechanical strain (e.g., swimming, canoeing and kayaking, motor and/or winter sports) on EIGS markers and Ex-GIS is warranting exploration	[7, 20, 81]
Environmental conditions	+++	- Exercising in hot ambient conditions (e.g., ~ 35 °C), irrespective of relative humidity, results in increases in EIGS integrity and systemic response biomarkers, and Ex-GIS. Correlation and regression analysis have confirmed a positive association and prediction between maximal and Δ core body temperature with pathophysiological markers of EIGS. Core body temperature values of ≥ 39.5ºC or Δ ≥ 2.5ºC indicative of clinical significance^a^, but values of ≥ 39.0ºC or Δ ≥ 2.0ºC are reported above minimal detectable change and of clinical relevance - Exercise stress per se impairs gastrointestinal function without any substantial additional heat stress impact - Other thermoregulatory modifiers that may influence maximal (e.g., ≥ 39 °C) or magnitude of change (≥ 2 °C) in core body temperature (e.g., clothing, wind speed, water temperature, hydration status, heat acclimation/acclimatization and/or individual thermoregulatory responses (i.e., heat tolerance) may result in increases in EIGS integrity and systemic response biomarkers, and Ex-GIS	[27, 33, 72, 82, 83]
Topographical elevation*	+	- Some evidence suggested that altitude (e.g., > 2500 m ASL) at which exercise is performed may increase disturbances to gastrointestinal integrity and function, and subsequently exacerbate Ex-GIS. However, further and repeatable exploration of the impact of altitude on EIGS integrity and functional markers is warranted	[11, 84–86]
Time of day exercise is performed (circadian variation)	++	- Nocturnal exercise prompts greater disturbance to gastrointestinal function responses and subsequent Ex-GIS compared with diurnal exercise - No effect of nocturnal versus diurnal exercise on EIGS integrity and systemic biomarkers	[26]
External pharmaceutical administration	+++	- Nonsteroidal anti-inflammatory drugs (NSAIDs) are considered gastrointestinal irritants, impacting stomach gastric secretions, bicarbonate release in the duodenum, and/or erosion of the mucosal lining along the gastrointestinal tract - The administration of NSAIDs prior to exercise can markedly increase gastrointestinal integrity perturbations, impair functional responses, and/or exacerbate Ex-GIS	[87–92]
**Intrinsic factors**			
Biological sex*	+	- Differences in gastrointestinal integrity and function, systemic responses, and Ex-GIS between male and female participants in exercise gastroenterology research have been observed. Outcomes have not been consistent, with both biological sexes reporting varied markers of EIGS and Ex-GIS dependent on the experimental model. Any differences have also been low to modest in nature - It is proposed that female athletes present greater EIGS and Ex-GIS than male athletes owing to the menstrual cycle. However, clear and consistent evidence with substantial magnitude to support this plausibility is still warranted	[42, 93–95]
Age*	+	- Some speculation has suggested that the developing gastrointestinal tract and immune system of youth athletes (≤ 18 years) may result in greater EIGS marker responses and Ex-GIS compared with well-trained and exercise-experienced adults. However, current evidence suggests any age-related differences are modest in nature - Despite active older adults (≥ 40 years) presenting differences in gut bacterial composition (i.e., fecal profile) compared with younger active adults (≤ 30 years), only modest differences have been reported in EIGS integrity and/or systemic biomarkers, and Ex-GIS, between these two age populations - The full extent of exertional or exertional-heat stress on EIGS integrity/systemic and functional outcomes and Ex-GIS in developing youth athletes and older masters athletes is still unknown and warrants exploration	[93, 96–98]
Fitness status	+	- Higher-trained individuals, and individuals with the ability to cope with greater exertional or exertional-heat stress loads (i.e., duration, intensity, and heat), are at lower risk of EIGS compared with lower-trained individuals at the same absolute load. However, the ability to cope inevitably exposes higher-trained individuals to these higher exertional or exertional-heat stress loads and subsequently poses greater relative EIGS and Ex-GIS risk - Higher-trained individuals have a greater ability to cope with feeding and/or drinking during exercise (i.e., greater feeding tolerance) and the same absolute intake (i.e., volume, concentration, type, and/or texture); therefore, they present lower risk of Ex-GIS. However, the ability to better cope with feeding during exercise inevitably pushes higher trained individuals to attempt greater feeding volumes, and subsequently poses greater relative Ex-GIS risk	[15, 16, 20, 65, 66, 99]
Gastrointestinal and/or circulating microbial and/or short chain fatty acid composition*	+	- The bacterial composition of the gastrointestinal tract (i.e., commensal versus pathogenic bacteria), and subsequent levels of short chain fatty acids (SCFAs; e.g., acetate, butyrate, and/or propionate) in the lumen and/or circulation may impact the magnitude to which gastrointestinal integrity and systemic responses are perturbed, and Ex-GIS are instigated, in response to exertional or exertional-heat stress	[29, 33, 93]
Individual characteristics*	+++	- The feeding tolerance to food and/or fluid intake volume (e.g., mL/h), concentration (e.g., 6% versus > 10% carbohydrate solution), type (e.g., macronutrient profile and/or singular versus multiple transportable carbohydrates), and texture (e.g., solid versus liquid) may increase the risk of EIGS and Ex-GIS (Supplementary file 1) - Individuals presenting gastrointestinal structural or function diseases or disorders may be predisposed to greater incidence and severity of EIGS and Ex-GIS - Individuals with recurrent Ex-GIS with or without etiological and/or pathophysiological confirmation and/or clinical diagnosis are likely to present more incidence and severity of EIGS and Ex-GIS	[12, 41–43, 64, 66, 100, 101]
Psychological state*	?	- It has been proposed that a pre-exercise state of anxiety is linked to Ex-GIS on the basis of field exploratory observations and/or survey/questionnaire research designs. Such experimental approaches lack the rigors of laboratory and confounder-control experimental protocols, as previously described in pre-exercise mental prompts and physiological response to exercise [102]. To date, only one study has explored the role of pre-exercise anxiety state using a sport anxiety questionnaire of EIGS integrity, systemic, and/or functional markers, and subsequent Ex-GIS, using controlled experimental procedures [106]. Somatic trait anxiety scores were not correlated with Ex-GIS in both exercise trial occasions (i.e., pre- and post- intervention)	[103–105]

Impact on magnitude of response in EIGS markers and Ex-GIS include: unknown: ?; minimal: + ; modest:++; extensive:+++; and further research warranted since current literature provides only extrapolation of study outcomes and speculation: *. Comments and impact outcomes are a general representation of reference literature. The high variability in intra- and inter-individual responses in EIGS markers and Ex-GIS in response to exertional and exertional-heat stress models, and feeding tolerance (i.e., volume, concentration, type, and texture) intake during exercise, is acknowledged. ^a^Clinical significance: above established minimal detectable change, and/or aligned with impacting exercise output, severe Ex-GIS, and/or warranting medical management. The role of dietary intake and hydration status as potential intrinsic exacerbation factors is covered in Sects. 5 and 7, respectively

ASL, above sea level; EIGS, exercise-induced gastrointestinal syndrome; Ex-GIS, exercise-associated gastrointestinal symptoms; h, hours; HIIT, high intensity interval training; NSAIDs, non-steroidal anti-inflammatory drugs; SCFA, short chain fatty acids; *V*O_2max_, maximal oxygen uptake

## Methodological Considerations in Exercise Gastroenterology Research and Translational Practice

In the last decade, there has been exponential growth in EIGS and Ex-GIS prevention and management strategy research in response to potential performance and health implications. It has, however, been highlighted that a large proportion of EIGS and Ex-GIS prevention and management strategy intervention studies contain substantial limitations in experimental methodologies to a degree that may influence data outcomes and interpretations in professional practice [67]. Areas of identified methodological concern include, but are not limited to: the clear selection and screening of participants; the justification of exercise protocols (e.g., intensity, duration, and modality); ambient temperature control and/or application of sufficient heat stress; dietary control and nutritional provision before and during exercise; regulation and monitoring of hydration status; the justification of EIGS measurement variables (e.g., intestinal epithelial tissue integrity and pathogenic translocation biomarkers, systemic inflammatory response biomarkers, and/or gastrointestinal functional responses) and application of a suite of such markers; and the choice of Ex-GIS assessment tool employed. In addition, the consideration for other identified outcome and interpretation impacting factors have been acknowledged, such as power calculations to support sample size; data presentation (i.e., baseline values, absolute and relative change); inclusion of broad heterogeneous populations (e.g., biological sex, age, fitness status, and/or modality participation); and/or inclusion of confirmed erroneous measurement variables and analytical techniques. It has been argued that “such limitations increase the risk of misrepresenting research outcomes, which can have significant translational implications for practitioners, with outcomes ranging from ineffective interventions to the risk of fatality” [67]. In the context of the current review, the experimental design checklist, as presented in Costa et al. (2022), was followed to ascertain the experimental quality and subsequent justification for inclusion in assessing the level of evidence for overall therapeutic strategy efficacy in the prevention and management of EIGS and Ex-GIS. Table 2 provides details of categories and types of disturbance to gastrointestinal integrity and function, and systemic responses, commonly reported in the published literature. In addition, it provides a level of evidence based on best practice recommendations in exercise gastroenterology research. Research that predominantly meets best-practice recommendations in exercise gastroenterology research may be assigned “Grade I” or “Grade II” level of evidence, while research that does not predominantly meet best-practice criteria is limited to “Grade III” evidence.

**Table 2:** Categories of exercise-associated gastrointestinal disturbance (A) and level of evidence for establishing efficacy of exercise-induced gastrointestinal syndrome (EIGS) and exercise-associated gastrointestinal symptoms (Ex-GIS) prevention and management strategies (B)

A. Disturbance category		Disturbance type	
**Gastrointestinal epithelial integrity**		Intestinal epithelial tissue injury, tight-junction protein injury, gastrointestinal permeability, systemic endotoxemia and/or bacteremia	
**Gastrointestinal function**		Gastric emptying, intestinal transit, nutrient absorption, smooth muscle myoelectrical activity, and/or malabsorption of feeding challenge	
**Systemic responses**		Leukocyte trafficking, systemic inflammatory cytokine responses, immune functional response to endotoxemia and/or bacteremia	
**Gastrointestinal signs and symptoms**		Gut comfort, total-GIS symptoms, upper-GIS symptoms, lower-GIS symptoms, nausea, regurgitation or projectile vomiting with or without blood loss, abnormal defecation with or without blood loss	
**Feeding tolerance**		Taste and/or flavor fatigue, interest in food/drink, tolerance in food/drink, appetite, and thirst, with or without GIS impact	

B. Grades of evidence*		Description exemplar	Practical application
Good	**Grade I**	SLR, RCT, and/or original investigation with predominantly repeatable and consistent outcomes**	Yes
Fair	**Grade II**	RCT and/or original investigation**; however, limited to single or minimal studies	Yes
Limited	**Grade III**	RCT and/or original investigation, with limited, conflicting or unclear outcomes, with or without single or minimal studies	Yes—with caution and justification
Not fully supported or refutable	**Grade IV**	Original investigation with or without questionable, conflicting, inconsistent outcomes, and/or unclear in nature (i.e., methodologies and results including data analysis and interpretation) Field observational data, or individual case-study and case-series approaches	Not warranted and justified, or application with caution and justification
No clear evidence	**Grade V**	Not assignable—no evidence to support or refutable conclusions	Not warranted or justified

^*^Adapted from Thomas et al. [107]. **Predominately based on experimental procedure best practice recommendation checklist from Costa et al. [67]. Further discussion within each specific section when experimental protocols do not adhere to proposed best practice guidance. GIS, gastrointestinal symptoms; RCT, randomized control trial; SLR, systematic literature review

## EIGS and Ex-GIS Prevention and Management Strategies

To assess the impact of researched interventions on the prevention and management of the various primary causal mechanisms, pathophysiological pathways, secondary outcomes, performance and/or clinical manifestation, Table 2 provides a general overview of exercise-associated gastrointestinal disturbance categories and outcomes. Table 3 presents a summary of evidence of efficacy. The level of evidence used, after screening for best practice experimental quality, [67] is also described in Table 2 and was adapted from levels of evidence presented in Thomas et al. [107] Areas of coverage include macronutrient intake before and during exercise, dietary interventions, nutritional supplement interventions, hydration status, heat stress mitigating strategies, gut training and tolerance to feeding during exercise (supplementary file 1) in specific relation to Ex-GIS. Other prevention and management strategies (i.e., physical applications and pharmaceutical interventions) and considerations (i.e., recovery nutrition) explored are presented in the supplementary materials (supplementary file 2).

**Table 3:** Summary of the efficacy of proposed exercise-induced gastrointestinal syndrome (EIGS) and exercise-associated gastrointestinal symptoms (Ex-GIS) prevention and management strategies taking into account: (1) experimental procedures following best practice recommendation, and (2) magnitude of impact in reducing EIGS assessment markers and Ex-GIS

Intervention	Integrity	Function	Systemic	Ex-GIS	Grade of evidence	EIGS/Ex-GIS potential prevention and management strategy	Key comments
**Macronutrients (Before and/or during)**							
Carbohydrate	✓	?^a^	✓	N and X^a^	I	Yes/Yes	Ex-GIS: volume and type dependent
Glutamine	N and ?	?	N	N	III	No/No	Unclear due to inconsistent research outcomes and research methodological issues in some studies**
Arginine	N	?	N	X	III	No/Yes	Avoid intake to ↓ Ex-GIS
Citrulline	✓	?	N	?	II	Yes/No	Benefit possibly aligned with nutrient presence
Amino acid combinations	✓	?	✓	N	II	Yes/Yes	Dependent on specific formulations
Whole protein	✓	?	✓	X	II	Yes/Yes	Ex-GIS: volume and tolerance dependent
**Dietary interventions (short- and/or long-term)**							
Gluten-free diet	N	?	N	N	III	No/No	Gluten free diets inadvertently reduce FODMAP load
Low FODMAP diet	X	✓	N	✓	I	No/Yes	↑ EIGS pathophysiology, but ↓ Ex-GIS severity. ↓ malabsorption risk from a functional perspective
High FODMAP diet	✓	X	N	X	I	Yes/No	↓ EIGS pathophysiology, but ↑ Ex-GIS severity. ↑ malabsorption risk from a functional perspective
LCHF	X	?	X	?	II	No/No	Dietary lipid intake and digestion/absorption are synonymous with luminal pathogenic translocation
LEA (short-term)	N	?	N	N	III	No/No	Longer periods of LEA leading to REDs diagnosis may result in EIGS and/or Ex-GIS
Low fiber/residue diet	?	?	?	✓	IV	No/Yes	Only field observational data and case study approach evidence available. No controlled experimental trial data available
**Nutritional supplements**							
**(short- and/or long-term)**							
Antioxidant—Ascorbic acid	✓	?	?	?	V	No/No	Research methodologies not aligned with best practice recommendations**
- Tocopherol	N	?	?	✓	IV	No/No	Research methodologies not aligned with best practice recommendations**
- Capsaicin	?	?	N	X	III	No/No	Supplementation resulted in greater Ex-GIS incidence
Biotics- Prebiotics	✓	N	N	N	II	Yes/No	Also refer to FODMAP research, considering the prebiotic properties of FODMAP
- Probiotics	N	?	N	N	I	No/No	Proposed outcomes from SLR
- Synbiotics	N	?	N	N	I	No/No	Proposed outcomes from SLR
Bovine colostrum	N	?	N	?	II-III	No/No	Unclear due to inconsistent research outcomes and research methodological issues in some studies**
Curcumin	✓	?	✓	?	III	Yes/No	Further research using more methodologically robust studies is required to establish clinical or practice relevance**
Anthocyanins	✓	?	N	?	III	Yes/No	Only modest attenuation observed in intestinal epithelial injury, with no effects on pathogenic translocation observed
Nitrate	N	?	N	N	III	No/No	Further research using more methodologically robust studies are required to establish clinical or practice relevance**
**Hydration strategies**							
Pre-exercise euhydration	✓	✓	?	✓ and X^a^	II	Yes/Yes	Start exercise euhydrated, but without fluid overload that may overburden gastric tolerance
During exercise euhydration	✓	✓	N	N and X^a^	II	Yes/Yes	Maintain euhydration, but without fluid overload that may overburden gastric tolerance
**Heat mitigating strategies**							
Heat acclimation	N	?	N	N	III	Yes/Yes^b^	Positive impact on core body temperature evidence; but further research with longer exposure is warranted to thoroughly assess impact on EIGS
Internal pre- and per-cooling	N	?	N	N	III	Yes/Yes^b^	Positive impact on core body temperature evidence; but further research on pre- and/or per-cooling, with or without external cooling strategies, is warranted to thoroughly assess impact on EIGS
External pre-and per-cooling	?	?	✓	?	III	Yes/Yes^b^	Positive impact on core body temperature evidence; but further research on pre- and/or per-cooling, with or without internal cooling strategies, is warranted to thoroughly assess impact on EIGS
**Gut training**							
Repetitive feeding challenge	N	✓	?	✓	I	No/Yes	Magnitude of effect dependent on repetitive challenge exposure (i.e., quantity, quality, and duration), and individual responsiveness
Noncaloric sweeteners	?	?	?	N	III	No/No	Only one study that used sucralose pre-exercise, and Ex-GIS was a secondary variable of focus
Fluid tolerance training	?	?	?	✓	III	No/Yes	Only one study that used sweat rate matched fluid challenge versus ad libitum (within comfort), and Ex-GIS was a secondary variable of focus
**Other strategies**							
Pharmaceutical administration							
- Antiemetics	?	?	?	N	V	No/No	One serotonin 5-HT3 receptor antagonist field-based study, with no confounder control, and only assessed nausea and vomiting
- Antacids	✓	?	?	✓	IV	No/No	H_2_ receptor antagonists and proton-pump inhibitor field-based studies with no confounder control, and only assessed nausea, vomiting, and/or fecal blood presence
Physical maneuvers - Abdominal muscle contraction, - Modified breathing, - Abdominal belt tightening	?	?	?	✓	IV	No/No	Only one study that focused on reducing Ex-GIS intensity with proposed physical maneuver
Compression socks	✓	?	?	?	IV	No/No	Only one study that applied a parallel intervention experimental design in a field race, with no adequate confounder control
Feeding tolerance***							
Intake volume	–	–	–	X	I	–/Yes	↑ volume ↑ the risk of Ex-GIS
Carbohydrate concentration	–	–	–	X	I	–/Yes	↑ carbohydrate intake concentration ↑ the risk of Ex-GIS
Carbohydrate dose and frequency	–	–	–	X	III	–/Yes	↑ carbohydrate intake dose and ↓ frequency increases the risk of Ex-GIS
Intake texture form	–	–	–	X	I	–/Yes	Ex-GIS risk ↑ from liquid to solid form
Carbohydrate type							
- Fructose	–	–	–	X	II	–/Yes	↑ fructose intake ↑ risk of Ex-GIS
- Glucose molecular weight	–	–	–	N	II	–/No	↑ in molecular weight of carbohydrate type does not generally and substantially ↑ the risk of Ex-GIS
- Isomaltulose	–	–	–	X	IV	–/No	↑ Ex-GIS with singular isomaltulose use
- Multiple transportable carbohydrates	–	–	–	N	III	–/No	Contradictory findings, methodological issues, and questionable result interpretation in research that has explored Ex-GIS on the impact of multiple transportable carbohydrate blends versus singular carbohydrate on Ex-GIS. Onset of Ex-GIS appears to depend on individual tolerance and linked to malabsorption (i.e., namely fructose)
- Modified starch	–	–	–	X	III	–/Yes	Use of modified starch carbohydrate ↑ risk of Ex-GIS
- Hydrogel	–	–	–	N	I	–/No	Use of hydrogel carbohydrate products pre- and/or during exercise does not lower the incidence or severity of Ex-GIS
Caffeine inclusion	–	–	–	N	I	–/No	Caffeine, with or without carbohydrate pre- and/or during exercise, in modest doses does not exacerbate Ex-GIS compared with water and carbohydrate alone
Coconut water	–	–	–	N	III	–/No	Coconut water during exercise does not exacerbate Ex-GIS compared with water
Dairy (containing lactose)	–	–	–	N	III	–/No	Dairy intake pre-exercise does not exacerbate Ex-GIS compared with a carbohydrate control comparator

✓: beneficial effect reported in research; X: detrimental effect reported in research; N: no effect reported in research; ?: unclear (due to conflicting outcomes, no study and/or warrants further exploration); NA: not applicable; ↑: increase; ↓: decrease (note, symbol does not provide indication of the magnitude of beneficial to detrimental effect). EIGS: exercise-induced gastrointestinal syndrome; Ex-GIS: exercise-associated gastrointestinal symptoms; FODMAP: fermentable oligo-, di-, and mono-saccharides and polyols; LCHF: low-carbohydrate high-fat diet; LEA: low energy availability. ^a^Volume- and type-dependent, ^b^targeted at lowing peak and relative change in core body temperature [83] **based on experimental procedure best-practice recommendation checklist from Costa et al. [67] and ***based on experimental procedures best-practice recommendations for confounding factors and assessment tool quality for Ex-GIS

## Macronutrients and Derivatives

A variety of macronutrients or their derivatives, which are consumed for a short- (< 24 h) or long-term (≥ 24 h) period before and/or during an exertional or exertional-heat stress model, have been studied for their efficacy in the prevention and management of EIGS. These include carbohydrate, protein, certain singular amino acids (i.e., glutamine, cysteine, arginine, and/or L-citrulline), and amino acid mixture formulations. Such nutritional intervention approaches appear to target splanchnic perfusion dynamics via villi microvascular regulation, epithelial cell metabolism and/or stability, and/or epithelial tissue tight-junction stability [37, 108–112].

### Carbohydrate

Carbohydrate ingestion during prolonged exercise is common practice, and its effects on EIGS and Ex-GIS are therefore of interest. Carbohydrate ingestion immediately before or during exercise may attenuate disturbances to gastrointestinal integrity associated with EIGS through postprandial hyperemia [108, 112]. Carbohydrate ingestion during exercise also aids the maintenance of blood glucose that attenuates stress hormone responses and potential rises in systemic inflammatory cytokines; [73] thus, it may play a role in mitigating EIGS. On the contrary, excessive carbohydrate ingestion and the type of carbohydrate ingested during exercise may adversely affect the function of the gastrointestinal tract (e.g., delayed gastric emptying and/or malabsorption) and lead to Ex-GIS, subsequently impacting exercise performance (Sect. 9 and supplementary file 1).

The majority of research to date suggests that carbohydrate ingestion before and/or during exercise can minimize or prevent perturbations to gastrointestinal integrity [73, 114–116]. For example, carbohydrate doses of 15.0–22.5 g every 15–30 min (20–90 g/h) during cycling or running exercise of 1–2 h duration, with or without heat exposure (32–35 °C and 27–70% relative humidity (RH)), has attenuated the rise in plasma intestinal fatty acid binding protein (I-FABP; a surrogate biomarker indicative of intestinal epithelial cell injury) concentration and small intestine permeability (i.e., via dual-sugars test for lactulose to rhamnose (L:R) ratio) compared with ingestion of water alone [73, 114–117]. Two studies did not observe a reduction in I-FABP with carbohydrate intake of 27 g/h during 60 min running (70% *V̇*O_2max_, 30 °C) and 108 g/h during 3 h cycling [118, 119]. However, these studies failed to show any substantial increase in I-FABP compared with the control, suggesting that the lack of effect may be due to insufficient thermophysiological impact rather than carbohydrate ingestion [67]. Moreover, another study that showed a reduction in I-FABP with carbohydrate intake compared with water failed to observe differences in splanchnic perfusion (i.e., gap between gastric and arterial pCO_2_) by gastric tonometry [115], potentially owing to the lack of sensitivity of this method to detect difference in perfusion at the level of the intestinal villi. In the aforementioned studies, there has been no difference observed in markers of endotoxemia (i.e., lipopolysaccharide (LPS) and lipopolysaccharide binding protein (LBP)). However, Snipe et al. [73] observed an increased endogenous endotoxin core antibody (EndoCAb IgM) concentration with carbohydrate ingestion and depressed concentrations with water. This suggests potential activation of EndoCAb, which is associated with systemic appearance of pathogenic agents, was overused in the water trial, but not on the carbohydrate trial, irrespective of the post-exercise LPS concentration (i.e., via limulus amebocyte lysate gram-negative bacterial endotoxin determination). Only one study has explored systemic inflammatory responses in conjunction with other EIGS markers with carbohydrate intake and found attenuation of interleukin (IL)-6 compared with water intake, but no differences in the exertional-heat stress induced increases in proinflammatory (i.e., tumor necrosis factor alpha (TNFα) and IL-1β) or anti-inflammatory (i.e., IL-10 and IL-1ra) cytokines between the carbohydrate and water control trials [73]. Together, the current research shows good evidence that ingestion of moderate (20–60 g/h) and/or high (> 60 g/h) amounts of carbohydrates during exercise is an effective strategy to attenuate disturbances to gastrointestinal integrity and systemic responses. However, carbohydrate ingestion tolerance will dictate intake impact on gastrointestinal functional responses and Ex-GIS incidence and severity (Sect. 9 and supplementary file 1).

**Grade of Evidence: I**

### Glutamine

The ingestion of protein (i.e., containing a variety of amino acids) and/or specific amino acids (and derivatives) may play a role in preventing EIGS through the provision of energy substrate to intestinal epithelial cells, regulation of intestinal barrier structure and function, and intestinal immune function [120]. The most well-researched singular amino acid to date for EIGS prevention or management is glutamine, on the basis of its provision as a primary fuel source for epithelial enterocytes [121]. Several studies have demonstrated that acute (0.25–0.9 g/kg fat-free mass (FFM) consumed 2 h before exercise) and prolonged (0.9 g/kgFFM/day for 7 days and 3 g L-glutamine with 0.69 g L-cysteine for 5 days) glutamine supplementation attenuated the modest rise in exercise-associated small intestine permeability in response to 60 min running at 65–75% *V̇*O _2max_ in 25–30 °C and 12–60% RH [122–125]. However, the studies failed to show any beneficial effects on luminal to systemic pathogenic translocation (e.g., bacterial endotoxin) and systemic inflammatory cytokine responses (e.g., TNFα). A dose response study demonstrated small reductions in plasma I-FABP concentration with higher (0.5 and 0.9 g/kgFFM), but not lower (0.25 g/kgFFM), acute glutamine supplementation compared with a non-nutritive placebo [122]. These findings contrast with other studies that have observed small, but significant reductions, in plasma I-FABP concentrations pre-exercise on the second day of exercise in the heat with low glutamine doses provided 1 h before exercise (0.15 g/kgBM) and post-exercise with prolonged (3 g/day L-glutamine with 0.69 g/day L-cysteine for 5 days) supplementation [123, 126]. It should be noted that values present in all studies for gastrointestinal integrity disturbance through the experimental procedure timeline were minor and of little clinical relevance [33, 67]. In contrast, several recent studies have shown no benefit to EIGS prevention and management with glutamine ingested before exercise. For example, 0.9 g/kgFFM L-glutamine ingested 60 min before a 20 km cycling time trial (~ 33 min) in 35 °C and 51% RH did not attenuate the post-exercise modest rise in plasma I-FABP, IL-6, or TNFα concentrations, and responses were not different to the non-nutritive placebo [127]. Similarly, ingestion of 0.3 g/kgFFM L-glutamine 60 min before 80 min treadmill walking at 6 km/h with 6% gradient in 35 °C and 30% RH and run to exhaustion (~ 22 min) at lactate threshold speed in 40 °C and 40% RH did not substantially attenuate disturbances to gastrointestinal integrity [128, 129]. Methodological limitations have been raised in these studies, questioning the results validity into real-world practice [67]. The majority of studies have not reported Ex-GIS with L-glutamine ingestion [122, 127–129]. However, a dose–response study showed increased Ex-GIS with glutamine ingestion at 0.9 compared with 0.6 and 0.3 g/kgFFM, which were more pronounced during the 2 h following ingestion [130]. Overall, there are conflicting findings on the effect of glutamine on EIGS, with any beneficial effects reported linked to magnitude of response being small and of no clinical relevance.

**Grade of Evidence: III**

### Arginine and Citrulline

L-arginine and L-citrulline (i.e., L-arginine precursor) may enhance nitric oxide production at the intestinal villi microvascular compartment, with a proposed subsequent enhancement in splanchnic perfusion that would support the maintenance of gastrointestinal epithelial integrity during exercise [131, 132]. Laboratory-controlled research with L-arginine and EIGS outcomes is lacking. However, ingestion of 30 g/day L-arginine for 14-days before a marathon did not have any effect on small intestine permeability, fecal occult bleeding, or Ex-GIS compared with equivalent supplementation with glycine [132]. Although, it should be noted that small intestine permeability, assessed by urinary dual-sugar (i.e., lactulose and mannitol) probe was not significantly elevated post-marathon compared with pre-race. Whilst this study did not observe differences in Ex-GIS, a review of arginine side effects suggests that GIS, particularly diarrhea, may occur with L-arginine intake with single doses of > 9 g or daily doses of > 30 g/day [133]. Indeed, a small increase in Ex-GIS (i.e., nausea and fullness) with the addition of L-arginine to a carbohydrate electrolyte solution has been observed during prolonged (2.5 h) submaximal (50% peak power) cycling exercise [134].

L-citrulline may enhance L-arginine-derived nitric oxide production, as the ingestion of 10 g of L-citrulline 30 min before 60 min cycling at 70% maximum workload has been shown to modestly attenuate splanchnic hypoperfusion and attenuate the rise in plasma I-FABP concentration, but with no effect on gastric or small intestine permeability [131]. It is also noteworthy to report the attenuated hypoperfusion and epithelial injury on L-citrulline supplement intervention increased to similar values as placebo immediately post-exercise. Therefore, it is not clear whether the acute attenuation during exercise was due to mechanistic effects of the L-citrulline or simply just having nutrients (e.g., amino acid derivative) along the gastrointestinal tract, as per the mechanistic alignment with carbohydrate feeding before and during exercise (Sect. 4.1).

**Grade of Evidence: II to III**

### Amino Acid Combinations

In response to the lack of clarity on the impact of singular amino acids or derivatives on biomarkers representative of EIGS, recent studies have investigated the impact of amino acid combinations on EIGS biomarkers. First, collagen peptides (10 g/day for 7 days and 45 min pre-exercise) did not influence a cluster of intestinal epithelial integrity biomarkers and inflammatory cytokines compared with a placebo in response to 70 min running at 70–90% *V̇*O_2max_ in 22 °C [135]. The application of a multiple amino acid (i.e., 4.5–6.4 g/L: valine, aspartic acid, serine, threonine, and tyrosine, with or without isoleucine) beverage intervention for 7 days, immediately pre-exercise, and during 2 h running at 60% *V̇*O _2max_ in ~ 35 °C attenuated intestinal epithelial cell injury, perturbations to bacterial and endotoxin profiles, and systemic inflammatory responses, but not circulatory bacterial DNA presence and/or alterations to systemic bacterial profile compared with the water control trial [136, 137]. Furthermore, the amino acid content within the beverage provisions did not substantially exacerbate Ex-GIS incidence and severity in response to the exertional-heat stress compared with water. It was reported that these beneficial effects may be due to the 7-day supplementation period promoting amino acid delivery before the stress exposure, subsequently improving the robustness and stability of the intestinal enterocyte cell membrane and/or tight-junction structure and function [108, 111, 112, 120, 138]. Additionally, intake during exercise may have supported villi microvascular perfusion via the nitric oxide pathway [109].

**Grade of Evidence: II**

### Whole Protein

The ingestion of whole protein, which is more commonly consumed in foods during prolonged steady state exercise (e.g., ultra-endurance) [15, 16], has also been previously investigated for the prevention and management of EIGS. Whey protein hydrolysate (i.e., 14.8 g protein/serve) consumed before and every 20 min during 2 h running at 60% *V̇*O _2max_ in 35 °C and 27% RH attenuated plasma I-FABP concentration and small intestine permeability, and subsequently resulted in a more beneficial bacterial endotoxin profile, although these differences in plasma endotoxin concentration were not significant [73, 117]. It was speculated that these beneficial outcomes were potentially due to increased heat shock protein expression and/or stabilization of tight-junction protein complexes. While whole protein may be beneficial to EIGS outcomes linked to the circulatory–gastrointestinal pathway, such high intakes increased Ex-GIS compared with ad libitum water [73], which has feeding tolerance and performance implications [17, 42]. Although large doses of protein during exercise may induce and/or exacerbate Ex-GIS, smaller doses (i.e., 3 g every 15 min) co-ingested with glucose appear to be tolerable [139]. However, the impact of lower protein doses during exercise on EIGS markers still warrants investigation.

**Grade of Evidence: II**

## Dietary Components and/or Interventions

### Gluten-Free

Adherence to a gluten-free diet is prevalent amongst non-celiac athletes, with the primary reason of self-diagnosis of gluten sensitivity symptom (i.e., gastrointestinal origin) or non-symptom related benefits (i.e., improves overall health and/or ergogenic effect) [140]. A gluten-free diet restricts gluten, a storage form composite of gliadins and glutenins commonly found in wheat, rye, barley, and triticale. Unlike celiac disease, at present there is no established clinical diagnostic biomarker (i.e., allergic or autoimmune response) for non-celiac gluten sensitivity [141, 142]. Nevertheless, non-celiac athletes have reported symptomatic improvements at resting and during exercise after implementing a gluten-free diet. Such symptomatic improvements may be due to the subsequent adjunct reduction of fermentable oligo-, di-, and mono-saccharides and polyols (FODMAPs), specifically fructans and galactooligosaccharides [143–146]. Other possible reasons are a change in dietary habits per se. For example, Lis et al. [140] reported that athletes increased their fruit, vegetable, and gluten-free wholegrain consumption when they switched to a gluten-free diet. To date, there is only one study that investigated the effects of a gluten-free diet versus a gluten-containing diet in non-celiac athletes on markers of EIGS and Ex-GIS. Non-celiac competitive cyclists found no overall effect of 7 days gluten-free versus gluten-containing diet on intestinal epithelial injury, systemic inflammatory cytokines, and Ex-GIS in response to an exertional stress experimental model [147]. At present, there is no evidence supporting a gluten-free diet in nonceliac athletes in the prevention and management of EIGS and Ex-GIS.

**Grade of Evidence: III**

### Fermentable Oligo-, Di-, and Mono-Saccharides and Polyols (FODMAP)

A low FODMAP diet is a well-recognized dietary intervention for managing diseases of gut–brain interaction (DGBI), namely irritable bowel syndrome (IBS)) [148–151]. Athletes who experience Ex-GIS often report similar symptom types as those with IBS; and commonly include flatulence, lower abdominal bloating and pain, and urge to defecate [3]. Many athletes often implement a low FODMAP diet pre-exercise as an effective strategy to reduce Ex-GIS [10, 11, 13, 152]. FODMAPs are rapidly fermentable short-chain carbohydrates, which are synonymous with increases in intestinal luminal gas, water, and metabolic by-products (e.g., short-chain fatty acids (SCFA)) [153, 154]. Collectively these biological changes result in luminal distension leading to lower-GIS symptoms in individuals with heightened visceral sensitivity [155]. Conversely, a low FODMAP diet is reported to reduce microbial diversity and total bacterial abundance [149], which may be counter productive to EIGS management. This is postulated to be due to diminished luminal content, fermentation, and SCFA production [29, 33]. Therefore, it is plausible that a lowered dietary intake of FODMAPs may support the management of Ex-GIS, but may negatively influence the pathophysiology of EIGS in susceptible athletes.

Owing to the increased energy requirements of athletes, the typical FODMAP dietary intake can be up to 81 g/day [144] compared with a typical westernized (i.e., Australian) diet of 24 g/day [153]. When adopting a low FODMAP diet (i.e., < 8 g/day of FODMAP), for either 6 days [11, 13, 152] or a short-term (i.e., 24–48 h) dietary intervention (i.e., ≤ 5 g/day) before exercise, [82, 156] a reduction in Ex-GIS severity before, during, and after exercise has been reported in response to exertional experimental models. Of interest, a 24-h low FODMAP diet before a substantial exposure to exertional-heat stress (i.e., 2 h running at 60% *V̇*O_2max_ in ~ 35 °C) showed greater intestinal injury along with a trend for a greater magnitude of bacterial endotoxin translocation, but did not impact systemic inflammatory responses compared with a high FODMAP diet. It has been proposed that the potential EIGS protective effects of a high FODMAP diet align with those mechanisms described with carbohydrate feeding before and during exercise in Sect. 4.1. In addition, they align with the increased SCFA concentrations observed in plasma and feces after high FODMAP diet adherence [28]. As discussed, there is evidence supporting the role of a short-term (i.e., 24–48 h) low FODMAP diet before exercise in reducing the severity of Ex-GIS, and there is also some evidence for a high FODMAP diet attenuating EIGS pathophysiology in athletes undertaking prolonged endurance-type exercise. Therefore, if implementing a short-term low FODMAP diet for the management of Ex-GIS severity, consideration must be given to other possible protective dietary influences on EIGS pathophysiology; namely, carbohydrate loading and/or carbohydrate feeding during exercise within tolerance levels.

**Grade of Evidence: I**

### Low Carbohydrate High Fat (Ketogenic Targeted Diets)

Maximizing fat as a substrate for endurance exercise, via a ketogenic low-carbohydrate high-fat (LCHF) diet, originated in the early 1980s and has since been thoroughly investigated, primarily in respect to fuel kinetics and exercise performance [157]. For athletes undertaking prolonged endurance-based exercise, it is theorized that a LCHF diet may facilitate extended durations of exercise performance without the need for frequent ingestion of carbohydrate-based fuels, which seems a positive outcome in respect to lowering the gastrointestinal burden during exercise, and subsequently favoring the abolishment of Ex-GIS. However, there is currently a scarcity of research focusing on gastrointestinal responses to the application of LCHF dietary interventions. Although enhancing the ability for endogenous fat oxidation at the expense of not having to or limiting the need for exogenous carbohydrate intake during exercise seems promising, several concerning aspects can be raised. Following dietary lipid consumption, such as a high-fat meal, circulating bacterial endotoxins (i.e., LPS) concentrations have been observed to increase in human experimental models [158], linking high-fat diets with increased bacterial endotoxin entry from the intestinal lumen into systemic circulation. In addition, an increased circulating concentration of I-FABP is also commonly reported in diets aimed at increasing lipid intake and oxidation [159]. Although I-FABP is indicative of intestinal epithelial cell damage, its levels are generally proportionally linked to the rate of cellular fatty-acid metabolism [160].

In an athletic population, an increase in plasma I-FABP concentration was observed following an acute 6 days LCHF diet (i.e., < 50 g/day carbohydrate, energy availability = 40 kcal/kgFFM/day) at rest and in response to prolonged strenuous exercise (i.e., 25 km race walk) in elite race walkers compared with a high-carbohydrate diet (i.e., 65% carbohydrate, energy availability = 40 kcal/kgFFM/day) [161]. In addition, the proposed compromised epithelial barrier before and during exercise was further supported by increased concentrations of soluble CD14 (sCD14) and lipopolysaccharide binding protein (LBP), both surrogate biomarkers indicative of luminal to systemic bacterial endotoxin translocation, in the LCHF group versus the high carbohydrate group. This likely influenced systemic inflammatory responses [162]. Despite evidence of increased injury and compromise to the intestinal epithelial barrier in athletes following a LCHF diet, incidence of Ex-GIS was observed, but did not significantly differ compared with the high carbohydrate diet. It is important to highlight that the higher FODMAP content of the high carbohydrate diet may have inadvertently exacerbated Ex-GIS incidence and severity via malabsorption and bacterial fermentation (as per Sect. 5.2), and subsequently masked potential effects of LCHF on Ex-GIS via the observed intestinal epithelial perturbations. In addition, from a professional practice perspective, following 32 weeks on a LCHF diet, a world-class long-distance triathlete experienced the worst-ever performance outcomes following half-ironman and ironman competitions. He experienced negative subjective well-being, and his usual gastrointestinal disturbances were not alleviated [14]. On the basis of the current evidence to date, LCHF dietary interventions do not support the prevention or management of EIGS and Ex-GIS.

**Grade of Evidence: II**

### Low Energy Availability

It is well-established that in medical conditions associated with acute or chronic periods of compromised nutritional intake (e.g., anorexia nervosa), gastrointestinal derangement and subsequent symptoms (e.g., gastro-esophageal and/or intestinal symptoms associated with disturbances to gastric motility, gastric emptying, and intestinal transit) are common manifestations [163, 164]. More recently, the exploration of athletes adhering to unmatched dietary and training regimes, synonymous with low energy intake, and/or high training and competition energy outputs has led to the greater knowledge and understanding of low energy availability (LEA), and the development of relative energy deficiency in sport (REDs) and its clinical consequences [165]. Clinical cases and exploratory research have revealed the multisystem impact of chronic LEA, including gastrointestinal disturbances and symptomatic outcomes potentially based on underlying neuroendocrine (e.g., altered and abnormal stress hormone responses) pathophysiology [166]. On the basis of these reports, it is plausible that individuals in a state of LEA and/or presenting diagnostic criteria for REDs may be more prone to EIGS and Ex-GIS as a result of the perturbed neuroendocrine–gastrointestinal pathway.

To date, only one study has investigated the effects of a short-term (i.e., 6 days) LEA intervention on gastrointestinal integrity biomarkers and Ex-GIS in elite race walkers [161]. Acute LEA (i.e., 65% carbohydrate, energy availability = 15 kcal/kgFFM/day) did not result in differing intestinal epithelial injury, bacterial endotoxin profile perturbations, or Ex-GIS compared with a high carbohydrate (i.e., 65% carbohydrate, energy availability = 40 kcal/kgFFM/day) diet over the dietary intervention period, in response to a 25 km race walk in temperate ambient conditions. Food and fluid were provided to mimic a race feeding regimen during exercise. It is possible that the short duration intervention may have resulted in these null effects, as longer-term LEA (i.e., timeline that impacts lean body mass and resting metabolic rate) results in more pronounced perturbations to physiological systems [165]. In addition, the provision of any carbohydrate dose during exercise may have also contributed to the lack of group differences [64, 73, 113–115]. Therefore, at this stage, it may be suggested that short-term LEA does not exacerbate EIGS or Ex-GIS; however, it is unknown if chronic LEA, leading to REDs, impacts how an individual gastrointestinal tract responds to exercise stress.

**Grade of Evidence: III**

### Low Fiber (and/or Residue)

It is well recognized that manipulating (i.e., increasing, reducing, or altering type) the fiber (e.g., insoluble: lignin and cellulose; and soluble: arabinoxylan, beta-glucan, guar gum, inulin, and psyllium) and/or other residues (e.g., resistant starches such as amylose) of dietary intake plays a key role in symptomatic management of DGBI (e.g., gastrointestinal functional disorders—IBS) and inflammatory diseases of the gastrointestinal tract (e.g., inflammatory bowel conditions such as Crohn’s diseases and ulcerative colitis) [167–172]. Considering the neuroendocrine–gastrointestinal and circulatory–gastrointestinal pathways of EIGS presents similar pathophysiological manifestations as these disease states, respectively; it seems logical that manipulating the intake of dietary residues (i.e., insoluble and soluble fibers, and resistant starches) before exercise may influence EIGS and subsequent Ex-GIS outcomes. From a theoretical perspective, the consumption of dietary residues before and during exercise may promote both increased gastric and intestinal content that may lead to a direct (i.e., in situ content) or an indirect (i.e., gas production and water translocation aligned with bacterial fermentation of soluble fibers and resistant starches) increase in luminal pressure leading to intestinal hypersensitivity, local dysmotility, and/or ileal brake mechanisms, contributing to Ex-GIS [43, 55, 57, 58]. Nevertheless, considering the fermentable capacity of soluble fibers and resistant starches by commensal bacteria along the gastrointestinal lumen, effects may mimic those reported in research of dietary FODMAP content (see Sect. 5.2) and/or prebiotic supplementation (see Sect. 6.2) on EIGS and Ex-GIS outcomes, whether it be exacerbating Ex-GIS severity and/or providing a beneficial effect on gastrointestinal integrity.

To date, only one retrospective field-based observational study has reported on the relationship between dietary fiber intake and Ex-GIS occurrence during competition in endurance athletes (i.e., long-course triathlon) [6]. The results were limited to those triathletes that presented with “intestinal cramps” also consumed fiber-rich foods in the pre-event meal. Despite these limited observational field research findings, it is surprising that a recent experimental survey (*n* = 277) found that endurance athletes (15.2%) self-reported following a low fiber diet in the management of Ex-GIS, which was the highest reported management strategy [9, 10]. In addition, the cohort reported lowering dietary fiber intake leading into competition was the most successful strategy in their perceived reduction in Ex-GIS. Current sports nutrition recommendations caution athletes to be wary of high-fiber foods prior to competition owing to the possibility of increasing the risk of developing Ex-GIS [173]. It is unclear where such therapeutic information and adherence is coming from, but speculation may possibly lie with unquestioned social acceptance stemming from clinical research in DGBI (i.e., functional diseases/disorders of the gastrointestinal tract) management [167, 168, 170, 171] and/or broad-spectrum sports nutrition recommendations. Nevertheless, low-residue intake, using low-fiber and elemental-based dietary components, formed part of EIGS and Ex-GIS management in a recent case series on endurance and ultra-endurance athletes experiencing severe Ex-GIS [12]. Outcomes from the individually tailored therapeutic interventions suggested including low-fiber and residue foods and beverages within the dietary regime of certain case athletes reduced Ex-GIS incidence and severity in subsequent competitive events, leading to these athletes reporting performance improvements (e.g., event completions and victories). It is worth highlighting that other therapeutic-focused interventions formed part of the overall management plan within the case series (e.g., gut training, low FODMAP diet, measured and planned carbohydrate intake during exercise, and euhydration without fluid overload, among others), and likely also contributed to the positive outcomes. Owing to anecdotal evidence in professional practice, it is speculated that adherence to low-fiber and/or residue diets may lower the predisposition to Ex-GIS [11]. However, considering that there is no experimental evidence on this topic, well-conducted randomized controlled trials are needed to provide full insight into the impact of dietary fiber on EIGS and Ex-GIS outcomes.

**Grade of Evidence: IV**

## Dietary and Nutritional Supplements

Various dietary and nutritional supplements have been explored in the context of attempting to ameliorate the pathophysiological pathways of EIGS, namely the circulatory–gastrointestinal pathway (i.e., intestinal epithelial cell and tight-junction injury and/or dysfunction, lumen to circulation pathogenic translocation, and systemic immune responses), and subsequently Ex-GIS. These include proposed antioxidants (i.e., ascorbic acid, tocopherol, and capsaicin); biotics (i.e., pre-, pro-, and syn-biotics), bovine colostrum, curcumin, anthocyanin, and nitrate.

### Antioxidants

Oxidative stress may contribute to the exacerbation of exercise-associated intestinal epithelial injury and subsequent local inflammatory responses [174, 175]. As such, the increased activity of intestinal epithelium reactive oxygen species, during exercise and/or in the recovery period after exercise (i.e., splanchnic reperfusion), may facilitate epithelial cell injury and tight-junction protein rupture or dysfunction, leading to enhanced translocation of pathogenic agents into systemic circulation [3, 37, 138]. It has, therefore, been proposed that supplementing with nutrients that present antioxidative or anti-inflammatory properties before exercise may ameliorate the associated gastrointestinal disturbances. To date, supplements containing antioxidative or anti-inflammatory properties, including ascorbic acid, tocopherol, and capsaicin have been investigated.

Using a test–retest model, an acute dose of L-ascorbic acid (1000 mg) 2 h before an incremental cycling test to exhaustion reduced circulating gram-negative endotoxin concentration compared with a control trial 8 weeks prior [176]. Given the lack of crossover, no placebo control, and extensive time between trials, it is unclear if the modestly reduced post-exercise endotoxin levels are directly related to acute ascorbic acid ingestion. Another investigation determined the impact of 2 weeks tocopherol or soy lecithin placebo supplementation on intestinal permeability, fecal blood loss, and GIS following a marathon [177]. There was no significant change in intestinal permeability with tocopherol supplementation in response to the marathon. Additionally, no significant difference in heme-positive fecal samples was observed (tocopherol: 10% versus placebo: 20%). While abdominal cramping and pain were significantly reduced with tocopherol supplementation, heme-positive fecal samples were unrelated to Ex-GIS.

It has also been proposed that an intervention involving the administration of an antioxidant rich compound, capsaicin, may be effective in preventing and/or managing gastrointestinal epithelial injury [178]. Capsaicin is a proposed antioxidant and anti-inflammatory compound found within hot peppers and has been shown to increase blood perfusion in the gastrointestinal epithelium, albeit within animal models [178]. Thus, an increase in blood perfusion from capsaicin consumption has been hypothesized to ameliorate gastrointestinal epithelial injury in humans. A randomized control trial undertaken to assess the effect of capsaicin supplementation on sprinting performance and IL-6 response implemented an intervention where participants received 25.8 mg of capsaicin immediately prior to performing 15 × 30 m sprints at 35 s intervals at maximum effort [179]. It was determined that participants experienced increased Ex-GIS with supplementation; however, IL-6 response was unchanged pre- to post-exercise [179]. Taken together, and considering the limited studies in the area that do not meet the minimal best practice recommendations, [2] there is no clear evidence to suggest a period of antioxidant supplementation before an exercise bout prevents or can be used to manage EIGS and/or Ex-GIS.

**Grade of Evidence: III to V**

### Biotics (Pre-, Pro-, and Syn-biotics)

It is a common belief among athletes and athlete support practitioners that a period of biotic supplementation, in the form of prebiotics (i.e., non-digestible material that can be fermented by bacteria in the lower gastrointestinal tract), probiotics (i.e., live bacteria which survives transit to colonize the lower gastrointestinal tract), or a combination referred to as synbiotics, will confer some beneficial effects to the gastrointestinal tract, particularly in response to exercise, when the gastrointestinal tract is compromised [180]. The mechanisms by which biotics may infer a beneficial effect on EIGS are associated with increasing the relative abundance of commensal bacterial along the gastrointestinal tract. This β-change may subsequently increase bacterial fermentation activity, leading to enhanced concentrations of luminal and/or plasma SCFA (e.g., acetate, butyrate, and propionate) [148, 181–183], newly termed post-biotics. Both fecal and plasma SCFA concentrations have been linked to protecting gastrointestinal integrity against exertional and exertional-heat stress [29, 33, 93]; although direct mechanistic explanation warrant further exploration and clarification.

A recent systematic literature review was undertaken to provide clarity on the impact of short or long-term biotic (i.e., pre-, pro-, syn-biotic) supplementation on markers of EIGS and Ex-GIS in response to exertional or exertional-heat stress [180]. However, no study that investigated the impact of prebiotic supplementation on EIGS and Ex-GIS was identified within the SLR. Recently, an 8-week prebiotic supplementation intervention (i.e., 16 g/day of a fructooligosaccharides, galactooligosaccharides, resistant starch, and dietary fiber formulation) prior to a 3 h exertional-heat stress experimental protocol resulted in a noticeable reduction in intestinal epithelial injury and luminal to systemic bacterial endotoxin translocation. However, it had no impact on attenuating systemic inflammatory response and did not influence gastrointestinal functional responses [184]. Additionally, the prebiotic supplementation did not further exacerbate Ex-GIS severity compared with the non-prebiotic-containing and low FODMAP placebo. It is important to note that the observed beneficial outcomes on ameliorating intestinal epithelial injury and bacterial endotoxin translocation with prebiotic supplementation were modest in nature, and overall exertional-heat stress associated gastrointestinal integrity perturbation were lower than previously reported [67]. This is likely attributed to the attenuating effects of carbohydrate feeding, as described in Sect. 4.1, which was provided in the first 2 h of the protocol.

Within the systematic literature review, probiotic and synbiotic supplementation interventions included single or multiple species/strains: *B. animalis*, *B. bifidum*, *B. breve*, *B. lactis*, *B. longum, B. subtilis*, *E. faecium*, *L. acidophilus*, *L. brevis*, *L. casei*, *L. fermentum*, *L. helveticus*, *L. lactis*, *L. paracasei*, *L. plantarum*, *L. rhamnosus*, *L. salivarius*, and/or *S. thermophilus*, with or without fructooligosaccharides (55.8 mg/dose) or inulin (2.3 g/dose). Bacterial doses ranged between × 10^8^ to × 10^11^ colony forming units, with supplementation period ranging from 7 days to 3 months. Exercise protocols varied from an incremental cycling test to exhaustion, to an ultra-endurance triathlon event, as well as a military training protocol. Probiotic or synbiotic supplementation did not present any substantial beneficial effect compared with placebo or control on surrogate markers of intestinal epithelial injury. Inconsistent outcomes were observed with assessment markers of intestinal permeability with probiotic or synbiotic supplementation; with higher, lower, and no difference in outcomes between supplementation and placebo were reported. Of note, the reported beneficial effects of synbiotic supplementation on intestinal permeability was as a result of using the erroneous zonulin biomarker to quantify permeability [185–188]. No probiotic or synbiotic supplementation intervention reduced markers of exercise-associated endotoxemia compared with the study’s respective placebo or control. However, one intervention (seven days *L. casei*) reported a substantial increase in gram-negative bacterial endotoxin plasma concentration in response to 2 h steady-state treadmill running (60% *V̇*O_2max_) in hot ambient conditions (34.0 °C, 32% RH) compared with a modest reduction in the placebo group [189]. The increased endotoxemia was speculated to be associated with a greater bacterial luminal load as a result of the 7 days *L. casei* supplementation dose (i.e., more bacterial endotoxin in the lumen available to translocate across the exercise-associated compromised epithelial barrier). There was no consistency in the impact of probiotic or synbiotic supplementation on systemic inflammatory responses, with no substantial differences reported in included studies. Regarding Ex-GIS, four out of the five studies within the SLR presented data indicative of no effect on Ex-GIS incidents and/or severity. One study reported lower Ex-GIS severity in the probiotic supplement group (i.e., 4 weeks, > 25 billion CFU/day from *L. acidophilus CUL60, L. acidophilus CUL21, B. bifidum CUL20, B. animalis subsp. Lactis CUL34*) in response to a simulated marathon. However, at closer inspection of the data and considering the methodological issues, it can be argued that the placebo group outperformed the probiotic supplementation group in regards to incidence of Ex-GIS and GIS in recovery from exercise. No study to date has assessed the impact of biotics on markers of gastrointestinal function. It was not surprising that no substantial differences were observed for EIGS markers, considering probiotic and synbiotic supplementation did not result in any changes in fecal bacterial composition (i.e., α-diversity and/or relative abundance), with only the supplemented strain or species showing increases in relative abundance. These increases, however, did not translate into increases in SCFA. The SLR concluded that probiotic supplementation with the strain or species studied does not substantially influence intestinal injury and permeability, subsequent systemic endotoxin or inflammatory cytokine responses, or GIS in response to exercise. As reported in the risk of bias assessment, many studies lacked adequate exertional and/or heat stress, or appropriate spectrum of biomarkers, to make definitive conclusions. Synbiotic supplementation appears to closely resemble the effects of probiotic, rather than prebiotic supplements, owing to the generally very small quantity of prebiotic ingredients included within the study intervention formulation.

**Grade of Evidence: I to II**

### Bovine Colostrum

Bovine colostrum has been proposed to support the gastrointestinal system via promotion of villus development and mucosal thickness throughout the gastrointestinal tract [190]. As reported in animal experimental models, evidence suggests that bovine colostrum protects against intestinal hyperpermeability associated with non-steroidal anti-inflammatory drugs (NSAIDS) or hyperthermia [191, 192]. It is therefore plausible that bovine colostrum supplementation may contribute towards a management strategy for EIGS. Several randomized controlled trials have investigated the effect of acute and prolonged bovine colostrum supplementation on markers of EIGS, with conflicting findings reported in regards to intestinal epithelial injury and permeability outcomes in response to exercise. A wide variety of exertional stress protocols have been used, ranging from 20 min running at high intensity (up to 80% *V̇*O_2max_) to 1.5 h combined cycling and running, and within diverse environmental conditions (i.e., ambient temperature ranging from 22 to 40 °C) [193–200]. Supplementation protocols included ingestion of bovine colostrum for 14 days at 20 g/day with or without zinc carnosine, [193–196] 8 weeks at 60 g/day, [199] and 7 days at 1.7 g/kgBM, [198] before the respective exercise protocol. The proposed protective effects of bovine colostrum were assessed via urinary L:R ratio for intestinal permeability, plasma I-FABP concentrations pre- to post-exercise, and exercise-induced systemic inflammatory response (i.e., cytokine profile). Research outcomes appear inconsistent, with some studies reporting modest positive effect with 14 days of supplementation at 20 g/day supplementation on mitigating L/R and/or the rise in I-FABP from pre- to post-exercise, compared with placebo [193–196]. However, other studies, using more substantial exercise models with or without the addition of heat exposure (e.g., 1.5 h cycling and running and 45 min exercise in 40 °C) demonstrated no beneficial effects of supplementation on markers of intestinal permeability or systemic inflammatory response [197–200]. Previous arguments in support of bovine colostrum in protecting the gastrointestinal tract (i.e., intestinal epithelial injury and permeability) in response to exercise are acknowledged [201]. However, several supplement intervention studies, with closer adherence to best practice recommendations for research methodologies, [67] have shown no beneficial outcomes compared with respective placebo or control [197–200]. In addition, a systematic literature review with meta-analysis that included exploration of measures related to EIGS (i.e., circulating immunoglobulins and leukocytes) reported none to fairly low impact of bovine colostrum supplementation intervention on these biomarkers [202]. Taken together, the current evidence suggests there is no strong and consistent evidence to recommend acute or prolonged supplementation with bovine colostrum to attenuate disturbances to the gastrointestinal integrity and/or systemic inflammatory responses associated with EIGS.

**Grade of Evidence: II–III**

### Curcumin

Curcumin, the primary compound found in turmeric, is of interest owing to its anti-inflammatory properties and proposed function in strengthening intestinal endothelial tight junctions, demonstrated both in vitro and in vivo [203–205]. It also appears to attenuate proinflammatory LPS signaling pathways, moderating disturbance to gastrointestinal epithelial lining and resulting in reduced bacterial translocation. This potentially reduces systemic inflammatory responses [204, 205]. To date, only one study has investigated the potential role of curcumin in moderating markers synonymous with EIGS. It was observed that participants who supplemented with 500 mg/day of curcumin for 3 days had a significantly smaller increase in plasma I-FABP (absolute difference ~ 366 pg/mL) and IL-1ra (absolute difference ~ 8 pg/mL) after 60 min of moderate-intensity running in 37 °C ambient temperature compared with those who supplemented with placebo [206]. Unfortunately limitations, including insufficient exercise stress to induce substantial elevations in relevant biomarkers, confirmed by a rise in I-FABP under the minimal detectable change (MDC), [33] make it challenging to conclude if differences were of clinical and practical relevance. Therefore, more methodologically robust studies are required to unveil the preventative or mitigating potential of curcumin supplementation on EIGS. Given that limited research is available, it is currently not recommended as a first-line action for athlete application as a strategy for reducing EIGS.

**Grade of Evidence: III**

### Anthocyanins

Anthocyanin, a bioactive flavonoid polyphenol, has been proposed to attenuate nuclear factor-kappa B (NF-κβ)-mediated inflammatory responses, including targeted effects on protecting gastrointestinal barrier integrity in human experimental models [207–210]. Moreover, ingestion (240 mg) of blackcurrant extract that contains a substantial anthocyanin dose (e.g., cyanidin-3-O-glucoside, cyanidin-3-O-rutinoside, delphinidin-3–100 O-glucoside, and delphinidin-3-O-rutinoside), [211] before exertional stress (i.e., 30 min rowing at 80% *V̇*O_2max_), was reported to mitigate oxidative stress (e.g., plasma carbonyls), and inhibit LPS-stimulated cytokine secretion (i.e., TNFα and IL-6) and NF-κβ activation, compared with placebo [212]. It is therefore plausible that such supplementation before exercise may provide some attenuating effects on markers respective of exercise-associated gastrointestinal integrity perturbations. A recent study investigated the effects of 7 days of anthocyanin-rich blackcurrant extract administration (600 mg/day) on gastrointestinal integrity markers in response to exertional-heat stress (i.e., 60 min running at 70% *V̇*O_2max_ in 34 °C and 40% RH ambient conditions) [213]. Although, the supplementation intervention led to a significant reduction in intestinal epithelial injury (i.e., plasma I-FABP concentration) after exertional-heat stress compared with placebo, no differences were observed for small intestinal permeability (i.e., 4 h urinary L:R ratio), bacterial endotoxin translocation (i.e., sCD14 and LBP), or an array of systemic inflammatory responses biomarkers (i.e., IL-6, IL-10, and IL-1ra). Similar to other nutrition supplement studies attempting to manage exercise-associated perturbation to gastrointestinal integrity, it is important to note that the indication of overall intestinal epithelial injury, bacterial endotoxin translocation, and inflammatory cytokine biomarker values, in both the anthocyanin and placebo trials, were modest in nature. In addition, they were under the MDC previously proposed to warrant practical and clinical significance; which was likely associated with the modest exertional-heat stress model adopted [33, 67]. Taken together, it appears that the positive effect of acute intake of anthocyanin on in vitro pathogen challenge and inflammatory responses are not supported by a more prolonged supplementation period and its impact on in vivo biomarkers within human trials. In view of the current limited research available and the need for more robust experimental designs (e.g., more prolonged exertional-heat stress models to test supplementation hypothesis and adequate confounder control), it is currently not recommended as a first-line action for athlete application as a strategy for reducing EIGS.

**Grade of Evidence: III**

### Nitrate

Gastrointestinal perfusion is dependent on macro- and micro-vascular activity throughout the splanchnic vasculature network, in which nitric oxide is a key regulator, inducing vasodilation [109, 214]. It can be proposed that increasing local availability of nitric oxide through nitrate supplementation may play a role in preventing and/or attenuating exercise-associated splanchnic hypoperfusion, and subsequent local ischemia. To date, only one study has investigated the effects of a nitrate-containing beverage (800 mg) on markers of splanchnic perfusion and ischemia, epithelial integrity, and Ex-GIS compared with carbohydrate (20 g sucrose) and water trials, in response to 60 min cycling at 70% *W*_max_ [115]. Participants were provided with a supplement dose 15 min pre-exercise and halfway through a 60 min cycle at 70% *W*_max_. Despite a clear increase in plasma nitrate and nitrite, supplementation did not improve splanchnic perfusion, epithelial ischemic injury, or bacterial endotoxin translocation. Compared with water, nitrate supplementation resulted in a 50% increase in intestinal epithelial injury (i.e., plasma I-FABP concentration), whereas sucrose ameliorated intestinal epithelial injury. The percentage change in plasma I-FABP concentration pre- to post-exercise with a nitrate (299%) beverage was considerably larger compared with sucrose (179%). Ex-GIS were similar between groups. It appears that nitrate supplementation does not dampen, and may even exacerbate, intestinal epithelial injury during moderate duration exercise (≤ 1 h) when compared with water. However, it is important to note that the exercise model used within this study was insufficient to warrant substantial gastrointestinal perturbations and associated Ex-GIS of any relevance, [33] and thus no ideal experimental design has been undertaken to appropriately test nitrate supplement intervention in regard to EIGS mitigation or exacerbation [67].

**Grade of Evidence: III**

## Hydration

The ingestion of water, via an array of fluid types, during prolonged exercise (e.g., ≥ 1 h), especially when performed in hot (≥ 35 °C) ambient conditions, is important to maintain euhydration and avoid adverse physiological consequences of hypohydration, which may lead to exercise performance decrements [101, 215]. Current water replacement guidance for sport and exercise modalities are substantially affected by fluid shifts (e.g., endurance and ultra-endurance), and they suggest fluid intake should be individually tailored using either ad libitum or planned fluid intake strategy to avoid adverse consequences associated with both hypohydration and overhydration (i.e., exercise-associated hyponatremia) [101, 173, 216, 217]. Considering the naturally large intra- and inter-individual variation in sweat rates in response to exercise, there is a potential risk for both under- and over-hydration to impact the gastrointestinal tract leading to GIS [218–220]. For example, fluid intake above individual gastric tolerance levels will increase intragastric pressure, causing gastric distension and contributing to the development of upper-GIS symptoms (e.g., upper abdominal bloating and/or pain, belching, urge to regurgitate or regurgitation) [41–43]. In contrast, hypohydration may exacerbate EIGS through reductions in plasma volume that enhance splanchnic hypoperfusion and increases in sympathetic drive that may suppress gastrointestinal functional responses [41]. Thus, the maintenance of euhydration in response to exercise would seem a plausible strategy to prevent or ameliorate EIGS and Ex-GIS.

### Pre-Exercise Hydration

Commencing exercise at 3.0% body mass loss via pre-exercise sauna exposure slowed gastric emptying during 90 min cycling at 70% *W*_max_ compared with starting exercise euhydrated [221]. Orocecal transit time (OCTT) and post-exercise small intestine permeability were similar between pre-exercise euhydration and hypohydration where fluid was ingested during exercise, although prior hypohydration resulted in greater Ex-GIS (i.e., nausea and upper abdominal pain) [221]. Moreover, pre-exercise hypohydration induced by limiting fluid intake to 0.5 L/day prior to 1 h cycling at 70% *W*_max_, with no fluid intake during exercise, resulted in modestly higher intestinal epithelial injury (i.e., I-FABP: + 300 pg/mL, no statistical analysis presented) compared with pre-exercise euhydration achieved by habitual fluid intake [222]. On the basis of these findings, and considering the difficulties in consuming large volumes of fluid during certain exercise activities [173, 216, 217], it is recommended that individuals commence exercise activities in the euhydrated state. However, caution is warranted, as excessive pre-exercise fluid ingestion in the attempt to obtain euhydration may promote Ex-GIS, likely associated with increased gastrointestinal load. For example, fluid ingestion (1134 mL) immediately pre-exercise significantly increased the severity of exercise-related transient abdominal pain during the first 5 min of running compared with no fluid ingestion [223]. In addition, although the impact of hyperhydration status on markers of EIGS and Ex-GIS is unknown and warrants exploration, some of the strategies used to promote hyperhydration (e.g., glycerol supplementation and sodium intake) have been reported to instigate GIS (supplementary file 2) [224].

**Grade of Evidence: II**

### Hydration During Exercise

In a recent study, an increase in post-exercise plasma I-FABP concentration was accompanied by varying magnitudes of increased systemic inflammatory cytokines with fluid restriction during 2 h running at 70% *V̇*O_2max_ (3.1 ± 0.7% body mass loss and *P*_Osmol_ > 300 mOsmol/kg) in temperate conditions when compared with programmed fluid ingestion rates that maintained euhydration (0.6 ± 0.6% body mass loss and *P*_Osmol_ < 300 mOsmol/kg) [41]. Hypohydration resulted in higher incidence and severity (82% and 240 mean summative accumulation during exercise, respectively) of total Ex-GIS compared with euhydration (64% and 176 mean summative accumulation during exercise, respectively). However, it is important to highlight that no significant difference was observed between the hypohydration and euhydration trials for Ex-GIS variables (i.e., trend at *p* = 0.058 and *p* = 0.068 for lower-GIS and lower abdominal pain, respectively). This can possibly be attributed to the large individual variation common to GIS reporting and/or that participant numbers were underpowered for such a subjective variable. Moreover, a separate study focused on ad libitum water intake during exercise (i.e., 2 h running at 60% *V̇*O_2max_ in temperate ambient conditions) to maintain euhydration. Although Ex-GIS incidence was similar (70%), symptom severity was much lower (58 mean summative accumulation during exercise), possibly suggesting that fluid intake behavior may impact Ex-GIS outcomes (i.e., forceful intake via programming versus ad libitum intake within comfort) [71]. Nevertheless, carbohydrate malabsorption, as determined by breath H_2_, of the high-mixed carbohydrate meal given 2 h before exercise was pronounced with hypohydration, but not with euhydration; this likely explains the trend towards greater lower-GIS in the hypohydration trial compared with euhydration trial [41].

In another study, 1 h running at 70% *V̇*O_2max_ without fluid provisions significantly increased intestinal permeability compared with pre-exercise values; this was not significantly dampened by the provision of a carbohydrate beverage or a no-carbohydrate placebo beverage [225]. However, exercise-induced body mass loss was only 1.5%, likely linked to the modest exertional stress model used, which is not indicative of exercise-associated dehydration. Fluid provision during exercise resulted in greater feelings of fullness, but other GIS types were not affected. The optimal timing and volume of fluid provisions during exercise to reduce EIGS and Ex-GIS remains to be determined, but it is likely to be individualized [12]. Additionally, the effectiveness of fluid provision to reduce perturbations to gastrointestinal integrity may be dependent upon exercise duration or the extent of exercise-induced hypohydration. Considering hypohydration and excessive fluid intakes may contribute to EIGS and/or Ex-GIS, individuals should aim to establish a balance between individual tolerance levels to fluid intake during exercise and adequate fluid intake during prolonged exercise (> 1 h) to aid the maintenance of euhydration throughout.

**Grade of Evidence: II**

## Heat-Mitigating Strategies

The rise in core body temperature during exercise as a result of skeletal muscle function and metabolism, exacerbated further by hot and/or humid conditions, is directly positively correlated with increased perturbations to gastrointestinal integrity, systemic responses, and Ex-GIS [83]. A recent meta-data analysis from *n* = 132 exertional and exertional-heat stress trials suggested that maximal core body temperature accounted for 16.4%, 24.9%, 42.4%, and 12.4% of the predictive variance in the magnitude of epithelial injury, systemic endotoxemia and inflammatory response, and total-GIS, respectively [83]. Moreover, core body temperatures of ≥ 39.5 °C consistently elicit intestinal epithelial injury, bacterial endotoxin translocation, systemic inflammatory responses, and Ex-GIS over minimal detectable change synonymous with clinical relevance [33, 83]. Heat mitigation strategies, such as heat acclimation/acclimatization, pre- (before exercise) and per- (during exercise) cooling, using internal (e.g., cold beverages or ice slurry) and external strategies (e.g., ice-vest, cold water immersion, or cooling showers), and fluid ingestion (covered in the hydration section) may attenuate the rise in core body temperature during exercise in the heat and therefore may minimize EIGS and Ex-GIS [226].

### Heat Acclimation/Acclimatization

Heat acclimation/acclimatization (e.g., prolonged and repeated exposure to heat stress) appears to be an effective extrinsic heat mitigation strategy [226]. However, research to date is not supportive of its efficacy at mitigating EIGS. Intestine permeability was not different in response to a 45 min run at 50% *V̇*O_2max_ in 46 °C (20% RH) following 7-day (100 min exertional-heat stress) heat acclimation [227]. Permeability was not increased from rest and was measured by lactulose ingestion and excretion only, therefore making it difficult to interpret small intestine permeability outcomes. This study, however, showed a reduction in IL-6 and IL-10 cytokines that coincided with reduced thermoregulatory and cardiovascular strain. Further studies have also demonstrated that 5–10 days of heat acclimation (i.e., running in 41.4 °C, until a + 2 °C core body temperature increase and 60 min cycling at 50% *V̇*O_2peak_ in 35 °C and 50% RH or 40 °C and 25% RH) have been insufficient at attenuating I-FABP, gastric emptying rate, LPS, or the cytokine profile in response to heat stress or hypoxic (F_i_O_2_ = 0.14, 40 min cycle at 50% *V̇*O_2peak_) exercise [228–230]. Considering the proposed benefits of heat acclimation/acclimatization at attenuating cardiovascular and thermoregulatory strain during exercise in the heat [226], further research with heat acclimation regimens of more prolonged exposure (e.g., > 10 days) and sufficient adaptive stimulus (e.g., ≥ 90 min/exposure) are required to elucidate if this is an effective strategy for attenuating EIGS and Ex-GIS.

**Grade of Evidence: III**

### Internal Cooling

Internal cooling methods include the ingestion of cold fluids or ice slurry before (pre-cooling) and/or during (per-cooling) exercise. Internal cooling can lower pre-exercise core body temperature (e.g., when ingested prior to exercise) and create a heat sink that enables greater heat storage capacity, and subsequently, improved exercise performance in the heat [226]. Therefore, internal cooling methods may attenuate EIGS through directly lowering peak core body temperature and/or delaying the rise in core body temperature during exercise and heat stress load [83] with respect to exercise intensity and duration, ambient conditions of exercise adherence, and other thermoregulatory modifiers (e.g., air flow, clothing, and/or equipment). Internal per-cooling research has shown a suppressed rise in core body temperature (mean reduction of 0.3–0.4 °C) with water bolus ingestion with 0 °C and 7 °C water temperature compared with 22 °C water temperature given every 15 min during 2 h running at 60% *V̇*O_2max_ in 35 °C (25% RH). This resulted in modestly attenuated I-FABP and lower incidence (i.e., 67% versus 83%) and severity (i.e., 129 versus 235 summative accumulation) of upper-GIS symptoms; however, they did not reach statistical significance (i.e., trend at *p* = 0.066 and *p* = 0.087), respectively [231]. Additionally, no difference in cytokine profile was reported. These modest benefits with lower drink temperatures on intestinal injury and/or Ex-GIS (i.e., incidence and severity) are likely attributed to the modest suppressed rise in core body temperature; it can be speculated that it could potentially have been more beneficial if greater difference in core body temperature were observed [71, 83]. Further research has shown that ingestion of 617 g of a sports drink ice slurry during the cycle leg of a simulated Olympic distance triathlon in 32–34 °C (20–30% RH) attenuated intragastric body temperature and was well-tolerated, but no other markers of EIGS were reported [232]. One recent study reported no difference in markers representative of disturbed gastrointestinal integrity (i.e., I-FABP and LPS) with the frequent (i.e., pre-exercise and every 15 min) provisions of a carbohydrate–electrolyte ice slurry and respective carbohydrate–electrolyte beverage at ambient temperature during 45 min running at 60% *V̇*O_2peak_ that was followed by a second run until volitional exhaustion at 70% *V̇*O_2peak_ [233]. The research protocol of this study provided carbohydrates during exercise, which is aligned with attenuating exercise-associated disturbances to gastrointestinal integrity, as previously discussed in Sect. 4.1. Therefore, it is not surprising that no intervention outcomes were observed, since the study overall negated its primary research focus owing to this erroneous protocol oversight (i.e., carbohydrate provisions before and during exercise) in assessing the impact of internal per-cooling on markers of gastrointestinal integrity [67]. To date, internal cooling with cold water and ice slurry during exercise appears to be well-tolerated and contributes to suppressing the rise in core body temperature, which has positive associations and can predict the magnitude of EIGS and Ex-GIS [83]. Indeed, additional research using more robust experimental designs and control, ensuring that greater difference in core temperatures are achieved, and employing a wide array of EIGS markers is warranted.

**Grade of Evidence: III**

### External Cooling

External cooling methods consist of the application of a cold medium such as ice vests/packs, cold towels, or cold-water immersion. These methods can reduce pre-exercise core body temperature and increase the core to skin temperature gradient [226]. Similarly to internal cooling, external cooling strategies may attenuate EIGS through directly lowering peak core body temperature and/or delaying the rise in core body temperature during exercise and heat stress load. Research on the gastrointestinal responses to external cooling methods is limited to systemic inflammatory cytokine responses, with one pre-cooling study demonstrating a small attenuation of IL-6 and IL-10 responses with 60 min cold water (20 ºC) immersion before 90 min of running at 65% *V̇*O_2max_ in 32 ºC (47% RH) [234]. While another study demonstrated no difference in IL-6 with application of an ice vest and cold towel before 30-min intermittent sprint exercise in the heat [235]. Further exploration into external pre- and per-cooling methods in isolation and in combination with internal cooling methods is warranted and may provide a more thorough insight into how cooling strategies may directly or indirectly impact markers of gastrointestinal integrity or function, systemic responses, and subsequent Ex-GIS. In addition, despite extensive research into the impact of cold (i.e., ≤ 0 °C) exposure, with and without exercise, on many physiological variables [236–239], the direct impact of external extreme cold stimulus and/or states of hypothermia on the gastrointestinal tract is still relatively unknown and warrants exploration.

**Grade of Evidence: III**

## Gut Training

The idea of a highly adaptable gastrointestinal tract was first proposed in the 1990s [240]. Training the gut through constant exposure to nutrition around exercise is proposed to improve its function and thus an individual’s tolerance to feeding during exercise, subsequently reducing the risk of Ex-GIS [241]. With the first few proof-of-concept studies in athletes only published in recent years, gut training remains an understudied topic.

### Repetitive Feeding Challenge

The various approaches to gut training target the gastrointestinal tract’s adaptive potential that may occur with this strategy, and its relevance in managing EIGS and/or Ex-GIS have been explored through a SLR [241]. First, increasing the stomach’s capacity and tolerance to larger gastric content could reduce the sensation of fullness. The ability for gastric adaptation to food intake volume is observed anecdotally or in non-exercise settings (e.g., competitive speed eating contests and eating disorders) [242–244]. Gut comfort and/or Ex-GIS improved among runners through daily repetitive ingestion of nutrients (90 g/h 2:1 glucose:fructose 10% w/v) and a high fat supplement (33 g fat, 9 g protein, and 9 g CHO per hour) during exercise for 1–2 weeks [42, 43, 106], and repeated ingestion of sweat rate-matched fluids [245]. Second, it is proposed that gut training can increase gastric emptying. Despite the existing evidence on nutrient-specific changes in gastric emptying in non-athletic human populations [246–248], this has yet to be proven within an exercise model. Third, a higher carbohydrate intake both during exercise and/or overall daily intake can increase intestinal absorptive capacity. Preliminary evidence in animal models showed nutrient-specific upregulation of intestinal transport proteins through taste-transduction pathways (e.g., type 1 taste G protein-coupled receptors (GPCRs), α-gustductin) [249–251].

Additionally, the increase in nutrient absorption, gastric emptying, and gastrointestinal motility may also be improved through the inhibition of the ileal break mechanism [55, 252, 253]. This is only indirectly supported by existing gut-training studies looking at breath H_2_ concentrations, [42, 43, 65] and carbohydrate availability and oxidation [42, 254]. Specifically, carbohydrate malabsorption to a 90 g/h 2:1 glucose:fructose gut-challenge was reduced after gut training (i.e., 2 weeks of daily repeated feeding-challenge), but not on matched placebo [42, 43]. These findings were speculated to be caused by improved intestinal carbohydrate absorption, as supported by higher blood glucose concentration during exercise post-gut training, but not on matched placebo. This increased carbohydrate availability seen in Costa et al., [42] was not observed by increasing daily dietary carbohydrate intake for 28 days [254]. Moreover, carbohydrate malabsorption was not observed to any substantial relevant level (i.e., breath H_2_: 5 ppm before gut training and 4 ppm post-gut training) in response to a formulated 87 g/h glucose feeding challenge during exercise [106, 255]. This suggests that fructose may be the prime culprit in the malabsorption observed in the initial trial of Costa et al. [42], irrespective of co-ingestion with glucose, and subsequent improved fructose absorption in the post-gut training trial (also see supplementary file 1, carbohydrate type). From a professional practical perspective, such outcomes have been observed in athlete support intervention (i.e., case study). In these, higher carbohydrate doses with the inclusion of fructose (25 g) result in greater carbohydrate malabsorption (10 ppm breath H_2_) versus lower total carbohydrate and fructose (12.5 g) dose (6 ppm breath H_2_) [64]. Lastly, the impact of a repetitive feeding challenge on intestinal epithelial integrity (i.e., plasma I-FABP concentration) has been explored, but no effects have been observed. There was a substantial amount of individual variation within studies for intestinal epithelial integrity markers, likely associated with differences in nutrient (e.g., carbohydrate) content and processing along the gastrointestinal tract during the experimental procedures [42, 65], as discussed in Sect. 4.1. Overall, the potential adaptations to gastrointestinal accommodation, motility, and absorption could lead to less Ex-GIS and better tolerance of nutrition during exercise, and subsequent enhanced exercise performance outcomes, as previously observed [42, 43]. To date, there is some evidence to support repetitive gut-challenge protocols in the prevention and management of EIGS and Ex-GIS [12, 241]. However, it appears to mainly benefit carbohydrate malabsorption and associated Ex-GIS, with more advanced gastrointestinal functional measures still warranting investigation and no impact on gastrointestinal integrity evident.

It is also important to highlight that intermittent fasting is now a common practice in the sporting community with the aim of modifying body composition without performance decrements [256]. Considering the potential physiological and biochemical adaptations associated with gut training are at the expense of substantial and constant nutrient provisions, it is unknown whether such fasting practices and subsequent reductions in nutrient exposure are counterproductive in regard to gastrointestinal functional responses, Ex-GIS, and their performance implications. Nevertheless, from a gastrointestinal and professional practice perspective, it appears that overall carbohydrate intake tolerance in the general active population equates to a liquid form at ≤ 1.0 g/kgBM/h in 6–10% w/v with a simple glucose formulation [42, 54, 66, 73, 117]. Greater carbohydrate volumes and concentrations and/or altered forms (e.g., other and/or multiple carbohydrate forms, semisolid to solid textures, and/or inclusion of other substances such as caffeine) appear to be at the discretion of individual tolerance and/or gut training to improve formulation specific tolerance (see supplementary file 1).

**Grade of Evidence: I**

### Non-caloric Sweeteners

Similar to the mechanisms proposed for repetitive feeding challenge, consumption of non-caloric sweeteners may play a role in enhancing intestinal glucose uptake through the upregulation of active (i.e., SGLT1) and passive (GLUT5) transporters at the enterocyte apical surface, with or without upregulating GLUT2 at the basolateral surface, although this has primarily been observed in animal models [250, 257]. As such, enhancing glucose absorption and reducing the overall nutrient residue along the gastrointestinal tract may be linked to reducing Ex-GIS. Only one study to date has investigated the effects of sucralose supplementation (1 mM every 15 min for 2 h) prior to 2 h cycling at 48% *V̇*O_2peak_ [258]. No difference in overall Ex-GIS was observed in response to a carbohydrate challenge (1.2 g/kg/h maltodextrin) during exercise between the sucralose and control trials. While the exercise stress was insufficient to warrant any substantial gastrointestinal disturbance [67], it is important to note that GIS were only measured post-exercise retrospectively.

**Grade of Evidence: III**

### Fluid Tolerance Training

Considering the plasticity of the stomach chamber to volume, repeated exposures to ingesting large fluid volumes (e.g. > 800 mL/h) may effectively improve stomach emptying rate and subsequent comfort. Ingesting a 4% w/v carbohydrate–electrolyte beverage every 10 min during running to match sweat losses (830 mL/h) increased stomach fullness from 2 (2.0: comfortable) to 2.3 (3: moderately uncomfortable), when compared with ingesting the beverage ad libitum (338 mL/h) [245]. A total of five repeated trials (every 7–11 days) of 90 min running at 65% *V̇*O_2max_ with volumes to match sweat loss significantly improved stomach comfort. Gastric emptying assessed on the first and fifth run remained unchanged, indicating that the improvement may be related to sensory factors and tolerance (e.g., desensitization) in coping with increased intragastric pressure.

**Grade of Evidence: III**

## Translation to Practice

Considering that intervention research shows large individual variation in assessment markers synonymous with EIGS and factors exacerbating Ex-GIS in athletes [3, 67, 259], there is no one standard approach and therapeutic intervention for EIGS and Ex-GIS prevention or management. This means practitioners should be cautioned against providing generalized prevention and management strategies of EIGS and EX-GIS, as each individual athlete case is different and unique. To determine the most effective strategy in prevention and management, an individualized assessment to establish the underlying causes and exacerbators of EIGS and Ex-GIS is of vital importance. Supplementary file 3 covers practical aspects of the management of EIGS and Ex-GIS in sports and exercise clinical practice, elite sports performance, and field based competitive events. Context acknowledges and highlights individualization in the prevention and management pathway [12, 75], as well as other underlying factors from an individual athlete perspective and sports-specific training or competition aspects, which may promote exacerbation of EIGS and subsequent Ex-GIS. These can include predisposition, established diseases/disorders, or other underlying health influencing behaviors that may alter gastrointestinal integrity and/or functional response to exercise [165]. It can include training or competition aspects relating to pathogen exposure and contamination (i.e., environmental, population contact, and/or equipment cross-contamination) that may induce acute or chronic gastrointestinal-associated illness or infection [260].

## Summary

**Fig. 2:**
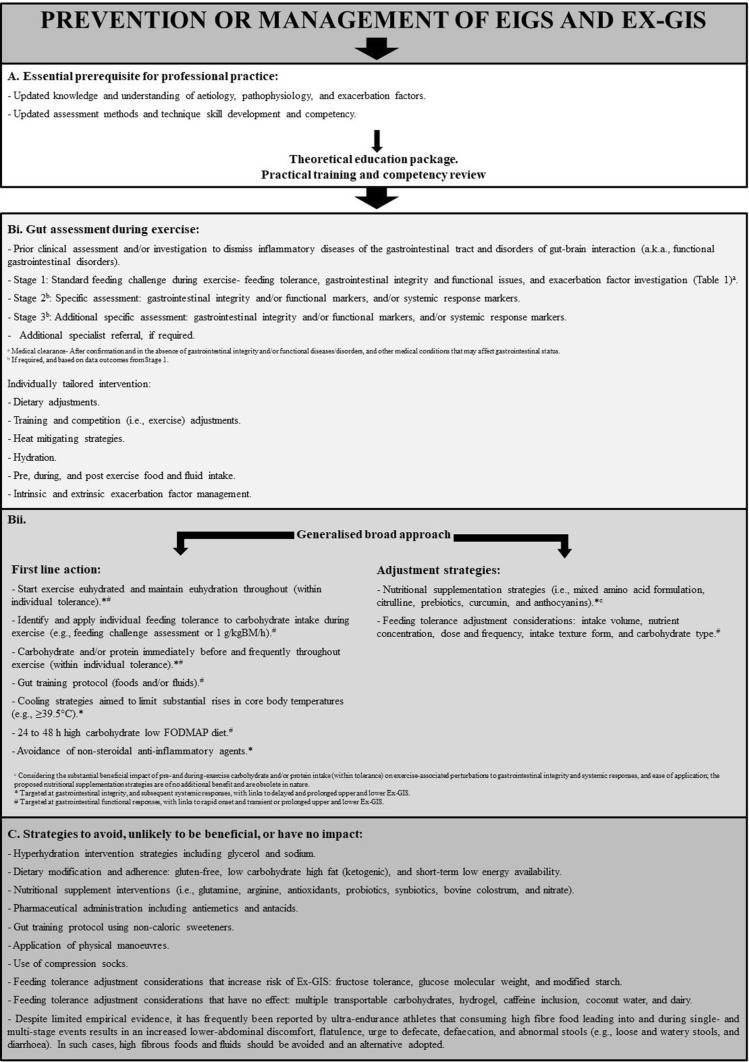
Schematic flow illustration of the professional practice guidance for the prevention or management of exercise-induced gastrointestinal syndrome (EIGS) and aligned exercise-associated gastrointestinal symptoms (Ex-GIS) [12, 67, 75]

From historical to more recent evidence, it is now established that exercise stress causes an array of asymptomatic and symptomatic gastrointestinal integrity and functional disturbances, which may lead to health-impacting systemic responses and outcomes. Furthermore, such gastrointestinal disturbances are linked with rapid onset or delayed, and acute or prolonged, gastrointestinal symptoms, which have profound performance-impairment implications (i.e., reduced workload, cessation of exercise, or withdrawal from activity). From a professional practice perspective, it is therefore not surprising that athletes and athlete support practitioners are exploring effective ways to prevent and manage exercise-associated gastrointestinal perturbations and aligned symptoms. On the basis of the current available evidence from exercise gastroenterology research that generally meets a minimum level of quality of methodologies for best-practice experimental design, Table 3 presents a summary of prevention and management strategies and their impact on parameters of EIGS and Ex-GIS. On the basis of the efficacy of presented strategies, Fig. 2 presents a professional practice guidance schematic in the prevention and management of EIGS and Ex-GIS.

In short, the first-line actions consist of: (i) preparing the gastrointestinal tract for exercise; (ii) limiting gastrointestinal integrity, functional perturbation, and systemic responses during exercise; and (iii) restoring gastrointestinal patency post-exercise to support recovery nutrition. First-line actions also include avoiding factors and strategies reported to exacerbate or have no impact on EIGS and Ex-GIS. For those athletes experiencing recurrent Ex-GIS episodes, second-line action include undertaking a gastrointestinal assessment during exercise, as described in supplementary file 3 entitled Translation to practice: Assessment and intervention procedures for clinical practice—Supporting the individual athlete. This process would take into consideration individualized data and findings from testing procedures into EIGS and Ex-GIS causal factors and align with relevant prevention and management strategies that have proven effective in research settings. Adopting and adhering to such assessment and intervention procedures requires a multidisciplinary approach. Therefore, understanding one’s scope of practice and appropriate local and/or external referral pathways is a fundamental part of competent and effective practice. In addition, it is recommended that professional practitioners supporting athletes with gastrointestinal issues undertake ongoing education and training to gain or update knowledge and understanding of how the gastrointestinal tract responds to exercise, thus enabling the provisions of effective and competent practice.

## Supplementary Information

Below is the link to the electronic supplementary material.

Supplementary file1 (PDF 236 KB)

Supplementary file1 (PDF 320 KB)

Supplementary file2 (PDF 393 KB)

## Declarations

### Availability of Data and Materials

Data and materials presented within this review can be made available by contacting the corresponding author, at reasonable request.

## References

[1] Gordon B, Levine SA, Wilmaers A. Observations on a group of marathon runners: with special reference to the circulation. Arch Intern Med. 1924;33(4):425–34. 10.1001/archinte.1924.00110280023003.

[2] www.history.com/topics/sports/olympic-games.

[3] Costa RJS, Snipe RMJ, Kitic CM, Gibson PR. Systematic review: exercise-induced gastrointestinal syndrome-implications for health and intestinal disease. Aliment Pharmacol Ther. 2017;46(3):246–65. 10.1111/apt.14157. (**Epub 20170607**). 28589631 10.1111/apt.14157

[4] Brouns F, Saris WH, Rehrer NJ. Abdominal complaints and gastrointestinal function during long-lasting exercise. Int J Sports Med. 1987;8(3):175–89. 10.1055/s-2008-1025653. 3305389 10.1055/s-2008-1025653

[5] Rehrer NJ, Beckers E, Brouns F, ten Hoor F, Saris WH. Exercise and training effects on gastric emptying of carbohydrate beverages. Med Sci Sports Exerc. 1989;21(5):540–9. 2691815

[6] Rehrer NJ, van Kemenade M, Meester W, Brouns F, Saris WH. Gastrointestinal complaints in relation to dietary intake in triathletes. Int J Sport Nutr. 1992;2(1):48–59. 10.1123/ijsn.2.1.48. 1338583 10.1123/ijsn.2.1.48

[7] Rehrer NJ, Meijer GA. Biomechanical vibration of the abdominal region during running and bicycling. J Sports Med Phys Fitness. 1991;31(2):231–4. 1753730

[8] Worobetz LJ, Gerrard DF. Gastrointestinal symptoms during exercise in Enduro athletes: prevalence and speculations on the aetiology. N Z Med J. 1985;98(784):644–6. 3861978

[9] Scrivin R, Costa RJ, Pelly F, Lis D, Slater G. Development and validation of a questionnaire investigating endurance athletes practices to manage gastrointestinal symptoms around exercise. Nutr Diet. 2021;78(3):286–95. 10.1111/1747-0080.12674. (**Epub 20210527**). 34047004 10.1111/1747-0080.12674

[10] Scrivin R, Costa RJS, Pelly F, Lis D, Slater G. An exploratory study of the management strategies reported by endurance athletes with exercise-associated gastrointestinal symptoms. Front Nutr. 2022;9:1003445. 10.3389/fnut.2022.1003445. (**Epub 20221109**). 36438762 10.3389/fnut.2022.1003445 PMC9691682

[11] Gaskell SK, Costa RJS. Applying a low-FODMAP dietary intervention to a female ultraendurance runner with irritable bowel syndrome during a multistage ultramarathon. Int J Sport Nutr Exerc Metab. 2019;29(1):61–7. 10.1123/ijsnem.2017-0398. (**Epub 20180926**). 29757053 10.1123/ijsnem.2017-0398

[12] Gaskell SK, Rauch CE, Costa RJS. Gastrointestinal assessment and therapeutic intervention for the management of exercise-associated gastrointestinal symptoms: a case series translational and professional practice approach. Front Physiol. 2021;12: 719142. 10.3389/fphys.2021.719142. (**Epub 20210907**). 34557109 10.3389/fphys.2021.719142 PMC8452991

[13] Lis D, Ahuja KD, Stellingwerff T, Kitic CM, Fell J. Case study: utilizing a low FODMAP diet to combat exercise-induced gastrointestinal symptoms. Int J Sport Nutr Exerc Metab. 2016;26(5):481–7. 10.1123/ijsnem.2015-0293. (**Epub 20160824**). 27097380 10.1123/ijsnem.2015-0293

[14] Mujika I. Case Study: Long-term low-carbohydrate, high-fat diet impairs performance and subjective well-being in a world-class vegetarian long-distance triathlete. Int J Sport Nutr Exerc Metab. 2019;29(3):339–44. 10.1123/ijsnem.2018-0124. (**Epub 20181113**). 30160554 10.1123/ijsnem.2018-0124

[15] Costa RJ, Gill S, Hankey J, Scheer V, Murray A, Thake D. Compromised energy and nutritional intake of ultra-endurance runners during a multi-stage ultra-marathon conducted in a hot ambient environment. Int J Sports Sci. 2013;3(2):51–61. 10.5923/j.sports.20130302.03.

[16] Costa RJ, Gill SK, Hankey J, Wright A, Marczak S. Perturbed energy balance and hydration status in ultra-endurance runners during a 24 h ultra-marathon. Br J Nutr. 2014;112(3):428–37. 10.1017/s0007114514000907. (**Epub 20140513**). 24818799 10.1017/S0007114514000907

[17] Costa RJ, Snipe R, Camões-Costa V, Scheer V, Murray A. The impact of gastrointestinal symptoms and dermatological injuries on nutritional intake and hydration status during ultramarathon events. Sports Med Open. 2016;2:16. 10.1186/s40798-015-0041-9. (**Epub 20160105**). 26767151 10.1186/s40798-015-0041-9 PMC4701764

[18] Engebretsen L, Soligard T, Steffen K, Alonso JM, Aubry M, Budgett R, et al. Sports injuries and illnesses during the London Summer Olympic Games 2012. Br J Sports Med. 2013;47(7):407–14. 10.1136/bjsports-2013-092380. (**Epub 20130320**). 23515712 10.1136/bjsports-2013-092380

[19] Jeukendrup AE, Vet-Joop K, Sturk A, Stegen JH, Senden J, Saris WH, et al. Relationship between gastro-intestinal complaints and endotoxaemia, cytokine release and the acute-phase reaction during and after a long-distance triathlon in highly trained men. Clin Sci (Lond). 2000;98(1):47–55. 10600658

[20] Pfeiffer B, Stellingwerff T, Hodgson AB, Randell R, Pöttgen K, Res P, et al. Nutritional intake and gastrointestinal problems during competitive endurance events. Med Sci Sports Exerc. 2012;44(2):344–51. 10.1249/MSS.0b013e31822dc809. 21775906 10.1249/MSS.0b013e31822dc809

[21] Benmassaoud A, Kanber Y, Nawar J, Bessissow T. Exercise-induced ischemic colitis in an amateur marathon runner. Endoscopy. 2014;46(Suppl 1 UCTN):E480. 10.1055/s-0034-1377536. (**Epub 20141014**). 25314207 10.1055/s-0034-1377536

[22] Cohen DC, Winstanley A, Engledow A, Windsor AC, Skipworth JR. Marathon-induced ischemic colitis: why running is not always good for you. Am J Emerg Med. 2009;27(2):255.e5-7. 10.1016/j.ajem.2008.06.033. 10.1016/j.ajem.2008.06.033 19371557

[23] García Gavilán MC, Morales Alcázar F, Montes Aragón C, Sánchez Cantos AM. Ischemic colitis of the right colon after triathlon: the importance of high clinical suspicion. Gastroenterol Hepatol. 2021;44(8):565–6. 10.1016/j.gastrohep.2020.08.010. (**Epub 20201112**). 33189408 10.1016/j.gastrohep.2020.08.010

[24] Grames C, Berry-Cabán CS. Ischemic colitis in an endurance runner. Case Rep Gastrointest Med. 2012;2012: 356895. 10.1155/2012/356895. (**Epub 20121010**). 23091744 10.1155/2012/356895 PMC3474219

[25] Robertson JD, Maughan RJ, Davidson RJ. Faecal blood loss in response to exercise. Br Med J (Clin Res Ed). 1987;295(6593):303–5. 10.1136/bmj.295.6593.303. 10.1136/bmj.295.6593.303 PMC1247142 3115419

[26] Gaskell SK, Rauch CE, Parr A, Costa RJS. Diurnal versus nocturnal exercise-effect on the gastrointestinal tract. Med Sci Sports Exerc. 2021;53(5):1056–67. 10.1249/mss.0000000000002546. 33065594 10.1249/MSS.0000000000002546

[27] Gaskell SK, Burgell R, Wiklendt L, Dinning P, Costa RJS. Does exertional heat stress impact gastrointestinal function and symptoms? J Sci Med Sport. 2022;25(12):960–7. 10.1016/j.jsams.2022.10.008. (**Epub 20221017**). 36347748 10.1016/j.jsams.2022.10.008

[28] Gaskell SK, Burgell R, Wiklendt L, Dinning PG, Costa RJS. Impact of exercise duration on gastrointestinal function and symptoms. J Appl Physiol (1985). 2023;134(1):160–71. 10.1152/japplphysiol.00393.2022. (**Epub 20221208**). 36476157 10.1152/japplphysiol.00393.2022

[29] Gaskell SK, Henningsen K, Young P, Gill P, Muir J, Henry R, et al. The impact of a 24-h low and high fermentable oligo- di- mono-saccharides and polyol (FODMAP) diet on plasma bacterial profile in response to exertional-heat stress. Nutrients. 2023. 10.3390/nu15153376. (**Epub 20230729**). 10.3390/nu15153376 PMC10420669 37571312

[30] Gill SK, Hankey J, Wright A, Marczak S, Hemming K, Allerton DM, et al. The impact of a 24-h ultra-marathon on circulatory endotoxin and cytokine profile. Int J Sports Med. 2015;36(8):688–95. 10.1055/s-0034-1398535. (**Epub 20150505**). 25941924 10.1055/s-0034-1398535

[31] Gill SK, Teixeira A, Rama L, Prestes J, Rosado F, Hankey J, et al. Circulatory endotoxin concentration and cytokine profile in response to exertional-heat stress during a multi-stage ultra-marathon competition. Exerc Immunol Rev. 2015;21:114–28. 25830597

[32] Laitano O, Leon LR, Roberts WO, Sawka MN. Controversies in exertional heat stroke diagnosis, prevention, and treatment. J Appl Physiol (1985). 2019;127(5):1338–48. 10.1152/japplphysiol.00452.2019. (**Epub 20190923**). 31545156 10.1152/japplphysiol.00452.2019

[33] Young P, Russo I, Gill P, Muir J, Henry R, Davidson Z, et al. Reliability of pathophysiological markers reflective of exercise-induced gastrointestinal syndrome (EIGS) in response to 2-h high-intensity interval exercise: a comprehensive methodological efficacy exploration. Front Physiol. 2023;14:1063335. 10.3389/fphys.2023.1063335. (**Epub 20230221**). 36895638 10.3389/fphys.2023.1063335 PMC9989174

[34] Gaskell SK, Lis DM, Costa RJS. Exercise-induced gastrointestinal syndrome. In: Burke LDV, Minehan M, editors. Clinical sports nutrition. 6th ed. Sydney: McGraw-Hill Education; 2021. p. 551–75.

[35] Perko MJ, Nielsen HB, Skak C, Clemmesen JO, Schroeder TV, Secher NH. Mesenteric, celiac and splanchnic blood flow in humans during exercise. J Physiol. 1998;513(Pt 3):907–13. 10.1111/j.1469-7793.1998.907ba.x. 9824727 10.1111/j.1469-7793.1998.907ba.x PMC2231328

[36] Rehrer NJ, Smets A, Reynaert H, Goes E, De Meirleir K. Effect of exercise on portal vein blood flow in man. Med Sci Sports Exerc. 2001;33(9):1533–7. 10.1097/00005768-200109000-00017. 11528343 10.1097/00005768-200109000-00017

[37] Grootjans J, Lenaerts K, Buurman WA, Dejong CH, Derikx JP. Life and death at the mucosal-luminal interface: new perspectives on human intestinal ischemia-reperfusion. World J Gastroenterol. 2016;22(9):2760–70. 10.3748/wjg.v22.i9.2760. 26973414 10.3748/wjg.v22.i9.2760 PMC4777998

[38] Henningsen K, Martinez I, Costa RJS. Exertional stress-induced pathogenic luminal content translocation—Friend or foe? Int J Sports Med. 2024. 10.1055/a-2235-1629. (**Epub 20240129**). 10.1055/a-2235-1629 38286406

[39] van Wijck K, Lenaerts K, van Loon LJ, Peters WH, Buurman WA, Dejong CH. Exercise-induced splanchnic hypoperfusion results in gut dysfunction in healthy men. PLoS ONE. 2011;6(7): e22366. 10.1371/journal.pone.0022366. (**Epub 20110721**). 21811592 10.1371/journal.pone.0022366 PMC3141050

[40] Young P, Rauch C, Russo I, Gaskell S, Davidson Z, Costa RJS. Plasma endogenous endotoxin core antibody response to exercise in endurance athletes. Int J Sports Med. 2022;43(12):1023–32. 10.1055/a-1827-3124. (**Epub 20220414**). 35426092 10.1055/a-1827-3124 PMC9622302

[41] Costa RJS, Camões-Costa V, Snipe RMJ, Dixon D, Russo I, Huschtscha Z. Impact of exercise-induced hypohydration on gastrointestinal integrity, function, symptoms, and systemic endotoxin and inflammatory profile. J Appl Physiol (1985). 2019;126(5):1281–91. 10.1152/japplphysiol.01032.2018. (**Epub 20190321**). 30896356 10.1152/japplphysiol.01032.2018

[42] Costa RJS, Miall A, Khoo A, Rauch C, Snipe R, Camões-Costa V, et al. Gut-training: the impact of two weeks repetitive gut-challenge during exercise on gastrointestinal status, glucose availability, fuel kinetics, and running performance. Appl Physiol Nutr Metab. 2017;42(5):547–57. 10.1139/apnm-2016-0453. (**Epub 20170322**). 28177715 10.1139/apnm-2016-0453

[43] Miall A, Khoo A, Rauch C, Snipe RMJ, Camões-Costa VL, Gibson PR, et al. Two weeks of repetitive gut-challenge reduce exercise-associated gastrointestinal symptoms and malabsorption. Scand J Med Sci Sports. 2018;28(2):630–40. 10.1111/sms.12912. (**Epub 20170619**). 28508559 10.1111/sms.12912

[44] Russo I, Della Gatta PA, Garnham A, Porter J, Burke LM, Costa RJS. Does the nutritional composition of dairy milk based recovery beverages influence post-exercise gastrointestinal and immune status, and subsequent markers of recovery optimisation in response to high intensity interval exercise? Front Nutr. 2020;7: 622270. 10.3389/fnut.2020.622270. (**Epub 20210114**). 33521041 10.3389/fnut.2020.622270 PMC7840831

[45] Russo I, Della Gatta PA, Garnham A, Porter J, Burke LM, Costa RJS. Assessing overall exercise recovery processes using carbohydrate and carbohydrate-protein containing recovery beverages. Front Physiol. 2021;12: 628863. 10.3389/fphys.2021.628863. (**Epub 20210204**). 33613323 10.3389/fphys.2021.628863 PMC7890126

[46] Russo I, Della Gatta PA, Garnham A, Porter J, Burke LM, Costa RJS. The effects of an acute “train-low” nutritional protocol on markers of recovery optimization in endurance-trained male athletes. Int J Sports Physiol Perform. 2021;16(12):1764–76. 10.1123/ijspp.2020-0847. (**Epub 20210527**). 34044369 10.1123/ijspp.2020-0847

[47] Fukudo S. Role of corticotropin-releasing hormone in irritable bowel syndrome and intestinal inflammation. J Gastroenterol. 2007;42(Suppl 17):48–51. 10.1007/s00535-006-1942-7. 17238026 10.1007/s00535-006-1942-7

[48] Hirst GD, Ward SM. Interstitial cells: involvement in rhythmicity and neural control of gut smooth muscle. J Physiol. 2003;550(Pt 2):337–46. 10.1113/jphysiol.2003.043299. (**Epub 20030606**). 12794179 10.1113/jphysiol.2003.043299 PMC2343044

[49] Holzer P, Farzi A, Hassan AM, Zenz G, Jačan A, Reichmann F. visceral inflammation and immune activation stress the brain. Front Immunol. 2017;8:1613. 10.3389/fimmu.2017.01613. (**Epub 20171122**). 29213271 10.3389/fimmu.2017.01613 PMC5702648

[50] Lin YM, Li F, Shi XZ. Mechanical stress is a pro-inflammatory stimulus in the gut: in vitro, in vivo and ex vivo evidence. PLoS ONE. 2014;9(9): e106242. 10.1371/journal.pone.0106242. (**Epub 20140902**). 25180799 10.1371/journal.pone.0106242 PMC4152012

[51] Merrells RJ, Cripps AJ, Chivers PT, Fournier PA. Role of lactic acidosis as a mediator of sprint-mediated nausea. Physiol Rep. 2019;7(21): e14283. 10.14814/phy2.14283. 31724342 10.14814/phy2.14283 PMC6854110

[52] Merrells RJ, Madon SB, Chivers PT, Fournier PA. Nausea after repeated sprints: is lactic acidosis really the culprit? Med Sci Sports Exerc. 2021;53(9):1865–72. 10.1249/mss.0000000000002667. 34398059 10.1249/MSS.0000000000002667

[53] Smith TJ, Wilson MA, Karl JP, Austin K, Bukhari A, Pasiakos SM, et al. Interstitial glucose concentrations and hypoglycemia during 2 days of caloric deficit and sustained exercise: a double-blind, placebo-controlled trial. J Appl Physiol (1985). 2016;121(5):1208–16. 10.1152/japplphysiol.00432.2016. (**Epub 20160929**). 27687559 10.1152/japplphysiol.00432.2016

[54] Sharkey KA, Savidge TC. Role of enteric neurotransmission in host defense and protection of the gastrointestinal tract. Auton Neurosci. 2014;181:94–106. 10.1016/j.autneu.2013.12.006. (**Epub 20131222**). 24412639 10.1016/j.autneu.2013.12.006 PMC3944077

[55] Shin HS, Ingram JR, McGill AT, Poppitt SD. Lipids, CHOs, proteins: can all macronutrients put a “brake” on eating? Physiol Behav. 2013;120:114–23. 10.1016/j.physbeh.2013.07.008. (**Epub 20130801**). 23911804 10.1016/j.physbeh.2013.07.008

[56] Layer P, Peschel S, Schlesinger T, Goebell H. Human pancreatic secretion and intestinal motility: effects of ileal nutrient perfusion. Am J Physiol. 1990;258(2 Pt 1):G196-201. 10.1152/ajpgi.1990.258.2.G196. 1689548 10.1152/ajpgi.1990.258.2.G196

[57] van Avesaat M, Troost FJ, Ripken D, Hendriks HF, Masclee AA. Ileal brake activation: macronutrient-specific effects on eating behavior? Int J Obes (Lond). 2015;39(2):235–43. 10.1038/ijo.2014.112. (**Epub 20140624**). 24957485 10.1038/ijo.2014.112

[58] Van Citters GW, Lin HC. Ileal brake: neuropeptidergic control of intestinal transit. Curr Gastroenterol Rep. 2006;8(5):367–73. 10.1007/s11894-006-0021-9. 16968603 10.1007/s11894-006-0021-9

[59] Fleshner M, Crane CR. Exosomes, DAMPs and miRNA: features of stress physiology and immune homeostasis. Trends Immunol. 2017;38(10):768–76. 10.1016/j.it.2017.08.002. (**Epub 20170823**). 28838855 10.1016/j.it.2017.08.002 PMC5624844

[60] Moses FM. The effect of exercise on the gastrointestinal tract. Sports Med. 1990;9(3):159–72. 10.2165/00007256-199009030-00004. 2180030 10.2165/00007256-199009030-00004

[61] ter Steege RW, Van der Palen J, Kolkman JJ. Prevalence of gastrointestinal complaints in runners competing in a long-distance run: an internet-based observational study in 1281 subjects. Scand J Gastroenterol. 2008;43(12):1477–82. 10.1080/00365520802321170. 18777440 10.1080/00365520802321170

[62] ter Steege RW, Kolkman JJ. Review article: the pathophysiology and management of gastrointestinal symptoms during physical exercise, and the role of splanchnic blood flow. Aliment Pharmacol Ther. 2012;35(5):516–28. 10.1111/j.1365-2036.2011.04980.x. (**Epub 20120110**). 22229513 10.1111/j.1365-2036.2011.04980.x

[63] Stuempfle KJ, Hoffman MD. Gastrointestinal distress is common during a 161-km ultramarathon. J Sports Sci. 2015;33(17):1814–21. 10.1080/02640414.2015.1012104. (**Epub 20150226**). 25716739 10.1080/02640414.2015.1012104

[64] Alcock R, McCubbin A, Camões-Costa V, Costa RJS. Case Study: Providing nutritional support to an ultraendurance runner in preparation for a self-sufficient multistage ultramarathon: rationed versus full energy provisions. Wilderness Environ Med. 2018;29(4):508–20. 10.1016/j.wem.2018.06.004. (**Epub 20180922**). 30249353 10.1016/j.wem.2018.06.004

[65] King AJ, Etxebarria N, Ross ML, Garvican-Lewis L, Heikura IA, McKay AKA, et al. Short-term very high carbohydrate diet and gut-training have minor effects on gastrointestinal status and performance in highly trained endurance athletes. Nutrients. 2022. 10.3390/nu14091929. (**Epub 20220505**). 10.3390/nu14091929 PMC9105618 35565896

[66] Rauch CE, McCubbin AJ, Gaskell SK, Costa RJS. Feeding tolerance, glucose availability, and whole-body total carbohydrate and fat oxidation in male endurance and ultra-endurance runners in response to prolonged exercise, consuming a habitual mixed macronutrient diet and carbohydrate feeding during exercise. Front Physiol. 2021;12: 773054. 10.3389/fphys.2021.773054. (**Epub 20220104**). 35058795 10.3389/fphys.2021.773054 PMC8764139

[67] Costa RJS, Young P, Gill SK, Snipe RMJ, Gaskell S, Russo I, et al. assessment of exercise-associated gastrointestinal perturbations in research and practical settings: methodological concerns and recommendations for best practice. Int J Sport Nutr Exerc Metab. 2022;32(5):387–418. 10.1123/ijsnem.2022-0048. (**Epub 20220813**). 35963615 10.1123/ijsnem.2022-0048

[68] Gaskell SK, Snipe RMJ, Costa RJS. Test–retest reliability of a modified visual analog scale assessment tool for determining incidence and severity of gastrointestinal symptoms in response to exercise stress. Int J Sport Nutr Exerc Metab. 2019;29(4):411–9. 10.1123/ijsnem.2018-0215. (**Epub 20190701**). 30632417 10.1123/ijsnem.2018-0215

[69] Drossman DA. The functional gastrointestinal disorders and the Rome III process. Gastroenterology. 2006;130(5):1377–90. 10.1053/j.gastro.2006.03.008. 16678553 10.1053/j.gastro.2006.03.008

[70] Drossman DA. Functional gastrointestinal disorders: history, pathophysiology, clinical features and Rome IV. Gastroenterology. 2016. 10.1053/j.gastro.2016.02.032. (**Epub 20160219**). 10.1053/j.gastro.2016.02.032 27144617

[71] Snipe RMJ, Khoo A, Kitic CM, Gibson PR, Costa RJS. The impact of exertional-heat stress on gastrointestinal integrity, gastrointestinal symptoms, systemic endotoxin and cytokine profile. Eur J Appl Physiol. 2018;118(2):389–400. 10.1007/s00421-017-3781-z. (**Epub 20171212**). 29234915 10.1007/s00421-017-3781-z

[72] Snipe RMJ, Khoo A, Kitic CM, Gibson PR, Costa RJS. The impact of mild heat stress during prolonged running on gastrointestinal integrity, gastrointestinal symptoms, systemic endotoxin and cytokine profiles. Int J Sports Med. 2018. 10.1055/s-0043-122742. (**Epub 20180207**). 10.1055/s-0043-122742 29415294

[73] Snipe RMJ, Khoo A, Kitic CM, Gibson PR, Costa RJS. Carbohydrate and protein intake during exertional heat stress ameliorates intestinal epithelial injury and small intestine permeability. Appl Physiol Nutr Metab. 2017;42(12):1283–92. 10.1139/apnm-2017-0361. (**Epub 20170804**). 28777927 10.1139/apnm-2017-0361

[74] Keeffe EB, Lowe DK, Goss JR, Wayne R. Gastrointestinal symptoms of marathon runners. West J Med. 1984;141(4):481–4. 6506684 PMC1021858

[75] Gaskell SKRC, Costa RJS. Gastrointestinal assessment and management procedures for exercise-associated gastrointestinal symptoms. Aspetar Sports Med J. 2021;10:36–44.

[76] Walter E, Gibson OR, Stacey M, Hill N, Parsons IT, Woods D. Changes in gastrointestinal cell integrity after marathon running and exercise-associated collapse. Eur J Appl Physiol. 2021;121(4):1179–87. 10.1007/s00421-021-04603-w. (**Epub 20210129**). 33512586 10.1007/s00421-021-04603-w

[77] Hart TL, Townsend JR, Grady NJ, Johnson KD, Littlefield LA, Vergne MJ, et al. Resistance exercise increases gastrointestinal symptoms, markers of gut permeability, and damage in resistance-trained adults. Med Sci Sports Exerc. 2022;54(10):1761–70. 10.1249/mss.0000000000002967. (**Epub 20220525**). 35612399 10.1249/MSS.0000000000002967

[78] Horner KM, Schubert MM, Desbrow B, Byrne NM, King NA. Acute exercise and gastric emptying: a meta-analysis and implications for appetite control. Sports Med. 2015;45(5):659–78. 10.1007/s40279-014-0285-4. 25398225 10.1007/s40279-014-0285-4

[79] Pals KL, Chang RT, Ryan AJ, Gisolfi CV. Effect of running intensity on intestinal permeability. J Appl Physiol (1985). 1997;82(2):571–6. 10.1152/jappl.1997.82.2.571. 9049739 10.1152/jappl.1997.82.2.571

[80] van Wijck K, Pennings B, van Bijnen AA, Senden JM, Buurman WA, Dejong CH, et al. Dietary protein digestion and absorption are impaired during acute postexercise recovery in young men. Am J Physiol Regul Integr Comp Physiol. 2013;304(5):R356–61. 10.1152/ajpregu.00294.2012. (**Epub 20130102**). 23283940 10.1152/ajpregu.00294.2012

[81] Costa RJS, Mika AS, McCubbin AJ. The impact of exercise modality on exercise-induced gastrointestinal syndrome and associated gastrointestinal symptoms. J Sci Med Sport. 2022;25(10):788–93. 10.1016/j.jsams.2022.07.003. (**Epub 20220712**). 35868987 10.1016/j.jsams.2022.07.003

[82] Gaskell SK, Taylor B, Muir J, Costa RJS. Impact of 24-h high and low fermentable oligo-, di-, monosaccharide, and polyol diets on markers of exercise-induced gastrointestinal syndrome in response to exertional heat stress. Appl Physiol Nutr Metab. 2020;45(6):569–80. 10.1139/apnm-2019-0187. (**Epub 20191025**). 31652404 10.1139/apnm-2019-0187

[83] Henningsen K, Mika A, Alcock R, Gaskell SK, Parr A, Rauch C, et al. The increase in core body temperature in response to exertional-heat stress can predict exercise-induced gastrointestinal syndrome. Temperature. 2023. 10.1080/23328940.2023.2213625. 10.1080/23328940.2023.2213625 PMC10989703 38577295

[84] Arribalzaga S, Viribay A, Calleja-González J, Fernández-Lázaro D, Castañeda-Babarro A, Mielgo-Ayuso J. Relationship of carbohydrate intake during a single-stage one-day ultra-trail race with fatigue outcomes and gastrointestinal problems: a systematic review. Int J Environ Res Public Health. 2021. 10.3390/ijerph18115737. (**Epub 20210527**). 10.3390/ijerph18115737 PMC8197833 34071815

[85] McKenna ZJ, Fennel ZJ, Berkemeier QN, Nava RC, Amorim FT, Deyhle MR, et al. Exercise in hypobaric hypoxia increases markers of intestinal injury and symptoms of gastrointestinal distress. Exp Physiol. 2022;107(4):326–36. 10.1113/ep090266. (**Epub 20220309**). 35224797 10.1113/EP090266

[86] McKenna ZJ, Gorini Pereira F, Gillum TL, Amorim FT, Deyhle MR, Mermier CM. High-altitude exposures and intestinal barrier dysfunction. Am J Physiol Regul Integr Comp Physiol. 2022;322(3):R192-r203. 10.1152/ajpregu.00270.2021. (**Epub 20220119**). 35043679 10.1152/ajpregu.00270.2021

[87] Iwamoto J, Saito Y, Honda A, Matsuzaki Y. Clinical features of gastroduodenal injury associated with long-term low-dose aspirin therapy. World J Gastroenterol. 2013;19(11):1673–82. 10.3748/wjg.v19.i11.1673. 23555156 10.3748/wjg.v19.i11.1673 PMC3607744

[88] Lambert GP, Broussard LJ, Mason BL, Mauermann WJ, Gisolfi CV. Gastrointestinal permeability during exercise: effects of aspirin and energy-containing beverages. J Appl Physiol (1985). 2001;90(6):2075–80. 10.1152/jappl.2001.90.6.2075. 11356768 10.1152/jappl.2001.90.6.2075

[89] Lambert GP, Boylan M, Laventure JP, Bull A, Lanspa S. Effect of aspirin and ibuprofen on GI permeability during exercise. Int J Sports Med. 2007;28(9):722–6. 10.1055/s-2007-964891. (**Epub 20070413**). 17436199 10.1055/s-2007-964891

[90] Ryan AJ, Chang RT, Gisolfi CV. Gastrointestinal permeability following aspirin intake and prolonged running. Med Sci Sports Exerc. 1996;28(6):698–705. 10.1097/00005768-199606000-00009. 8784758 10.1097/00005768-199606000-00009

[91] Van Wijck K, Lenaerts K, Van Bijnen AA, Boonen B, Van Loon LJ, Dejong CH, et al. Aggravation of exercise-induced intestinal injury by ibuprofen in athletes. Med Sci Sports Exerc. 2012;44(12):2257–62. 10.1249/MSS.0b013e318265dd3d. 22776871 10.1249/MSS.0b013e318265dd3d

[92] Warden SJ. Prophylactic use of NSAIDs by athletes: a risk/benefit assessment. Phys Sportsmed. 2010;38(1):132–8. 10.3810/psm.2010.04.1770. 20424410 10.3810/psm.2010.04.1770

[93] Bennett CJ, Henry R, Snipe RMJ, Costa RJS. Is the gut microbiota bacterial abundance and composition associated with intestinal epithelial injury, systemic inflammatory profile, and gastrointestinal symptoms in response to exertional-heat stress? J Sci Med Sport. 2020;23(12):1141–53. 10.1016/j.jsams.2020.06.002. (**Epub 20200623**). 32620352 10.1016/j.jsams.2020.06.002

[94] Russo I, Della Gatta PA, Garnham A, Porter J, Burke LM, Costa RJS. The influence of fitness status and biological sex on markers of recovery optimisation in response to prolonged high intensity interval exercise. Int J Sports Sci. 2020;10(6):145–63.

[95] Snipe RMJ, Costa RJS. Does biological sex impact intestinal epithelial injury, small intestine permeability, gastrointestinal symptoms and systemic cytokine profile in response to exertional-heat stress? J Sports Sci. 2018;36(24):2827–35. 10.1080/02640414.2018.1478612. (**Epub 20180523**). 29790452 10.1080/02640414.2018.1478612

[96] Scheer V, Costa RJS, Doutreleau S, Knechtle B, Nikolaidis PT, Roberts WO, et al. Recommendations on youth participation in ultra-endurance running events: a consensus statement. Sports Med. 2021;51(6):1123–35. 10.1007/s40279-021-01441-w. (**Epub 20210311**). 33704697 10.1007/s40279-021-01441-w

[97] Young P, Davidson Z, Scheer V, Costa RJS, editor. The impact of prolonged endurance exercise on markers of exercise-induced gastrointestinal syndrome in youth versus adult endurance athletes. Sports Dietitians Australia; 2023: Nutrition and Dietetics.

[98] Young P, Henningsen K, Snipe R, Gaskell S, Alcock R, Mika A, et al. Does age influence gastrointestinal status responses to exertional-heat stress? Int J Sports Med. 2024. 10.1055/a-2195-3131. (**Epub 20240110**). 10.1055/a-2195-3131 38198808

[99] Selkirk GA, McLellan TM, Wright HE, Rhind SG. Mild endotoxemia, NF-kappaB translocation, and cytokine increase during exertional heat stress in trained and untrained individuals. Am J Physiol Regul Integr Comp Physiol. 2008;295(2):R611–23. 10.1152/ajpregu.00917.2007. 18565834 10.1152/ajpregu.00917.2007

[100] Guillochon M, Rowlands DS. Solid, gel, and liquid carbohydrate format effects on gut comfort and performance. Int J Sport Nutr Exerc Metab. 2017;27(3):247–54. 10.1123/ijsnem.2016-0211. (**Epub 20161220**). 27997257 10.1123/ijsnem.2016-0211

[101] Hoffman MD, Snipe RMJ, Costa RJS. Ad libitum drinking adequately supports hydration during 2 h of running in different ambient temperatures. Eur J Appl Physiol. 2018;118(12):2687–97. 10.1007/s00421-018-3996-7. (**Epub 20180928**). 30267225 10.1007/s00421-018-3996-7

[102] Marcora SM, Staiano W, Manning V. Mental fatigue impairs physical performance in humans. J Appl Physiol (1985). 2009;106(3):857–64. 10.1152/japplphysiol.91324.2008. (**Epub 20090108**). 19131473 10.1152/japplphysiol.91324.2008

[103] Wilson PB. Perceived life stress and anxiety correlate with chronic gastrointestinal symptoms in runners. J Sports Sci. 2018;36(15):1713–9. 10.1080/02640414.2017.1411175. (**Epub 20171201**). 29192838 10.1080/02640414.2017.1411175

[104] Wilson PB, Russell H, Pugh J. Anxiety may be a risk factor for experiencing gastrointestinal symptoms during endurance races: an observational study. Eur J Sport Sci. 2021;21(3):421–7. 10.1080/17461391.2020.1746836. (**Epub 20200406**). 32251613 10.1080/17461391.2020.1746836

[105] Wynne JL, Wilson PB. Thorn in your side or thorn in your head? anxiety and stress as correlates of exercise-related transient abdominal pain. Clin J Sport Med. 2022;32(5):471–5. 10.1097/jsm.0000000000001000. (**Epub 20211207**). 36083326 10.1097/JSM.0000000000001000

[106] Martinez IG, Biesiekierski JR, Rauch CE, Costa RJS. Repetitive feeding-challenge with different nutritional densities on markers of gastrointestinal function, substrate oxidation and endurance exercise performance. Int J Sport Nutr Exerc Metab. 2025. 10.1123/ijsnem.2024-0145. 10.1123/ijsnem.2024-0145 39914376

[107] Thomas DT, Erdman KA, Burke LM. American College of Sports Medicine Joint Position Statement. Nutrition and athletic performance. Med Sci Sports Exerc. 2016;48(3):543–68. 10.1249/mss.0000000000000852. 26891166 10.1249/MSS.0000000000000852

[108] Gupta R, Yin L, Grosche A, Lin S, Xu X, Guo J, et al. An amino acid-based oral rehydration solution regulates radiation-induced intestinal barrier disruption in mice. J Nutr. 2020;150(5):1100–8. 10.1093/jn/nxaa025. 32133527 10.1093/jn/nxaa025

[109] Matheson PJ, Wilson MA, Garrison RN. Regulation of intestinal blood flow. J Surg Res. 2000;93(1):182–96. 10.1006/jsre.2000.5862. 10945962 10.1006/jsre.2000.5862

[110] Xiao H, Zha C, Shao F, Wang L, Tan B. Amino acids regulate energy utilization through mammalian target of rapamycin complex 1 and adenosine monophosphate activated protein kinase pathway in porcine enterocytes. Anim Nutr. 2020;6(1):98–106. 10.1016/j.aninu.2019.12.001. (**Epub 20200107**). 32211535 10.1016/j.aninu.2019.12.001 PMC7083746

[111] Yin L, Vijaygopal P, Menon R, Vaught LA, Zhang M, Zhang L, et al. An amino acid mixture mitigates radiation-induced gastrointestinal toxicity. Health Phys. 2014;106(6):734–44. 10.1097/hp.0000000000000117. 24776907 10.1097/HP.0000000000000117

[112] Yin L, Gupta R, Vaught L, Grosche A, Okunieff P, Vidyasagar S. An amino acid-based oral rehydration solution (AA-ORS) enhanced intestinal epithelial proliferation in mice exposed to radiation. Sci Rep. 2016;6:37220. 10.1038/srep37220. (**Epub 20161123**). 27876791 10.1038/srep37220 PMC5120277

[113] Rehrer NJ, Goes E, DuGardeyn C, Reynaert H, DeMeirleir K. Effect of carbohydrate on portal vein blood flow during exercise. Int J Sports Med. 2005;26(3):171–6. 10.1055/s-2004-820957. 15776331 10.1055/s-2004-820957

[114] Flood TR, Montanari S, Wicks M, Blanchard J, Sharp H, Taylor L, et al. Addition of pectin-alginate to a carbohydrate beverage does not maintain gastrointestinal barrier function during exercise in hot-humid conditions better than carbohydrate ingestion alone. Appl Physiol Nutr Metab. 2020;45(10):1145–55. 10.1139/apnm-2020-0118. (**Epub 20200504**). 32365303 10.1139/apnm-2020-0118

[115] Jonvik KL, Lenaerts K, Smeets JSJ, Kolkman JJ, van Loon LJC, Verdijk LB. Sucrose but not nitrate ingestion reduces strenuous cycling-induced intestinal injury. Med Sci Sports Exerc. 2019;51(3):436–44. 10.1249/mss.0000000000001800. 30299412 10.1249/MSS.0000000000001800

[116] Salvador AF, McKenna CF, Alamilla RA, Cloud RMT, Keeble AR, Miltko A, et al. Potato ingestion is as effective as carbohydrate gels to support prolonged cycling performance. J Appl Physiol. 2019;127(6):1651–9. 10.1152/japplphysiol.00567.2019. (**Epub 20191017**). 31622159 10.1152/japplphysiol.00567.2019 PMC6962613

[117] Houghton MJ, Snipe RMJ, Williamson G, Costa RJS. Plasma measurements of the dual sugar test reveal carbohydrate immediately alleviates intestinal permeability caused by exertional heat stress. J Physiol. 2023;601(20):4573–89. 10.1113/jp284536. (**Epub 20230911**). 37695123 10.1113/JP284536

[118] Sessions J, Bourbeau K, Rosinski M, Szczygiel T, Nelson R, Sharma N, et al. Carbohydrate gel ingestion during running in the heat on markers of gastrointestinal distress. Eur J Sport Sci. 2016;16(8):1064–72. 10.1080/17461391.2016.1140231. (**Epub 20160203**). 26841003 10.1080/17461391.2016.1140231

[119] Trommelen J, Fuchs CJ, Beelen M, Lenaerts K, Jeukendrup AE, Cermak NM, et al. Fructose and sucrose intake increase exogenous carbohydrate oxidation during exercise. Nutrients. 2017. 10.3390/nu9020167. (**Epub 20170220**).

[120] Kong S, Zhang YH, Zhang W. Regulation of intestinal epithelial cells properties and functions by amino acids. Biomed Res Int. 2018;2018:2819154. 10.1155/2018/2819154. (**Epub 20180509**). 29854738 10.1155/2018/2819154 PMC5966675

[121] Windmueller HG, Spaeth AE. Uptake and metabolism of plasma glutamine by the small intestine. J Biol Chem. 1974;249(16):5070–9. 4605420

[122] Pugh JN, Sage S, Hutson M, Doran DA, Fleming SC, Highton J, et al. Glutamine supplementation reduces markers of intestinal permeability during running in the heat in a dose-dependent manner. Eur J Appl Physiol. 2017;117(12):2569–77. 10.1007/s00421-017-3744-4. (**Epub 20171020**). 29058112 10.1007/s00421-017-3744-4 PMC5694515

[123] Tataka Y, Haramura M, Hamada Y, Ono M, Toyoda S, Yamada T, et al. Effects of oral cystine and glutamine on exercise-induced changes in gastrointestinal permeability and damage markers in young men. Eur J Nutr. 2022;61(5):2331–9. 10.1007/s00394-022-02806-1. (**Epub 20220201**). 35106632 10.1007/s00394-022-02806-1 PMC9279189

[124] Zuhl MN, Lanphere KR, Kravitz L, Mermier CM, Schneider S, Dokladny K, et al. Effects of oral glutamine supplementation on exercise-induced gastrointestinal permeability and tight junction protein expression. J Appl Physiol (1985). 2014;116(2):183–91. 10.1152/japplphysiol.00646.2013. (**Epub 20131127**). 24285149 10.1152/japplphysiol.00646.2013 PMC3921361

[125] Zuhl M, Dokladny K, Mermier C, Schneider S, Salgado R, Moseley P. The effects of acute oral glutamine supplementation on exercise-induced gastrointestinal permeability and heat shock protein expression in peripheral blood mononuclear cells. Cell Stress Chaperones. 2015;20(1):85–93. 10.1007/s12192-014-0528-1. (**Epub 20140726**). 25062931 10.1007/s12192-014-0528-1 PMC4255255

[126] Nava RC, Zuhl MN, Moriarty TA, Amorim FT, Bourbeau KC, Welch AM, et al. The effect of acute glutamine supplementation on markers of inflammation and fatigue during consecutive days of simulated wildland firefighting. J Occup Environ Med. 2019;61(2):e33–42. 10.1097/jom.0000000000001507. 30489352 10.1097/JOM.0000000000001507

[127] Osborne JO, Stewart IB, Beagley KW, Borg DN, Minett GM. Acute glutamine supplementation does not improve 20-km self-paced cycling performance in the heat. Eur J Appl Physiol. 2019;119(11–12):2567–78. 10.1007/s00421-019-04234-2. (**Epub 20190930**). 31565753 10.1007/s00421-019-04234-2

[128] Ogden HB, Fallowfield JL, Child RB, Davison G, Fleming SC, Delves SK, et al. No protective benefits of low dose acute L-glutamine supplementation on small intestinal permeability, epithelial injury and bacterial translocation biomarkers in response to subclinical exertional-heat stress: a randomized cross-over trial. Temperature (Austin). 2022;9(2):196–210. 10.1080/23328940.2021.2015227. (**Epub 20220107**). 36106146 10.1080/23328940.2021.2015227 PMC9467553

[129] Ogden HB, Fallowfield JL, Child RB, Davison G, Fleming SC, Delves SK, et al. Acute (L)-glutamine supplementation does not improve gastrointestinal permeability, injury or microbial translocation in response to exhaustive high intensity exertional-heat stress. Eur J Sport Sci. 2022;22(12):1865–76. 10.1080/17461391.2021.2001575. (**Epub 20211202**). 34726114 10.1080/17461391.2021.2001575

[130] Ogden HB, Child RB, Fallowfield JL, Delves SK, Westwood CS, Millyard A, et al. Gastrointestinal tolerance of low, medium and high dose acute oral l-glutamine supplementation in healthy adults: a pilot study. Nutrients. 2020. 10.3390/nu12102953. (**Epub 20200927**). 10.3390/nu12102953 PMC7601811 32992440

[131] van Wijck K, Wijnands KA, Meesters DM, Boonen B, van Loon LJ, Buurman WA, et al. L-citrulline improves splanchnic perfusion and reduces gut injury during exercise. Med Sci Sports Exerc. 2014;46(11):2039–46. 10.1249/mss.0000000000000332. 24621960 10.1249/MSS.0000000000000332

[132] Buchman AL, O’Brien W, Ou CN, Rognerud C, Alvarez M, Dennis K, et al. The effect of arginine or glycine supplementation on gastrointestinal function, muscle injury, serum amino acid concentrations and performance during a marathon run. Int J Sports Med. 1999;20(5):315–21. 10.1055/s-2007-971137. 10452229 10.1055/s-2007-971137

[133] Grimble GK. Adverse gastrointestinal effects of arginine and related amino acids. J Nutr. 2007;137(6 Suppl 2):1693s-s1701. 10.1093/jn/137.6.1693S. 17513449 10.1093/jn/137.6.1693S

[134] Rowlands DS, Clarke J, Green JG, Shi X. L-Arginine but not L-glutamine likely increases exogenous carbohydrate oxidation during endurance exercise. Eur J Appl Physiol. 2012;112(7):2443–53. 10.1007/s00421-011-2225-4. (**Epub 20111103**). 22048324 10.1007/s00421-011-2225-4

[135] Taylor G, Leonard A, Tang JCY, Dunn R, Fraser WD, Virgilio N, et al. The effects of collagen peptides on exercise-induced gastrointestinal stress: a randomized, controlled trial. Eur J Nutr. 2023;62(2):1027–39. 10.1007/s00394-022-03051-2. (**Epub 20221112**). 36370176 10.1007/s00394-022-03051-2 PMC9941265

[136] Costa RJS, Henningsen K, Gaskell SK, Alcock R, Mika A, Rauch C, et al. Amino acid-based beverage interventions ameliorate exercise-induced gastrointestinal syndrome in response to exertional-heat stress: the Heat Exertion Amino Acid Technology (HEAAT) study. Int J Sport Nutr Exerc Metab. 2023;33(4):230–42. 10.1123/ijsnem.2023-0025. (**Epub 20230524**). 37225167 10.1123/ijsnem.2023-0025

[137] Henningsen K, Henry R, Gaskell SK, Alcock R, Mika A, Rauch C, et al. Exertional heat stress promotes the presence of bacterial DNA in plasma: a counterbalanced randomised controlled trial. J Sci Med Sport. 2024;27(9):610–7. 10.1016/j.jsams.2024.05.010. (**Epub 20240522**). 38906729 10.1016/j.jsams.2024.05.010

[138] Choi W, Yeruva S, Turner JR. Contributions of intestinal epithelial barriers to health and disease. Exp Cell Res. 2017;358(1):71–7. 10.1016/j.yexcr.2017.03.036. (**Epub 20170323**). 28342899 10.1016/j.yexcr.2017.03.036 PMC5958612

[139] Rowlands DS, Wadsworth DP. No effect of protein coingestion on exogenous glucose oxidation during exercise. Med Sci Sports Exerc. 2012;44(4):701–8. 10.1249/MSS.0b013e318237e7c5. 21946154 10.1249/MSS.0b013e318237e7c5

[140] Lis DM, Stellingwerff T, Shing CM, Ahuja KD, Fell JW. Exploring the popularity, experiences, and beliefs surrounding gluten-free diets in nonceliac athletes. Int J Sport Nutr Exerc Metab. 2015;25(1):37–45. 10.1123/ijsnem.2013-0247. (**Epub 20140605**). 24901744 10.1123/ijsnem.2013-0247

[141] Sapone A, Bai JC, Ciacci C, Dolinsek J, Green PH, Hadjivassiliou M, et al. Spectrum of gluten-related disorders: consensus on new nomenclature and classification. BMC Med. 2012;10:13. 10.1186/1741-7015-10-13. (**Epub 20120207**). 22313950 10.1186/1741-7015-10-13 PMC3292448

[142] Uhde M, Ajamian M, Caio G, De Giorgio R, Indart A, Green PH, et al. Intestinal cell damage and systemic immune activation in individuals reporting sensitivity to wheat in the absence of celiac disease. Gut. 2016;65(12):1930–7. 10.1136/gutjnl-2016-311964. (**Epub 20160725**). 27459152 10.1136/gutjnl-2016-311964 PMC5136710

[143] Biesiekierski JR, Rosella O, Rose R, Liels K, Barrett JS, Shepherd SJ, et al. Quantification of fructans, galacto-oligosacharides and other short-chain carbohydrates in processed grains and cereals. J Hum Nutr Diet. 2011;24(2):154–76. 10.1111/j.1365-277X.2010.01139.x. (**Epub 20110221**). 21332832 10.1111/j.1365-277X.2010.01139.x

[144] Lis D, Ahuja KD, Stellingwerff T, Kitic CM, Fell J. Food avoidance in athletes: FODMAP foods on the list. Appl Physiol Nutr Metab. 2016;41(9):1002–4. 10.1139/apnm-2015-0428. (**Epub 20160511**). 27507006 10.1139/apnm-2015-0428

[145] Skodje GI, Sarna VK, Minelle IH, Rolfsen KL, Muir JG, Gibson PR, et al. Fructan, rather than gluten, induces symptoms in patients with self-reported non-celiac gluten sensitivity. Gastroenterology. 2018;154(3):529–39. 10.1053/j.gastro.2017.10.040. (**e2. Epub 20171102**). 29102613 10.1053/j.gastro.2017.10.040

[146] Staudacher HM, Whelan K, Irving PM, Lomer MC. Comparison of symptom response following advice for a diet low in fermentable carbohydrates (FODMAPs) versus standard dietary advice in patients with irritable bowel syndrome. J Hum Nutr Diet. 2011;24(5):487–95. 10.1111/j.1365-277X.2011.01162.x. (**Epub 20110525**). 21615553 10.1111/j.1365-277X.2011.01162.x

[147] Lis D, Stellingwerff T, Kitic CM, Ahuja KD, Fell J. No effects of a short-term gluten-free diet on performance in nonceliac athletes. Med Sci Sports Exerc. 2015;47(12):2563–70. 10.1249/mss.0000000000000699. 25970665 10.1249/MSS.0000000000000699

[148] Gibson PR, Halmos EP, Muir JG. Review article: FODMAPS, prebiotics and gut health-the FODMAP hypothesis revisited. Aliment Pharmacol Ther. 2020;52(2):233–46. 10.1111/apt.15818. (**Epub 20200620**). 32562590 10.1111/apt.15818

[149] Halmos EP, Power VA, Shepherd SJ, Gibson PR, Muir JG. A diet low in FODMAPs reduces symptoms of irritable bowel syndrome. Gastroenterology. 2014;146(1):67–75. 10.1053/j.gastro.2013.09.046. (**e5. Epub 20130925**). 24076059 10.1053/j.gastro.2013.09.046

[150] Marsh A, Eslick EM, Eslick GD. Does a diet low in FODMAPs reduce symptoms associated with functional gastrointestinal disorders? A comprehensive systematic review and meta-analysis. Eur J Nutr. 2016;55(3):897–906. 10.1007/s00394-015-0922-1. (**Epub 20150517**). 25982757 10.1007/s00394-015-0922-1

[151] Sperber AD. Review article: epidemiology of IBS and other bowel disorders of gut-brain interaction (DGBI). Aliment Pharmacol Ther. 2021;54(Suppl 1):S1-s11. 10.1111/apt.16582. 34927754 10.1111/apt.16582

[152] Lis DM, Stellingwerff T, Kitic CM, Fell JW, Ahuja KDK. Low FODMAP: a preliminary strategy to reduce gastrointestinal distress in athletes. Med Sci Sports Exerc. 2018;50(1):116–23. 10.1249/mss.0000000000001419. 28891824 10.1249/MSS.0000000000001419

[153] Halmos EP, Christophersen CT, Bird AR, Shepherd SJ, Gibson PR, Muir JG. Diets that differ in their FODMAP content alter the colonic luminal microenvironment. Gut. 2015;64(1):93–100. 10.1136/gutjnl-2014-307264. (**Epub 20140712**). 25016597 10.1136/gutjnl-2014-307264

[154] Hill P, Muir JG, Gibson PR. Controversies and recent developments of the low-FODMAP diet. Gastroenterol Hepatol (N Y). 2017;13(1):36–45. 28420945 PMC5390324

[155] Staudacher HM, Irving PM, Lomer MC, Whelan K. Mechanisms and efficacy of dietary FODMAP restriction in IBS. Nat Rev Gastroenterol Hepatol. 2014;11(4):256–66. 10.1038/nrgastro.2013.259. (**Epub 20140121**). 24445613 10.1038/nrgastro.2013.259

[156] Scrivin R, Slater GJ, Mika A, Rauch C, Young P, Martinez I, et al. The impact of 48-h high carbohydrate diets with high and low FODMAP content on gastrointestinal status and symptoms in response to endurance exercise, and subsequent endurance performance. Appl Physiol Nutr Metab. 2024. 10.1139/apnm-2023-0508. (**Epub 20240215**). 10.1139/apnm-2023-0508 38359412

[157] Burke LM. Ketogenic low-CHO, high-fat diet: the future of elite endurance sport? J Physiol. 2021;599(3):819–43. 10.1113/jp278928. (**Epub 20200610**). 32358802 10.1113/JP278928 PMC7891323

[158] Erridge C, Attina T, Spickett CM, Webb DJ. A high-fat meal induces low-grade endotoxemia: evidence of a novel mechanism of postprandial inflammation. Am J Clin Nutr. 2007;86(5):1286–92. 10.1093/ajcn/86.5.1286. 17991637 10.1093/ajcn/86.5.1286

[159] Lau E, Marques C, Pestana D, Santoalha M, Carvalho D, Freitas P, et al. The role of I-FABP as a biomarker of intestinal barrier dysfunction driven by gut microbiota changes in obesity. Nutr Metab (Lond). 2016;13:31. 10.1186/s12986-016-0089-7. (**Epub 20160430**). 27134637 10.1186/s12986-016-0089-7 PMC4851788

[160] Furuhashi M, Hotamisligil GS. Fatty acid-binding proteins: role in metabolic diseases and potential as drug targets. Nat Rev Drug Discov. 2008;7(6):489–503. 10.1038/nrd2589. 18511927 10.1038/nrd2589 PMC2821027

[161] McKay AKA, Wallett AM, McKune AJ, Périard JD, Saunders P, Whitfield J, et al. The impact of a short-term ketogenic low-carbohydrate high-fat diet on biomarkers of intestinal epithelial integrity and gastrointestinal symptoms. Int J Sport Nutr Exerc Metab. 2023;33(6):305–15. 10.1123/ijsnem.2023-0009. (**Epub 20230811**). 37567573 10.1123/ijsnem.2023-0009

[162] McKay AKA, Peeling P, Pyne DB, Tee N, Whitfield J, Sharma AP, et al. Six days of low carbohydrate, not energy availability, alters the iron and immune response to exercise in elite athletes. Med Sci Sports Exerc. 2022;54(3):377–87. 10.1249/mss.0000000000002819. 34690285 10.1249/MSS.0000000000002819

[163] Abraham S, Kellow JE. Do the digestive tract symptoms in eating disorder patients represent functional gastrointestinal disorders? BMC Gastroenterol. 2013;13:38. 10.1186/1471-230x-13-38. (**Epub 20130228**). 23448363 10.1186/1471-230X-13-38 PMC3606125

[164] Norris ML, Harrison ME, Isserlin L, Robinson A, Feder S, Sampson M. Gastrointestinal complications associated with anorexia nervosa: a systematic review. Int J Eat Disord. 2016;49(3):216–37. 10.1002/eat.22462. (**Epub 20150926**). 26407541 10.1002/eat.22462

[165] Mountjoy M, Ackerman KE, Bailey DM, Burke LM, Constantini N, Hackney AC, et al. 2023 International Olympic Committee’s (IOC) consensus statement on relative energy deficiency in sport (REDs). Br J Sports Med. 2023;57(17):1073–97. 10.1136/bjsports-2023-106994. 37752011 10.1136/bjsports-2023-106994

[166] Ackerman KE, Holtzman B, Cooper KM, Flynn EF, Bruinvels G, Tenforde AS, et al. Low energy availability surrogates correlate with health and performance consequences of relative energy deficiency in sport. Br J Sports Med. 2019;53(10):628–33. 10.1136/bjsports-2017-098958. (**Epub 20180602**). 29860237 10.1136/bjsports-2017-098958

[167] Black CJ, Yuan Y, Selinger CP, Camilleri M, Quigley EMM, Moayyedi P, et al. Efficacy of soluble fiber, antispasmodic drugs, and gut-brain neuromodulators in irritable bowel syndrome: a systematic review and network meta-analysis. Lancet Gastroenterol Hepatol. 2020;5(2):117–31. 10.1016/s2468-1253(19)30324-3. (**Epub 20191216**). 31859183 10.1016/S2468-1253(19)30324-3

[168] Camilleri M. Diagnosis and treatment of irritable bowel syndrome: a review. JAMA. 2021;325(9):865–77. 10.1001/jama.2020.22532. 33651094 10.1001/jama.2020.22532

[169] Fitzpatrick JA, Melton SL, Yao CK, Gibson PR, Halmos EP. Dietary management of adults with IBD—The emerging role of dietary therapy. Nat Rev Gastroenterol Hepatol. 2022;19(10):652–69. 10.1038/s41575-022-00619-5. (**Epub 20220516**). 35577903 10.1038/s41575-022-00619-5

[170] Galica AN, Galica R, Dumitrașcu DL. Diet, fibers, and probiotics for irritable bowel syndrome. J Med Life. 2022;15(2):174–9. 10.25122/jml-2022-0028. 35419092 10.25122/jml-2022-0028 PMC8999090

[171] Gill SK, Rossi M, Bajka B, Whelan K. Dietary fiber in gastrointestinal health and disease. Nat Rev Gastroenterol Hepatol. 2021;18(2):101–16. 10.1038/s41575-020-00375-4. (**Epub 20201118**). 33208922 10.1038/s41575-020-00375-4

[172] Sabo CM, Simiras C, Ismaiel A, Dumitrascu DL. Diet and gut inflammation: the effect of diet on inflammatory markers in inflammatory bowel disease—a scoping review. J Gastrointestin Liver Dis. 2023;32(3):402–10. 10.15403/jgld-5090. (**Epub 20230928**). 37494554 10.15403/jgld-5090

[173] Burke LM, Castell LM, Casa DJ, Close GL, Costa RJS, Desbrow B, et al. International Association of Athletics Federations Consensus Statement 2019: nutrition for athletics. Int J Sport Nutr Exerc Metab. 2019;29(2):73–84. 10.1123/ijsnem.2019-0065. (**Epub 20190405**). 30952204 10.1123/ijsnem.2019-0065

[174] Qiu X, Dong K, Guan J, He J. Hydrogen attenuates radiation-induced intestinal damage by reducing oxidative stress and inflammatory responses. Int Immunopharmacol. 2020;84: 106517. 10.1016/j.intimp.2020.106517. 32361189 10.1016/j.intimp.2020.106517

[175] Yoshikawa T, Yasuda M, Ueda S, Naito Y, Tanigawa T, Oyamada H, et al. Vitamin E in gastric mucosal injury induced by ischemia-reperfusion. Am J Clin Nutr. 1991;53(1 Suppl):210s-s214. 10.1093/ajcn/53.1.210S. 1985390 10.1093/ajcn/53.1.210S

[176] Ashton T, Young IS, Davison GW, Rowlands CC, McEneny J, Van Blerk C, et al. Exercise-induced endotoxemia: the effect of ascorbic acid supplementation. Free Radic Biol Med. 2003;35(3):284–91. 10.1016/s0891-5849(03)00309-5. 12885590 10.1016/s0891-5849(03)00309-5

[177] Buchman AL, Killip D, Ou CN, Rognerud CL, Pownall H, Dennis K, et al. Short-term vitamin E supplementation before marathon running: a placebo-controlled trial. Nutrition. 1999;15(4):278–83. 10.1016/s0899-9007(99)00005-2. 10319359 10.1016/s0899-9007(99)00005-2

[178] Srinivasan K. Biological activities of red pepper (Capsicum annuum) and its pungent principle capsaicin: a review. Crit Rev Food Sci Nutr. 2016;56(9):1488–500. 10.1080/10408398.2013.772090. 25675368 10.1080/10408398.2013.772090

[179] Opheim MN, Rankin JW. Effect of capsaicin supplementation on repeated sprinting performance. J Strength Cond Res. 2012;26(2):319–26. 10.1519/JSC.0b013e3182429ae5. 22130402 10.1519/JSC.0b013e3182429ae5

[180] Rauch CE, Mika AS, McCubbin AJ, Huschtscha Z, Costa RJS. Effect of prebiotics, probiotics, and synbiotics on gastrointestinal outcomes in healthy adults and active adults at rest and in response to exercise—a systematic literature review. Front Nutr. 2022;9:1003620. 10.3389/fnut.2022.1003620. (**Epub 20221207**). 36570133 10.3389/fnut.2022.1003620 PMC9768503

[181] Gurunathan S, Thangaraj P, Kim JH. Postbiotics: functional food materials and therapeutic agents for cancer, diabetes, and inflammatory diseases. 2023. Foods. 10.3390/foods13010089. (**Epub 20231226**). 10.3390/foods13010089 PMC10778838 38201117

[182] Salminen S, Collado MC, Endo A, Hill C, Lebeer S, Quigley EMM, et al. The International Scientific Association of Probiotics and Prebiotics (ISAPP) consensus statement on the definition and scope of postbiotics. Nat Rev Gastroenterol Hepatol. 2021;18(9):649–67. 10.1038/s41575-021-00440-6. (**Epub 20210504**). 33948025 10.1038/s41575-021-00440-6 PMC8387231

[183] Thorakkattu P, Khanashyam AC, Shah K, Babu KS, Mundanat AS, Deliephan A, et al. Postbiotics: current trends in food and pharmaceutical industry. 2022. Foods. 10.3390/foods11193094. (**Epub 20221005**). 10.3390/foods11193094 PMC9564201 36230169

[184] Rauch C, Henningsen, K, Martinez, I, Young, P, Mika, A, Huschtscha, Z, et al. The effect of prebiotic supplementation on markers of exercise-induced gastrointestinal syndrome in response to exertional-heat stress. Int J Sport Nutr Exerc Metab. 2025 (**In press**). https://doi.org/10.1123/ijsnem.2024-0127 10.1123/ijsnem.2024-0127 40010361

[185] Ajamian M, Steer D, Rosella G, Gibson PR. Serum zonulin as a marker of intestinal mucosal barrier function: may not be what it seems. PLoS ONE. 2019;14(1): e0210728. 10.1371/journal.pone.0210728. (**Epub 20190114**). 30640940 10.1371/journal.pone.0210728 PMC6331146

[186] Power N, Turpin W, Espin-Garcia O, Smith MI, Croitoru K. Serum zonulin measured by commercial kit fails to correlate with physiologic measures of altered gut permeability in first degree relatives of Crohn’s disease patients. Front Physiol. 2021;12: 645303. 10.3389/fphys.2021.645303. (**Epub 20210325**). 33841181 10.3389/fphys.2021.645303 PMC8027468

[187] Scheffler L, Crane A, Heyne H, Tönjes A, Schleinitz D, Ihling CH, et al. Widely used commercial ELISA does not detect precursor of haptoglobin2, but recognizes properdin as a potential second member of the zonulin family. Front Endocrinol (Lausanne). 2018;9:22. 10.3389/fendo.2018.00022. (**Epub 20180205**). 29459849 10.3389/fendo.2018.00022 PMC5807381

[188] Tatucu-Babet OA, Forsyth A, Owen E, Navarro-Perez D, Radcliffe J, Benheim D, et al. Serum zonulin measured by enzyme-linked immunosorbent assay may not be a reliable marker of small intestinal permeability in healthy adults. Nutr Res. 2020;78:82–92. 10.1016/j.nutres.2020.05.003. (**Epub 20200519**). 32563954 10.1016/j.nutres.2020.05.003

[189] Gill SK, Allerton DM, Ansley-Robson P, Hemmings K, Cox M, Costa RJ. Does short-term high dose probiotic supplementation containing Lactobacillus casei attenuate exertional-heat stress induced endotoxaemia and cytokinaemia? Int J Sport Nutr Exerc Metab. 2016;26(3):268–75. 10.1123/ijsnem.2015-0186. (**Epub 20151116**). 26568577 10.1123/ijsnem.2015-0186

[190] Yang M, Zou Y, Wu ZH, Li SL, Cao ZJ. Colostrum quality affects immune system establishment and intestinal development of neonatal calves. J Dairy Sci. 2015;98(10):7153–63. 10.3168/jds.2014-9238. (**Epub 20150729**). 26233454 10.3168/jds.2014-9238

[191] Kim JW, Jeon WK, Kim EJ. Combined effects of bovine colostrum and glutamine in diclofenac-induced bacterial translocation in rat. Clin Nutr. 2005;24(5):785–93. 10.1016/j.clnu.2005.04.004. 15919136 10.1016/j.clnu.2005.04.004

[192] Prosser C, Stelwagen K, Cummins R, Guerin P, Gill N, Milne C. Reduction in heat-induced gastrointestinal hyperpermeability in rats by bovine colostrum and goat milk powders. J Appl Physiol. 2004;96(2):650–4. 10.1152/japplphysiol.00295.2003. (**Epub 20031003**). 14527963 10.1152/japplphysiol.00295.2003

[193] Davison G, Marchbank T, March DS, Thatcher R, Playford RJ. Zinc carnosine works with bovine colostrum in truncating heavy exercise-induced increase in gut permeability in healthy volunteers. Am J Clin Nutr. 2016;104(2):526–36. 10.3945/ajcn.116.134403. (**Epub 20160629**). 27357095 10.3945/ajcn.116.134403

[194] Marchbank T, Davison G, Oakes JR, Ghatei MA, Patterson M, Moyer MP, et al. The nutriceutical bovine colostrum truncates the increase in gut permeability caused by heavy exercise in athletes. Am J Physiol Gastrointest Liver Physiol. 2011;300(3):G477-84. 10.1152/ajpgi.00281.2010. (**Epub 20101209**). 21148400 10.1152/ajpgi.00281.2010

[195] March DS, Marchbank T, Playford RJ, Jones AW, Thatcher R, Davison G. Intestinal fatty acid-binding protein and gut permeability responses to exercise. Eur J Appl Physiol. 2017;117(5):931–41. 10.1007/s00421-017-3582-4. (**Epub 20170313**). 28290057 10.1007/s00421-017-3582-4 PMC5388720

[196] March DS, Jones AW, Thatcher R, Davison G. The effect of bovine colostrum supplementation on intestinal injury and circulating intestinal bacterial DNA following exercise in the heat. Eur J Nutr. 2019;58(4):1441–51. 10.1007/s00394-018-1670-9. (**Epub 20180324**). 29574607 10.1007/s00394-018-1670-9 PMC6561991

[197] McKenna Z, Berkemeier Q, Naylor A, Kleint A, Gorini F, Ng J, et al. Bovine colostrum supplementation does not affect plasma I-FABP concentrations following exercise in a hot and humid environment. Eur J Appl Physiol. 2017;117(12):2561–7. 10.1007/s00421-017-3743-5. (**Epub 20171024**). 29063949 10.1007/s00421-017-3743-5

[198] Morrison SA, Cheung SS, Cotter JD. Bovine colostrum, training status, and gastrointestinal permeability during exercise in the heat: a placebo-controlled double-blind study. Appl Physiol Nutr Metab. 2014;39(9):1070–82. 10.1139/apnm-2013-0583. (**Epub 20140611**). 25068884 10.1139/apnm-2013-0583

[199] Buckley JD, Butler RN, Southcott E, Brinkworth GD. Bovine colostrum supplementation during running training increases intestinal permeability. Nutrients. 2009;1(2):224–34. 10.3390/nu1020224. (**Epub 20091202**). 22253980 10.3390/nu1020224 PMC3257608

[200] Carol A, Witkamp RF, Wichers HJ, Mensink M. Bovine colostrum supplementation’s lack of effect on immune variables during short-term intense exercise in well-trained athletes. Int J Sport Nutr Exerc Metab. 2011;21(2):135–45. 10.1123/ijsnem.21.2.135. 21558575 10.1123/ijsnem.21.2.135

[201] Davison G. The use of bovine colostrum in sport and exercise. Nutrients. 2021. 10.3390/nu13061789. (**Epub 20210524**). 10.3390/nu13061789 PMC8225123 34073917

[202] Główka N, Durkalec-Michalski K, Woźniewicz M. Immunological outcomes of bovine colostrum supplementation in trained and physically active people: a systematic review and meta-analysis. Nutrients. 2020. 10.3390/nu12041023. (**Epub 20200408**). 10.3390/nu12041023 PMC7231218 32276466

[203] Ghosh SS, Bie J, Wang J, Ghosh S. Oral supplementation with non-absorbable antibiotics or curcumin attenuates western diet-induced atherosclerosis and glucose intolerance in LDLR−/− mice—Role of intestinal permeability and macrophage activation. PLoS ONE. 2014;9(9): e108577. 10.1371/journal.pone.0108577. (**Epub 20140924**). 25251395 10.1371/journal.pone.0108577 PMC4177397

[204] Lin Y, Liu H, Bu L, Chen C, Ye X. Review of the effects and mechanism of curcumin in the treatment of inflammatory bowel disease. Front Pharmacol. 2022;13: 908077. 10.3389/fphar.2022.908077. (**Epub 20220620**). 35795556 10.3389/fphar.2022.908077 PMC9250976

[205] Wang J, Ghosh SS, Ghosh S. Curcumin improves intestinal barrier function: modulation of intracellular signaling, and organization of tight junctions. Am J Physiol Cell Physiol. 2017;312(4):C438-c45. 10.1152/ajpcell.00235.2016. (**Epub 20170301**). 28249988 10.1152/ajpcell.00235.2016 PMC5407015

[206] Szymanski MC, Gillum TL, Gould LM, Morin DS, Kuennen MR. Short-term dietary curcumin supplementation reduces gastrointestinal barrier damage and physiological strain responses during exertional heat stress. J Appl Physiol (1985). 2018;124(2):330–40. 10.1152/japplphysiol.00515.2017. (**Epub 20170921**). 28935827 10.1152/japplphysiol.00515.2017

[207] Biedermann L, Mwinyi J, Scharl M, Frei P, Zeitz J, Kullak-Ublick GA, et al. Bilberry ingestion improves disease activity in mild to moderate ulcerative colitis—an open pilot study. J Crohns Colitis. 2013;7(4):271–9. 10.1016/j.crohns.2012.07.010. (**Epub 20120809**). 22883440 10.1016/j.crohns.2012.07.010

[208] Li S, Wu B, Fu W, Reddivari L. The anti-inflammatory effects of dietary anthocyanins against ulcerative colitis. Int J Mol Sci. 2019. 10.3390/ijms20102588. (**Epub 20190527**). 10.3390/ijms20102588 PMC6567294 31137777

[209] Nair AR, Masson GS, Ebenezer PJ, Del Piero F, Francis J. Role of TLR4 in lipopolysaccharide-induced acute kidney injury: protection by blueberry. Free Radic Biol Med. 2014;71:16–25. 10.1016/j.freeradbiomed.2014.03.012. (**Epub 20140319**). 24657730 10.1016/j.freeradbiomed.2014.03.012

[210] Roth S, Spalinger MR, Gottier C, Biedermann L, Zeitz J, Lang S, et al. Bilberry-derived anthocyanins modulate cytokine expression in the intestine of patients with ulcerative colitis. PLoS ONE. 2016;11(5): e0154817. 10.1371/journal.pone.0154817. (**Epub 20160506**). 27152519 10.1371/journal.pone.0154817 PMC4859486

[211] Nakamura Y, Matsumoto H, Morifuji M, Iida H, Takeuchi Y. Development and validation of a liquid chromatography tandem mass spectrometry method for simultaneous determination of four anthocyanins in human plasma after black currant anthocyanins ingestion. J Agric Food Chem. 2010;58(2):1174–9. 10.1021/jf9027365. 20028128 10.1021/jf9027365

[212] Lyall KA, Hurst SM, Cooney J, Jensen D, Lo K, Hurst RD, et al. Short-term blackcurrant extract consumption modulates exercise-induced oxidative stress and lipopolysaccharide-stimulated inflammatory responses. Am J Physiol Regul Integr Comp Physiol. 2009;297(1):R70-81. 10.1152/ajpregu.90740.2008. (**Epub 20090429**). 19403859 10.1152/ajpregu.90740.2008

[213] Lee BJ, Flood TR, Hiles AM, Walker EF, Wheeler LEV, Ashdown KM, et al. Anthocynin-rich blackcurrent extract preserves gastrointestinal barrier permeability and reduces enterocyte damage but has no effect on microbial translocation and inflammation after exertional heat stress. Int J Sport Nutr Exerc Metab. 2022;32(4):265–74. 10.1123/ijsnem.2021-0330. 35287112 10.1123/ijsnem.2021-0330

[214] Matheson PJ, Wilson MA, Spain DA, Harris PD, Anderson GL, Garrison RN. Glucose-induced intestinal hyperemia is mediated by nitric oxide. J Surg Res. 1997;72(2):146–54. 10.1006/jsre.1997.5176. 9356236 10.1006/jsre.1997.5176

[215] Goulet EDB, Hoffman MD. Impact of ad libitum versus programmed drinking on endurance performance: a systematic review with meta-analysis. Sports Med. 2019;49(2):221–32. 10.1007/s40279-018-01051-z. 30659500 10.1007/s40279-018-01051-z

[216] Costa RJS, Knechtle B, Tarnopolsky M, Hoffman MD. Nutrition for ultramarathon running: trail, track, and road. Int J Sport Nutr Exerc Metab. 2019;29(2):130–40. 10.1123/ijsnem.2018-0255. (**Epub 20190403**). 30943823 10.1123/ijsnem.2018-0255

[217] Hoffman MD, Stellingwerff T, Costa RJS. Considerations for ultra-endurance activities: part 2—Hydration. Res Sports Med. 2019;27(2):182–94. 10.1080/15438627.2018.1502189. (**Epub 20180728**). 30056755 10.1080/15438627.2018.1502189

[218] McCubbin A, Costa RJS. Impact of sodium ingestion during exercise on endurance performance: a systematic review. Int J Sports Sci. 2018;8(3):97–107.

[219] McCubbin AJ, Lopez MB, Cox GR, Caldwell Odgers JN, Costa RJS. Impact of 3-day high and low dietary sodium intake on sodium status in response to exertional-heat stress: a double-blind randomized control trial. Eur J Appl Physiol. 2019;119(9):2105–18. 10.1007/s00421-019-04199-2. (**Epub 20190803**). 31377851 10.1007/s00421-019-04199-2

[220] McCubbin AJ, da Costa RJS. Effect of personalized sodium replacement on fluid and sodium balance and thermophysiological strain during and after ultraendurance running in the heat. Int J Sports Physiol Perform. 2024;19(2):105–15. 10.1123/ijspp.2023-0295. (**Epub 20231109**). 37944507 10.1123/ijspp.2023-0295

[221] van Nieuwenhoven MA, Vriens BE, Brummer RJ, Brouns F. Effect of dehydration on gastrointestinal function at rest and during exercise in humans. Eur J Appl Physiol. 2000;83(6):578–84. 10.1007/s004210000305. 11192068 10.1007/s004210000305

[222] Kartaram S, Mensink M, Teunis M, Schoen E, Witte G, Janssen Duijghuijsen L, et al. Plasma citrulline concentration, a marker for intestinal functionality, reflects exercise intensity in healthy young men. Clin Nutr. 2019;38(5):2251–8. 10.1016/j.clnu.2018.09.029. (**Epub 20180928**). 30340895 10.1016/j.clnu.2018.09.029

[223] Plunkett BT, Hopkins WG. Investigation of the side pain “stitch” induced by running after fluid ingestion. Med Sci Sports Exerc. 1999;31(8):1169–75. 10.1097/00005768-199908000-00014. 10449020 10.1097/00005768-199908000-00014

[224] Jardine WT, Aisbett B, Kelly MK, Burke LM, Ross ML, Condo D, et al. The effect of pre-exercise hyperhydration on exercise performance, physiological outcomes and gastrointestinal symptoms: a systematic review. Sports Med. 2023;53(11):2111–34. 10.1007/s40279-023-01885-2. (**Epub 20230725**). 37490269 10.1007/s40279-023-01885-2 PMC10587316

[225] Lambert GP, Lang J, Bull A, Pfeifer PC, Eckerson J, Moore G, et al. Fluid restriction during running increases GI permeability. Int J Sports Med. 2008;29(3):194–8. 10.1055/s-2007-965163. (**Epub 20070705**). 17614027 10.1055/s-2007-965163

[226] Périard JD, Eijsvogels TMH, Daanen HAM. Exercise under heat stress: thermoregulation, hydration, performance implications, and mitigation strategies. Physiol Rev. 2021;101(4):1873–979. 10.1152/physrev.00038.2020. (**Epub 20210408**). 33829868 10.1152/physrev.00038.2020

[227] Kuennen M, Gillum T, Dokladny K, Bedrick E, Schneider S, Moseley P. Thermotolerance and heat acclimation may share a common mechanism in humans. Am J Physiol Regul Integr Comp Physiol. 2011;301(2):R524-33. 10.1152/ajpregu.00039.2011. (**Epub 20110525**). 21613575 10.1152/ajpregu.00039.2011 PMC3154710

[228] Barberio MD, Elmer DJ, Laird RH, Lee KA, Gladden B, Pascoe DD. Systemic LPS and inflammatory response during consecutive days of exercise in heat. Int J Sports Med. 2015;36(3):262–70. 10.1055/s-0034-1389904. (**Epub 20141219**). 25525952 10.1055/s-0034-1389904

[229] Lee BJ, Thake CD. Heat and hypoxic acclimation increase monocyte heat shock protein 72 but do not attenuate inflammation following hypoxic exercise. Front Physiol. 2017;8:811. 10.3389/fphys.2017.00811. 29085305 10.3389/fphys.2017.00811 PMC5650636

[230] Sumi D, Nagatsuka H, Matsuo K, Okazaki K, Goto K. The impact of heat acclimation on gastrointestinal function following endurance exercise in a hot environment. Nutrients. 2023. 10.3390/nu15010216. (**Epub 20230101**). 10.3390/nu15010216 PMC9823684 36615873

[231] Snipe RMJ, Costa RJS. Does the temperature of water ingested during exertional-heat stress influence gastrointestinal injury, symptoms, and systemic inflammatory profile? J Sci Med Sport. 2018;21(8):771–6. 10.1016/j.jsams.2017.12.014. (**Epub 20180108**). 29371075 10.1016/j.jsams.2017.12.014

[232] Stevens CJ, Dascombe B, Boyko A, Sculley D, Callister R. Ice slurry ingestion during cycling improves Olympic distance triathlon performance in the heat. J Sports Sci. 2013;31(12):1271–9. 10.1080/02640414.2013.779740. (**Epub 20130318**). 23506436 10.1080/02640414.2013.779740

[233] Alhadad SB, Chua MCY, Lee JKW, Low ICC. The effects of low and normal dose ice slurry ingestion on endurance capacity and intestinal epithelial injury in the heat. J Sci Med Sport. 2023;26(6):278–84. 10.1016/j.jsams.2023.04.008. (**Epub 20230429**). 37179242 10.1016/j.jsams.2023.04.008

[234] Lee BJ, Clarke ND, Hankey J, Thake CD. Whole body precooling attenuates the extracellular HSP72, IL-6 and IL-10 responses after an acute bout of running in the heat. J Sports Sci. 2018;36(4):414–21. 10.1080/02640414.2017.1313441. (**Epub 20170405**). 28376678 10.1080/02640414.2017.1313441

[235] Duffield R, Steinbacher G, Fairchild TJ. The use of mixed-method, part-body pre-cooling procedures for team-sport athletes training in the heat. J Strength Cond Res. 2009;23(9):2524–32. 10.1519/JSC.0b013e3181bf7a4f. 19910821 10.1519/JSC.0b013e3181bf7a4f

[236] Castellani JW, Young AJ. Human physiological responses to cold exposure: acute responses and acclimatization to prolonged exposure. Auton Neurosci. 2016;196:63–74. 10.1016/j.autneu.2016.02.009. (**Epub 20160221**). 26924539 10.1016/j.autneu.2016.02.009

[237] Costa RJ, Smith AH, Oliver SJ, Walters R, Maassen N, Bilzon JL, et al. The effects of two nights of sleep deprivation with or without energy restriction on immune indices at rest and in response to cold exposure. Eur J Appl Physiol. 2010;109(3):417–28. 10.1007/s00421-010-1378-x. (**Epub 20100206**). 20140447 10.1007/s00421-010-1378-x

[238] Oliver SJ, Harper Smith AD, Costa RJ, Maassen N, Bilzon JL, Walsh NP. Two nights of sleep deprivation with or without energy restriction does not impair the thermal response to cold. Eur J Appl Physiol. 2015;115(10):2059–68. 10.1007/s00421-015-3184-y. (**Epub 20150521**). 25995099 10.1007/s00421-015-3184-y

[239] Walsh NP, Whitham M. Exercising in environmental extremes: a greater threat to immune function? Sports Med. 2006;36(11):941–76. 10.2165/00007256-200636110-00003. 17052132 10.2165/00007256-200636110-00003

[240] Rehrer NJ, Brouns F, Beckers EJ, ten Hoor F, Saris WH. Gastric emptying with repeated drinking during running and bicycling. Int J Sports Med. 1990;11(3):238–43. 10.1055/s-2007-1024799. 2373584 10.1055/s-2007-1024799

[241] Martinez IG, Mika AS, Biesiekierski JR, Costa RJS. The effect of gut-training and feeding-challenge on markers of gastrointestinal status in response to endurance exercise: a systematic literature review. Sports Med. 2023;53(6):1175–200. 10.1007/s40279-023-01841-0. (**Epub 20230415**). 37061651 10.1007/s40279-023-01841-0 PMC10185635

[242] Collier R. Competitive consumption: a profusion of pie, pizza and pulled pork. CMAJ. 2013;185(4):290–1. 10.1503/cmaj.109-4394. (**Epub 20130114**). 23318403 10.1503/cmaj.109-4394 PMC3589305

[243] Geliebter A, Yahav EK, Gluck ME, Hashim SA. Gastric capacity, test meal intake, and appetitive hormones in binge eating disorder. Physiol Behav. 2004;81(5):735–40. 10.1016/j.physbeh.2004.04.014. 15234178 10.1016/j.physbeh.2004.04.014

[244] Levine MS, Spencer G, Alavi A, Metz DC. Competitive speed eating: truth and consequences. Am J Roentgenol. 2007;189(3):681–6. 10.2214/AJR.07.2342. 17715117 10.2214/AJR.07.2342

[245] Lambert GP, Lang J, Bull A, Eckerson J, Lanspa S, O’Brien J. Fluid tolerance while running: effect of repeated trials. Int J Sports Med. 2008;29(11):878–82. 10.1055/s-2008-1038620. (**Epub 20080529**). 18512180 10.1055/s-2008-1038620

[246] Castiglione KE, Read NW, French SJ. Adaptation to high-fat diet accelerates emptying of fat but not carbohydrate test meals in humans. Am J Physiol Regul Integr Comp Physiol. 2002;282(2):R366–71. 10.1152/ajpregu.00190.2001. 11792645 10.1152/ajpregu.00190.2001

[247] Cunningham KM, Horowitz M, Read NW. The effect of short-term dietary supplementation with glucose on gastric emptying in humans. Br J Nutr. 1991;65(1):15–9. 10.1079/bjn19910061. 1997128 10.1079/bjn19910061

[248] Horowitz M, Cunningham KM, Wishart JM, Jones KL, Read NW. The effect of short-term dietary supplementation with glucose on gastric emptying of glucose and fructose and oral glucose tolerance in normal subjects. Diabetologia. 1996;39(4):481–6. 10.1007/bf00400681. 8777999 10.1007/BF00400681

[249] Dyer J, Daly K, Salmon KS, Arora DK, Kokrashvili Z, Margolskee RF, et al. Intestinal glucose sensing and regulation of intestinal glucose absorption. Biochem Soc Trans. 2007;35(Pt 5):1191–4. 10.1042/bst0351191. 17956309 10.1042/BST0351191

[250] Margolskee RF, Dyer J, Kokrashvili Z, Salmon KS, Ilegems E, Daly K, et al. T1R3 and gustducin in gut sense sugars to regulate expression of Na+-glucose cotransporter 1. Proc Natl Acad Sci U S A. 2007;104(38):15075–80. 10.1073/pnas.0706678104. (**Epub 20070827**). 17724332 10.1073/pnas.0706678104 PMC1986615

[251] Miyamoto K, Hase K, Takagi T, Fujii T, Taketani Y, Minami H, et al. Differential responses of intestinal glucose transporter mRNA transcripts to levels of dietary sugars. Biochem J. 1993;295(Pt 1):211–5. 10.1042/bj2950211. 8216218 10.1042/bj2950211 PMC1134840

[252] Lin HC, Doty JE, Reedy TJ, Meyer JH. Inhibition of gastric emptying by glucose depends on length of intestine exposed to nutrient. Am J Physiol. 1989;256(2 Pt 1):G404–11. 10.1152/ajpgi.1989.256.2.G404. 2919683 10.1152/ajpgi.1989.256.2.G404

[253] Minami H, McCallum RW. The physiology and pathophysiology of gastric emptying in humans. Gastroenterology. 1984;86(6):1592–610. 6370777

[254] Cox GR, Clark SA, Cox AJ, Halson SL, Hargreaves M, Hawley JA, et al. Daily training with high carbohydrate availability increases exogenous carbohydrate oxidation during endurance cycling. J Appl Physiol (1985). 2010;109(1):126–34. 10.1152/japplphysiol.00950.2009. (**Epub 20100513**). 20466803 10.1152/japplphysiol.00950.2009

[255] Martinez IG, Houghton MJ, Forte M, Williamson G, Biesiekierski JR, Costa RJS. Development of a low-fructose carbohydrate gel for exercise application. Heliyon. 2024;10(13): e33497. 10.1016/j.heliyon.2024.e33497. 39040322 10.1016/j.heliyon.2024.e33497 PMC11260965

[256] Conde-Pipó J, Mora-Fernandez A, Martinez-Bebia M, Gimenez-Blasi N, Lopez-Moro A, Latorre JA, et al. Intermittent fasting: does it affect sports performance? A systematic review. Nutrients. 2024. 10.3390/nu16010168. (**Epub 20240104**). 10.3390/nu16010168 PMC10780856 38201996

[257] Ferraris RP. Dietary and developmental regulation of intestinal sugar transport. Biochem J. 2001;360(Pt 2):265–76. 10.1042/0264-6021:3600265. 11716754 10.1042/0264-6021:3600265 PMC1222226

[258] Stellingwerff T, Godin JP, Beaumont M, Tavenard A, Grathwohl D, van Bladeren PJ, et al. Effects of pre-exercise sucralose ingestion on carbohydrate oxidation during exercise. Int J Sport Nutr Exerc Metab. 2013;23(6):584–92. 10.1123/ijsnem.23.6.584. (**Epub 20130520**). 23689036 10.1123/ijsnem.23.6.584

[259] Costa RJS, Gaskell SK, McCubbin AJ, Snipe RMJ. Exertional-heat stress-associated gastrointestinal perturbations during Olympic sports: management strategies for athletes preparing and competing in the 2020 Tokyo Olympic Games. Temperature (Austin). 2020;7(1):58–88. 10.1080/23328940.2019.1597676. (**Epub 20190507**). 32166105 10.1080/23328940.2019.1597676 PMC7053925

[260] Hoffman MD, Pasternak A, Rogers IR, Khodaee M, Hill JC, Townes DA, et al. Medical services at ultra-endurance foot races in remote environments: medical issues and consensus guidelines. Sports Med. 2014;44(8):1055–69. 10.1007/s40279-014-0189-3. 24748459 10.1007/s40279-014-0189-3

