# Supplementary material for: Sports Dietitians Australia and Ultra Sports Science Foundation Joint Position Statement: A Practitioner Guide to the Prevention and Management of Exercise-Associated Gastrointestinal Perturbations and Symptoms
Source: Sports Med. 2025 Apr 7;55(5):1097–134. doi: 10.1007/s40279-025-02186-6 (PMC12106582; doi:10.1007/s40279-025-02186-6)
Supplement: Supplementary file 1 — Supplementary file1 (PDF 236 KB) [file 40279_2025_2186_MOESM1_ESM.pdf]

Sports Medicine

Supplementary File 1

Sports Dietitians Australia and Ultra Sports Science Foundation Joint Position Statement: A practitioner guide to the prevention and management of exercise-associated gastrointestinal perturbations and symptoms.

Ricardo J.S. Costa[1], Stephanie Gaskell[1], Kayla Henningsen[1], Nikki Jeacocke[2], Isabel Martinez[1], Alice Mika[1], Volker Scheer[3], Rachel Scrivin[4,5], Rhiannon Snipe[6], Alice Wallett[2], Pascale Young[1].

[1] Department of Nutrition Dietetics & Food, Monash University, Notting Hill, Victoria, Australia; [2] AIS Performance, Bruce, Canberra, Australia; [3] Ultra Sports Science Foundation, Pierre-Benite, France; [4] University of the Sunshine Coast, Sippy Downs, Queensland, Australia; [5] Toi Ohomai Institute of Technology, Tauranga, New Zealand; [6] School of Exercise and Nutrition Sciences, Deakin University, Burwood, Victoria, Australia.

Feeding tolerance

Intake volume, carbohydrate concentration and dose, texture form (i.e., liquid, semi-solid, and solid), carbohydrate type (i.e., mono-, di-, poly-saccharide, singular or multiple combinations, and/or specifically engineered), inclusion of caffeine, and other carbohydrate sources (i.e., coconut water and dairy) have been investigated and proposed to potentially effect gastrointestinal status, mainly in relation to either reducing or accentuating Ex-GIS incidence and/or severity, as a result of impacting (i.e., alleviating or burdening) gastrointestinal functional responses. This is of prime importance from a performance perspective, considering Ex-GIS incidence and severity has been shown to reduce exercise performance,[1, 2] and result in lowering workload and/or withdrawing from competition.[3-5] It is, however, acknowledged that exploration into Ex-GIS within research that investigated these various aspects of feeding tolerance is with caution due to the wide variety of heterogenous GIS assessment tools applied. Although some studies appear to use Ex-GIS assessment tool with background validity and reliability, and are in accordance with clinical gastroenterology research (e.g., ROME criteria for GIs types); a proportion appear to be unreferenced in-house fabrications, out dated, not in accordance with established clinical gastroenterology assessment procedures, non-validated and/or not reliability checked.[6]

Intake volume

Considering the limited capacity of the gastric chamber, it is plausible that after reaching its intragastric pressure threshold capacity, gastric function may be affected, resulting in subsequent signs and symptoms of an overburdened stomach. Several studies have investigated the effect of different volumes of a carbohydrate-electrolyte beverage provided every 10-20 min during 60-120 min of steady state or time trial running or cycling, on Ex-GIS, including the subjective sensation of stomach fullness, which is a subjective surrogate indicative of

intragastric pressure.[7-10] Overall, study findings suggest that the intake of carbohydrate-electrolyte beverage ≥900 mL/h during moderate to high intensity endurance exercise may not be well tolerated, compared with lower intake volumes (i.e., *ad libitum* intake obtaining 300-400 mL/h, or direct comparative volumes of 120 mL/h and 450 mL/h). Such higher intake volumes were reported to promote Ex-GIS, especially upper-GIS (e.g., upper abdominal discomfort, sensation of fullness, and/or nausea); and when a time trial was implemented as part of the exercise protocol, this led to participant withdrawal from the exercise protocol in one study due to Ex-GIS.[8] More recently, a randomised cross-over study that investigated water restriction to induce hypohydration versus programmed water intake (i.e., 844 mL/h evenly distributed every 15 min) to maintain euhydration during 2 h of steady state running at 70% $\dot{V}O_{2max}$ in thermoneutral conditions, reported high gut discomfort and upper-GIS in the programmed-euhydrated intake arm of the trial compared with restriction-hypohydration arm.[11] The increase in gut discomfort and upper-GIS was especially evident in the 1st hour of the exercise protocol. From a practical perspective, the requirement for fluid intake, to support hydration and/or energy provisions, during short duration high intensity exercise (e.g., ≤1 h at ≥70% $\dot{V}O_{2max}$) is relatively low in comparison to prolonged duration exercise (e.g., ≥2 h at ≤70% $\dot{V}O_{2max}$). Therefore, *ad libitum* or programmed low to moderate fluid intake volumes (e.g. ~500 mL/h) may be more appropriate for short duration high intensity exercise where the risk of exercise-associated dehydration, to reach a state of hypohydration, is relatively low. However, irrespective of exercise duration, intensity, and/or environmental conditions, when body water losses (i.e., sweating) substantially surpasses gastric tolerance ability of the individual during prolonged duration exercise, strategies such as repetitive gastric load challenge and/or 'gut-training' may be required to improve fluid volume tolerance (Section 9).

Grade of evidence: I

Carbohydrate concentration

Increasing the concentration of carbohydrate in a solution, beyond an individual's gastric and intestinal threshold, may delay gastric emptying, intestinal transit, and/or saturate intestinal nutrient transporters. Within an exercise context, this may accumulate Ex-GIS that can consequently have a negative impact on exercise performance.[1, 2, 12, 13] A substantial number of studies have investigated the effect of ingesting carbohydrate beverage concentrations from 3% to 12% *w/v* before, early into, and/or in multiple boluses during exercise (i.e., 4-20 min intervals) on Ex-GIS using a variety of exercise protocols.[14-19] Exercise protocols ranged from mixed circuit type exercise, 23 min to 120 min steady state exercise (i.e., running, cycling, or rowing) at 60-70% $\dot{V}O_{2max}$, with or without a subsequent time trial performance test. Overall general consensus from these studies suggests that the higher the carbohydrate concentration, typically >6% *w/v*, the greater incidence and severity of Ex-GIS, in comparison to lower carbohydrate concentrations (<6% *w/v*), in its simplistic explanation. However, an exploration into a set of experimental trials has provided some evidence that carbohydrate intake concentrations of 10% to 16% *w/v* during prolonged steady state exercise are well tolerated in majority of study participants, reporting low Ex-GIS and good feeding tolerance.[20] From a practical perspective, these observations highlight the importance to consider intake volume, and potentially carbohydrate form (i.e., liquid, semi-solid, or solid) and type (i.e., singular or multiple transportable carbohydrates, polysaccharides, or hydrogels), in assessing the true impact of carbohydrate concentration on Ex-GIS at an individual athlete level.

Grade of evidence: I

Carbohydrate dose and frequency

Similar to carbohydrate concentration, the total intake dose of the carbohydrate in a solution beyond an individual's gastric and intestinal threshold may delay gastric emptying, intestinal transit, and/or saturate intestinal nutrient transporters. Compared to a moderate 1.0 gCHO/min, a higher 1.4 gCHO/min did not increase Ex-GIS, but did result in a small increase in nausea during a 16 km outdoor running event simulation.[21] Large individual variation was observed, with n=3 runners reporting severe Ex-GIS with the higher dose, and n=1 runner reporting severe Ex-GIS with both high and low doses. While it is plausible that high carbohydrate dose may increase Ex-GIS, the frequency of delivery may impact the extent of Ex-GIS. A high carbohydrate (2.4 g/min; 24% *w/v*) and moderate carbohydrate (1.2 g/min; 12% *w/v*) solution (1:1 maltodextrin:fructose) was compared with either high frequency feeding (6 feeds) or low frequency feeding (2 feeds), during a 30 km treadmill roller skiing time trial in well-trained cross-country skiers.[22] High carbohydrate low frequency feeding significantly increased gut discomfort compared with high carbohydrate high frequency feeding and moderate carbohydrate ingested with either low or high frequency feeding. These study outcomes suggest that if high exogenous carbohydrate feedings are required during the exercise activities, increasing the frequency of intake to 'smaller and frequent' may mitigate feeding tolerance linked Ex-GIS.

Grade of evidence: III for dose and frequency

Intake texture form

Dependant of the sports and/or activity type (e.g., adventure racing or expedition), individuals consume carbohydrate rich fluids and/or foods during exercise in different texture forms, including liquids (e.g., carbohydrate-electrolyte beverages), semi-solids (e.g., gels or purees), and solids (e.g., bars or conventional foods). Liquid-based carbohydrate forms are highly adopted by the majority of sports, generally lasting <2 h. However, as the duration of the sports

or exercise activity increases, demand for solid carbohydrate forms increases in response to flavour/taste fatigue of the partaking individual.[23, 24] Considering intake nutrient density and residues may impact on gastric emptying rate and/or intestinal transit and absorption, is it plausible to suggest that the incidence and severity risk for Ex-GIS increases with the density of the food and/or fluid intake. Studies that investigate the impact of carbohydrate intake texture forms on Ex-GIS have used substantially prolonged exercise bout, ranging from 3 h steady state cycling or running at 75% $\dot{V}O_{2max}$, 2 h undulating cycling challenge (60-85% $\dot{V}o_{2peak}$) followed by a time trial, a long course triathlon, and 140 min cycling race simulation with an ending slow ramp to exhaustion.[25-28] Liquid, semi-solid (i.e., gels or potato purée), and/or solid (i.e., bar, banana, and white bread) carbohydrate forms were compared at standardised intake concentration ranging from 60-81 g/h, but frequency of intake varied due to the nature of the exercise protocols (e.g., continues cycling and running vs segmented triathlon). The research outcome suggests that consuming carbohydrate in solid and semi-solid forms may provoke greater Ex-GIS (i.e., nausea, stomach fullness, abdominal bloating and pain, urge to defecate and defecation) compared to carbohydrate in liquid form.

Grade of evidence: I

Carbohydrate type

Within professional practice there is a substantial array of carbohydrate supplement products on the commercial market targeting during exercise energy provisions. The spectrum ranges from single monosaccharide type products, monosaccharide combinations, and specifically engineered carbohydrate types (e.g., maltodextrin, modified starch, and hydrogel). It is argued in the literature that the use of selective carbohydrate types may prevent Ex-GIS, through lowering the risk of gastrointestinal functional issues, such as easing gastric emptying, intestinal transit and absorption, and subsequent improved glucose availability in

circulation.[20] Whereas, the use of other carbohydrate types may exacerbate Ex-GIS, through impacting gastric emptying and/or increasing the risk of intestinal enterocyte transporter saturation leading to malabsorption and subsequent local bacterial fermentation, with or without ileal brake mechanisms.[2, 29-32] For example, it has been reported that consuming ~76 g/h (6% *w/v*) fructose during a 115 min intensive steady state (65-80% $\dot{V}O_{2max}$) cycling protocol, followed by a time trial, substantially increases Ex-GIS compared with consuming equivalent glucose and sucrose,[33] which was likely associated with fructose malabsorption and bacterial fermentation processes, as explore in Section 9.1.[1] Glucose polymers of differing molecular weight have also been studied for their potential role in reducing gastrointestinal burden (e.g., improving gastric emptying and intestinal transit), with the aim of reducing Ex-GIS. In general, the intake of high molecular weight carbohydrate (8-11% *w/v* amylopectin waxy maize starch) during 2.5 h cycling at 50-55% W_{max} did not result in any substantially greater Ex-GIS incidence and/or severity when compared with a low molecular weight carbohydrate (8-11% *w/v* maltodextrin).[34, 35] Interestingly, when the intake of 1.7 g/min of maltodextrin (10% *w/v*) was compared with 1.1 g/min maltodextrin + 0.6 g/min fructose (10% *w/v*) and a non-nutritive placebo during 2.5 h cycle at 50% $\dot{V}O_{2max}$ followed by a 60 km time trial, Ex-GIS (i.e., bloating and belching) was reported to be greater after the 1.7 g/min of maltodextrin trial.[36] Moreover, another study showed that the intake of maltodextrin and dextrose (5.6% *w/v*) before, during and immediately after a 70% $\dot{V}O_{2max}$ ergometer row of 2 x 25 min led to greater severity of gastroesophageal reflux and a greater sensation of stomach fullness compared with the intake of maltodextrin and sucrose (7.0%).[37] These study examples provide some indication that an overwhelming intake with one type of carbohydrate may exacerbate Ex-GIS, but combinations that result in a mixed monosaccharide intestinal lumen content may be more beneficial in reducing Ex-GIS, although modestly.

Considering the different intestinal enterocyte transport mechanism of monosaccharides (e.g., active SGLT for glucose and galactose, and passive GLUT5 for fructose) at the intestinal lumen apical surface, plus the absorption of fructose being potentially facilitated by the inclusion of glucose within the feeding bolus, as reported in the gastroenterology literature using resting clinical and health population models;[38-42] the application of multiple transportable carbohydrate blends (e.g., 2:1 glucose-fructose or 1:1:1 sucrose-glucose-fructose) during exercise primarily for enhancing glucose availability, muscular fuel provisions, and aiding enhancement of exercise performance, has previously been proposed and explored.[43-45] From an Ex-GIS perspective, studies that employed 2 h steady state exercise followed by a time trial have reported reduced Ex-GIS (e.g., belching, abdominal bloating and pain, flatulence, and nausea) with application of multiple versus singular carbohydrate solutions.[46, 47] Albeit, comparing isomaltulose (63 g/h) with a fructose:glucose mixture (63 g/h at 0.8:1.0 ratio) and resulting in more pronounced so-called 'stomach cramps' (i.e., unestablished term), bloating (i.e., undefined if upper or lower abdominal), and modestly greater nausea, at 120 min steady state cycling at 60% W_{max} and/or after the 16 km time trial with isomaltulose.[46] In addition, comparing maltodextrin:fructose (~78 g/h at 1.2:1 ratio) and matched maltodextrin:dextrose mixtures, resulted in the likelihood of reduced Ex-GIS with maltodextrin:fructose in response to 2 h steady state running followed by a 4 mile time trial.[47] However, equivocal outcomes have been reported in studies that have investigated glucose availability and fuel kinetics in response to singular and multiple-transportable carbohydrates, but in adjunct have attempted to explore Ex-GIS. Data presented generally shows no substantial to modestly lower incidence and/or severity of Ex-GIS with glucose-fructose or glucose-sucrose blends (79 g/h to 108 g/h) compared with matching glucose sources in response to both laboratory and field-based endurance exercise.[47-54] These studies, however, are not without limitations on Ex-GIS assessment and interpretation, which cautions their application into

professional practice, that include, but not limited to: 1) Ex-GIS confounder control in experimental procedures, in which the lack of pre-trial dietary provisions is a key factor (Section 5), and appears to be synonymous across multiple-transportable carbohydrate and fuel kinetics research; but well established that such uncontrolled methods as ‘participants record intake beforehand and attempt to duplicate intake on any subsequent trial/s’ appears inadequate for controlling dietary associated Ex-GIS instigators.[55] 2) The diverse Ex-GIS assessment tools employed, some of which are in-house fabrication with no clear background origin and unreferenced, erroneous terminologies and symptom types (i.e., also including limited and selective symptom types), not validated and reliability checked, and interpreted using either inferential or descriptive incidence reporting that shows little translational meaning when magnitude of difference it taken into consideration.[6, 56] 3) The study participant populations predominantly consist of small numbers (i.e., n= 8-10) of highly trained (e.g., ≥60 mL/kg/min $\dot{V}O_{2max}$) male endurance athletes, that do not reflect the bulk of general sport and exercise population that purchase and consume these carbohydrate rich products during exercise. Furthermore, it is important to consider individual responses to such multiple-transportable carbohydrate regimes, especially in the non-elite but highly active recreational athlete population, as those with potential known or unknown gastrointestinal functional issues may not respond to such an approach,[42] as shown with 64% of participants reporting malabsorption of 30 g/20min (90 g/h) 2:1 glucose:fructose ratio that correlated with Ex-GIS.[2] Therefore, at present, despite some previous studies suggesting that multiple-transportable carbohydrate blends reduce Ex-GIS, compared to a single carbohydrate sources, , findings seem unclear and modest at best, especially when exercise duration is ≥3 h and entering the ultra-endurance space.

Other types of carbohydrates that have also been investigated include modified starch and carbohydrate hydrogel. A slow digestible hydrothermally modified starch regime of 60 g 30 min pre-exercise then feeding every 15 min (60 g/h), and 60 g 30 min pre-exercise then feeding every 60 min (30 g/h) within a 3 h cycling experimental trial (50-80% W_{max} with maximal sprints) showed higher nausea severity in the modified starch groups compared with a commercially available sucrose-glucose supplement (60 g 30 min pre-exercise then feeding every 15 min (60 g/h).[57] No difference in other reported Ex-GIS (i.e., abdominal cramp and fullness) was observed between modified starch feeding regimes and glucose. More recently, there has been interest within the general sports population, especially endurance sports that warrant exogenous carbohydrate provisions, for the application of carbohydrate hydrogel claiming to accelerate gastric emptying, and subsequent intestinal absorption, circulatory glucose availability, and skeletal muscle oxidation, with emphasis of improving endurance exercise performance.[58] The micro-engineering and mechanisms of action include the addition of alginate and pectin into the carbohydrate supplement that forms a hydrogel structure in the low pH environment of the stomach, which encapsulates other local constituents (i.e., carbohydrate content), then returning to a liquid consistency in the higher pH environment of the duodenum.[59, 60] Therefore, in theory, the provision of similar or more exogenous carbohydrate content without the gastric burden and associated Ex-GIS. Although some preliminary research has reported enhanced gastric emptying rate with carbohydrate beverages containing hydrogel application versus standard glucose or maltodextrin plus fructose solutions,[58] it is important to note that these reports are at rest and not in response to exertional stress, where gastrointestinal functional responses are compromised *per se*, irrespective of exercise duration, intensity, and/or ambient conditions.[12, 61, 62] The original study that comprehensively investigated the effects of hydrogel carbohydrate supplementation versus matched carbohydrate supplementation (53 g/hr maltodextrin + 37 g/hr fructose, 16%

w/v) in response to 3 h running at 60% $\dot{V}O_{2max}$ followed by an incremental time to exhaustion test, on gastrointestinal tolerance, Ex-GIS, carbohydrate malabsorption and glucose availability, whole body carbohydrate oxidation, and exercise performance, reported no difference in all measured markers between the two carbohydrate interventions.[63] Follow-on studies, as reported in a systematic literature review, found similar results and confirms that hydrogel formulation has no beneficial effects on reducing Ex-GIS.[64] One proceeding study reported significantly less Ex-GIS with a hydrogel formulation (90 g/h 2:1 glucose-fructose) compared with non-hydrogel comparator, in high level male endurance runners (<2h40min marathon or >60 mL/kg/min $\dot{V}O_{2max}$).[65] However, the lack of controlled dietary provisions as a key confounding factor for Ex-GIS (Section 5), apparent in-house fabricated symptoms assessment tool, with no background reference point for tool evolution or production evident, and erroneous symptoms types and categorisation (e.g., urge to urinate as an Ex-GIS, stomach cramps categorised at lower-GIS, and headache categorised at systemic),[6, 56] does not allow such modest differences between hydrogel and non-hydrogel for Ex-GIS to be meaningful or translatable into practice. For example, using the same Ex-GIS assessment tool and lack of dietary provisions resulted in modestly greater stomach fullness in the alginate-pectin hydrogel trial versus the non-hydrogel trial in another study.[66]

Grade of evidence: I to IV dependent on the carbohydrate type.

Carbohydrate with caffeine

There is a general consensus within the public domain that caffeine *per se* is a gastrointestinal irritant, with its frequent and dose dependant consumption resulting in both upper- and lower-GIS.[67-69] The role of caffeine as an adenosine receptor inhibitor leads to a cascade of neuromodulatory effects on endogenous hormones and neurotransmitters (e.g., noradrenalin, dopamine, acetylcholine, and/or serotonin), which have direct effects on gastrointestinal

motility and exocrine secretions. It is therefore plausible that the inclusion of caffeine within pre- and/or during-exercise feeding food and fluid choices may perturbate gastrointestinal functional responses during exercise and aggravate Ex-GIS. The pre- and during-exercise administration of caffeine (15 mg/100mL) within a carbohydrate solution (6.9%), during 90 min cycling at 70% W_{max}, did not alter intra-gastric status or emptying, orocaecal transit time, and intestinal permeability, compared with a carbohydrate solution alone.[70] In additional, caffeine administration, ranging from a pre-exercise dose of 100 mg to 15 mg/100mL or 353 mg/h during endurance exercise did not further exacerbate Ex-GIS incidence and severity, compared with water or carbohydrate alone, resulting in similar Ex-GIS incidence type (i.e., bloating, belching, and flatulence), and modest Ex-GIS severity in a variety of exercise models.[71-73] For example, Ex-GIS incidence rates during an 18 km running competition were similar when runners ingested carbohydrate (90%; 69 g) or carbohydrate-caffeine (89%; 69 g + 150 mg) solution, but incidence rate was greater than water (79%).[72] While there are a small number of studies investigating the effect of caffeinated pre- and/or during-exercise, and/or inclusion in carbohydrate containing beverages on gastrointestinal integrity and functional responses, and Ex-GIS; the addition of caffeine within the applied dose does not appear to affect gastrointestinal integrity or function, or exacerbate Ex-GIS. However, individual tolerance and dose administration is advisable due to caffeine hypersensitivity in certain individuals.[74]

Grade of evidence: I

Coconut water and dairy

Individual studies have explored the effect of coconut water (i.e., carbohydrate at 5% *w/v*) during 60 min cycling at 45-65% maximum minute power,[75] and pre-exercise dairy-based meal before 80 min cycling at 60% W_{max} followed by a 10 min time trial,[76] on Ex-GIS.

Findings from individual studies make it difficult to draw firm conclusions; however, consumption of coconut water during exercise, and pre-exercise consumption of dairy did not increase Ex-GIS compared to consumption of water during exercise and pre-exercise carbohydrate control, respectively. It may be of interest to note that high pre-exercise intakes of dairy will likely contain a substantial lactose dose, which may exacerbate Ex-GIS severity in susceptible individuals (e.g., lactose intolerance), and subsequently should be avoided and alternatives applied in such individuals. On the contrary, dairy milk has consistently shown to be well tolerated in athletes from a gastrointestinal perspective, both pre-exercise and in the recovery period,[11, 79-82] so for the majority of the athlete population there is no evidence to advocate widespread avoidance.

Grade of evidence: III for coconut and dairy.

References

1. Costa RJS, Miall A, Khoo A, Rauch C, Snipe R, Camões-Costa V, et al. Gut-training: the impact of two weeks repetitive gut-challenge during exercise on gastrointestinal status, glucose availability, fuel kinetics, and running performance. Appl Physiol Nutr Metab. 2017;42(5):547-57. Epub 20170322. doi: 10.1139/apnm-2016-0453. PubMed PMID: 28177715.

2. Miall A, Khoo A, Rauch C, Snipe RMJ, Camões-Costa VL, Gibson PR, et al. Two weeks of repetitive gut-challenge reduce exercise-associated gastrointestinal symptoms and malabsorption. Scand J Med Sci Sports. 2018;28(2):630-40. Epub 20170619. doi: 10.1111/sms.12912. PubMed PMID: 28508559.

3. Costa RJ, Snipe R, Camões-Costa V, Scheer V, Murray A. The impact of gastrointestinal symptoms and dermatological injuries on nutritional intake and hydration status during ultramarathon events. Sports Med Open. 2016;2:16. Epub 20160105. doi: 10.1186/s40798-015-0041-9. PubMed PMID: 26767151; PubMed Central PMCID: PMCPMC4701764.

4. Jeukendrup AE, Vet-Joop K, Sturk A, Stegen JH, Senden J, Saris WH, et al. Relationship between gastro-intestinal complaints and endotoxaemia, cytokine release and the acute-phase reaction during and after a long-distance triathlon in highly trained men. Clin Sci (Lond). 2000;98(1):47-55. PubMed PMID: 10600658.

5. Pfeiffer B, Stellingwerff T, Hodgson AB, Randell R, Pöttgen K, Res P, et al. Nutritional intake and gastrointestinal problems during competitive endurance events. Med Sci Sports Exerc. 2012;44(2):344-51. doi: 10.1249/MSS.0b013e31822dc809. PubMed PMID: 21775906.

6. Costa RJS, Young P, Gill SK, Snipe RMJ, Gaskell S, Russo I, et al. Assessment of exercise-associated gastrointestinal perturbations in research and practical settings: Methodological concerns and recommendations for best practice. Int J Sport Nutr Exerc Metab.

2022;32(5):387-418. Epub 20220813. doi: 10.1123/ijsnem.2022-0048. PubMed PMID: 35963615.

7. Backx K, van Someren KA, Palmer GS. One hour cycling performance is not affected by ingested fluid volume. Int J Sport Nutr Exerc Metab. 2003;13(3):333-42. doi: 10.1123/ijsnem.13.3.333. PubMed PMID: 14669933.

8. Daries HN, Noakes TD, Dennis SC. Effect of fluid intake volume on 2-h running performances in a 25 degrees C environment. Med Sci Sports Exerc. 2000;32(10):1783-9. doi: 10.1097/00005768-200010000-00019. PubMed PMID: 11039653.

9. Lambert GP, Lang J, Bull A, Eckerson J, Lanspa S, O'Brien J. Fluid tolerance while running: effect of repeated trials. Int J Sports Med. 2008;29(11):878-82. Epub 20080529. doi: 10.1055/s-2008-1038620. PubMed PMID: 18512180.

10. Silva RP, Mündel T, Altoé JL, Saldanha MR, Ferreira FG, Marins JC. Preexercise urine specific gravity and fluid intake during one-hour running in a thermoneutral environment - a randomized cross-over study. J Sports Sci Med. 2010;9(3):464-71. Epub 20100901. PubMed PMID: 24149642; PubMed Central PMCID: PMCPMC3761697.

11. Costa RJS, Camões-Costa V, Snipe RMJ, Dixon D, Russo I, Huschtscha Z. Impact of exercise-induced hypohydration on gastrointestinal integrity, function, symptoms, and systemic endotoxin and inflammatory profile. J Appl Physiol (1985). 2019;126(5):1281-91. Epub 20190321. doi: 10.1152/japplphysiol.01032.2018. PubMed PMID: 30896356.

12. Horner KM, Schubert MM, Desbrow B, Byrne NM, King NA. Acute exercise and gastric emptying: a meta-analysis and implications for appetite control. Sports Med. 2015;45(5):659-78. doi: 10.1007/s40279-014-0285-4. PubMed PMID: 25398225.

13. Leiper JB. Fate of ingested fluids: factors affecting gastric emptying and intestinal absorption of beverages in humans. Nutr Rev. 2015;73 Suppl 2:57-72. doi: 10.1093/nutrit/nuv032. PubMed PMID: 26290292.

14. Davis JM, Burgess WA, Slentz CA, Bartoli WP, Pate RR. Effects of ingesting 6% and 12% glucose/electrolyte beverages during prolonged intermittent cycling in the heat. Eur J Appl Physiol Occup Physiol. 1988;57(5):563-9. doi: 10.1007/bf00418463. PubMed PMID: 3396573.

15. Łagowska K, Podgórski T, Celińska E, Wiertel Ł, Kryściak J. A comparison of the effectiveness of commercial and natural carbohydrate–electrolyte drinks. Science & Sports. 2017;32(3):160-4.

16. Morton DP, Aragón-Vargas LF, Callister R. Effect of ingested fluid composition on exercise-related transient abdominal pain. Int J Sport Nutr Exerc Metab. 2004;14(2):197-208. doi: 10.1123/ijsnem.14.2.197. PubMed PMID: 15118193.

17. Murray R, Seifert JG, Eddy DE, Paul GL, Halaby GA. Carbohydrate feeding and exercise: effect of beverage carbohydrate content. Eur J Appl Physiol Occup Physiol. 1989;59(1-2):152-8. doi: 10.1007/bf02396594. PubMed PMID: 2583144.

18. Shi X, Horn MK, Osterberg KL, Stofan JR, Zachwieja JJ, Horswill CA, et al. Gastrointestinal discomfort during intermittent high-intensity exercise: effect of carbohydrate-electrolyte beverage. Int J Sport Nutr Exerc Metab. 2004;14(6):673-83. doi: 10.1123/ijsnem.14.6.673. PubMed PMID: 15657472.

19. Wallis GA, Yeo SE, Blannin AK, Jeukendrup AE. Dose-response effects of ingested carbohydrate on exercise metabolism in women. Med Sci Sports Exerc. 2007;39(1):131-8. doi: 10.1249/01.mss.0000241645.28467.d3. PubMed PMID: 17218895.

20. Rauch CE, McCubbin AJ, Gaskell SK, Costa RJS. Feeding tolerance, glucose availability, and whole-body total carbohydrate and fat oxidation in male endurance and ultra-endurance runners in response to prolonged exercise, consuming a habitual mixed macronutrient diet and carbohydrate feeding during exercise. Front Physiol. 2021;12:773054.

Epub 20220104. doi: 10.3389/fphys.2021.773054. PubMed PMID: 35058795; PubMed Central PMCID: PMCPMC8764139.

21. Pfeiffer B, Cotterill A, Grathwohl D, Stellingwerff T, Jeukendrup AE. The effect of carbohydrate gels on gastrointestinal tolerance during a 16-km run. Int J Sport Nutr Exerc Metab. 2009;19(5):485-503. doi: 10.1123/ijsnem.19.5.485. PubMed PMID: 19910651.

22. Stocks B, Betts JA, McGawley K. Effects of carbohydrate dose and frequency on metabolism, gastrointestinal discomfort, and cross-country skiing performance. Scand J Med Sci Sports. 2016;26(9):1100-8. Epub 20150827. doi: 10.1111/sms.12544. PubMed PMID: 26316418.

23. Costa RJS, Knechtle B, Tarnopolsky M, Hoffman MD. Nutrition for ultramarathon running: trail, track, and road. Int J Sport Nutr Exerc Metab. 2019;29(2):130-40. Epub 20190403. doi: 10.1123/ijsnem.2018-0255. PubMed PMID: 30943823.

24. Costa RJS, Hoffman MD, Stellingwerff T. Considerations for ultra-endurance activities: part 1- nutrition. Res Sports Med. 2019;27(2):166-81. Epub 20180728. doi: 10.1080/15438627.2018.1502188. PubMed PMID: 30056753.

25. Guillochon M, Rowlands DS. Solid, gel, and liquid carbohydrate format effects on gut comfort and performance. Int J Sport Nutr Exerc Metab. 2017;27(3):247-54. Epub 20161220. doi: 10.1123/ijsnem.2016-0211. PubMed PMID: 27997257.

26. Peters HP, van Schelven FW, Verstappen PA, de Boer RW, Bol E, Erich WB, et al. Gastrointestinal problems as a function of carbohydrate supplements and mode of exercise. Med Sci Sports Exerc. 1993;25(11):1211-24. PubMed PMID: 8289607.

27. Salvador AF, McKenna CF, Alamilla RA, Cloud RMT, Keeble AR, Miltko A, et al. Potato ingestion is as effective as carbohydrate gels to support prolonged cycling performance. J Appl Physiol (1985). 2019;127(6):1651-9. Epub 20191017. doi:

10.1152/japplphysiol.00567.2019. PubMed PMID: 31622159; PubMed Central PMCID: PMCPMC6962613.

28. Sareban M, Zügel D, Koehler K, Hartveg P, Zügel M, Schumann U, et al. Carbohydrate Intake in form of gel is associated with increased gastrointestinal distress but not with performance differences compared with liquid carbohydrate ingestion during simulated long-distance triathlon. Int J Sport Nutr Exerc Metab. 2016;26(2):114-22. Epub 20150831. doi: 10.1123/ijsnem.2015-0060. PubMed PMID: 26323018.

29. Layer P, Peschel S, Schlesinger T, Goebell H. Human pancreatic secretion and intestinal motility: effects of ileal nutrient perfusion. Am J Physiol. 1990;258(2 Pt 1):G196-201. doi: 10.1152/ajpgi.1990.258.2.G196. PubMed PMID: 1689548.

30. Shin HS, Ingram JR, McGill AT, Poppitt SD. Lipids, CHOs, proteins: can all macronutrients put a 'brake' on eating? Physiol Behav. 2013;120:114-23. Epub 20130801. doi: 10.1016/j.physbeh.2013.07.008. PubMed PMID: 23911804.

31. van Avesaat M, Troost FJ, Ripken D, Hendriks HF, Masclee AA. Ileal brake activation: macronutrient-specific effects on eating behavior? Int J Obes (Lond). 2015;39(2):235-43. Epub 20140624. doi: 10.1038/ijo.2014.112. PubMed PMID: 24957485.

32. Van Citters GW, Lin HC. Ileal brake: neuropeptidergic control of intestinal transit. Curr Gastroenterol Rep. 2006;8(5):367-73. doi: 10.1007/s11894-006-0021-9. PubMed PMID: 16968603.

33. Murray R, Paul GL, Seifert JG, Eddy DE, Halaby GA. The effects of glucose, fructose, and sucrose ingestion during exercise. Med Sci Sports Exerc. 1989;21(3):275-82. PubMed PMID: 2733576.

34. Rowlands DS, Clarke J. Lower oxidation of a high molecular weight glucose polymer vs. glucose during cycling. Appl Physiol Nutr Metab. 2011;36(2):298-306. doi: 10.1139/h11-006. PubMed PMID: 21609293.

35. Rowlands DS, Wallis GA, Shaw C, Jentjens RL, Jeukendrup AE. Glucose polymer molecular weight does not affect exogenous carbohydrate oxidation. Med Sci Sports Exerc. 2005;37(9):1510-6. doi: 10.1249/01.mss.0000177586.68399.f5. PubMed PMID: 16177602.
36. Roberts JD, Tarpey MD, Kass LS, Tarpey RJ, Roberts MG. Assessing a commercially available sports drink on exogenous carbohydrate oxidation, fluid delivery and sustained exercise performance. J Int Soc Sports Nutr. 2014;11(1):8. Epub 20140304. doi: 10.1186/1550-2783-11-8. PubMed PMID: 24589205; PubMed Central PMCID: PMCPMC3975841.
37. Lagowska K, Bajerska, J., Jeszka, J. The Effect of two carbohydrate-elektrolyte drinks on gastrointestinal complaints and physical performance in rowers. Medicina Sportiva 2009;13(3):171-6.
38. Latulippe ME, Skoog SM. Fructose malabsorption and intolerance: effects of fructose with and without simultaneous glucose ingestion. Crit Rev Food Sci Nutr. 2011;51(7):583-92. doi: 10.1080/10408398.2011.566646. PubMed PMID: 21793722; PubMed Central PMCID: PMCPMC3471321.
39. Murray K, Wilkinson-Smith V, Hoad C, Costigan C, Cox E, Lam C, et al. Differential effects of FODMAPs (fermentable oligo-, di-, mono-saccharides and polyols) on small and large intestinal contents in healthy subjects shown by MRI. Am J Gastroenterol. 2014;109(1):110-9. Epub 20131119. doi: 10.1038/ajg.2013.386. PubMed PMID: 24247211; PubMed Central PMCID: PMCPMC3887576.
40. Rumessen JJ, Gudmand-Høyer E. Absorption capacity of fructose in healthy adults. Comparison with sucrose and its constituent monosaccharides. Gut. 1986;27(10):1161-8. doi: 10.1136/gut.27.10.1161. PubMed PMID: 3781328; PubMed Central PMCID: PMCPMC1433856.

41. Truswell AS, Seach JM, Thorburn AW. Incomplete absorption of pure fructose in healthy subjects and the facilitating effect of glucose. Am J Clin Nutr. 1988;48(6):1424-30. doi: 10.1093/ajcn/48.6.1424. PubMed PMID: 3202090.

42. Tuck CJ, Ross LA, Gibson PR, Barrett JS, Muir JG. Adding glucose to food and solutions to enhance fructose absorption is not effective in preventing fructose-induced functional gastrointestinal symptoms: randomised controlled trials in patients with fructose malabsorption. J Hum Nutr Diet. 2017;30(1):73-82. Epub 20160907. doi: 10.1111/jhn.12409. PubMed PMID: 27600184.

43. Rowlands DS, Houltham S, Musa-Veloso K, Brown F, Paulionis L, Bailey D. Fructose-glucose composite carbohydrates and endurance performance: critical review and future perspectives. Sports Med. 2015;45(11):1561-76. doi: 10.1007/s40279-015-0381-0. PubMed PMID: 26373645.

44. Smith JW, Pascoe DD, Passe DH, Ruby BC, Stewart LK, Baker LB, et al. Curvilinear dose-response relationship of carbohydrate (0-120 g·h(-1)) and performance. Med Sci Sports Exerc. 2013;45(2):336-41. doi: 10.1249/MSS.0b013e31827205d1. PubMed PMID: 22968309.

45. Stellingwerff T, Cox GR. Systematic review: Carbohydrate supplementation on exercise performance or capacity of varying durations. Appl Physiol Nutr Metab. 2014;39(9):998-1011. Epub 20140325. doi: 10.1139/apnm-2014-0027. PubMed PMID: 24951297.

46. Oosthuyse T, Carstens M, Millen AM. Ingesting isomaltulose versus fructose-maltodextrin during prolonged moderate-heavy exercise increases fat oxidation but impairs gastrointestinal comfort and cycling performance. Int J Sport Nutr Exerc Metab. 2015;25(5):427-38. Epub 20150326. doi: 10.1123/ijsnem.2014-0178. PubMed PMID: 25811946.

47. Wilson PB, Ingraham SJ. Glucose-fructose likely improves gastrointestinal comfort and endurance running performance relative to glucose-only. Scand J Med Sci Sports. 2015;25(6):e613-20. Epub 20141230. doi: 10.1111/sms.12386. PubMed PMID: 25556817.

48. Rowlands DS, Thorburn MS, Thorp RM, Broadbent S, Shi X. Effect of graded fructose coingestion with maltodextrin on exogenous 14C-fructose and 13C-glucose oxidation efficiency and high-intensity cycling performance. J Appl Physiol (1985). 2008;104(6):1709-19. Epub 20080327. doi: 10.1152/japplphysiol.00878.2007. PubMed PMID: 18369092.

49. Rowlands DS, Houltham SD. Multiple-transportable carbohydrate effect on long-distance triathlon performance. Med Sci Sports Exerc. 2017;49(8):1734-44. doi: 10.1249/mss.0000000000001278. PubMed PMID: 28350714.

50. Baur DA, Schroer AB, Luden ND, Womack CJ, Smyth SA, Saunders MJ. Glucose-fructose enhances performance versus isocaloric, but not moderate, glucose. Med Sci Sports Exerc. 2014;46(9):1778-86. doi: 10.1249/mss.0000000000000284. PubMed PMID: 25134001.

51. Jentjens RL, Moseley L, Waring RH, Harding LK, Jeukendrup AE. Oxidation of combined ingestion of glucose and fructose during exercise. J Appl Physiol (1985). 2004;96(4):1277-84. Epub 20031202. doi: 10.1152/japplphysiol.00974.2003. PubMed PMID: 14657042.

52. Jentjens RL, Underwood K, Achten J, Currell K, Mann CH, Jeukendrup AE. Exogenous carbohydrate oxidation rates are elevated after combined ingestion of glucose and fructose during exercise in the heat. J Appl Physiol (1985). 2006;100(3):807-16. Epub 20051110. doi: 10.1152/japplphysiol.00322.2005. PubMed PMID: 16282436.

53. O'Brien WJ, Rowlands DS. Fructose-maltodextrin ratio in a carbohydrate-electrolyte solution differentially affects exogenous carbohydrate oxidation rate, gut comfort, and performance. Am J Physiol Gastrointest Liver Physiol. 2011;300(1):G181-9. Epub 20101111. doi: 10.1152/ajpgi.00419.2010. PubMed PMID: 21071509.

54. Rowlands DS, Swift M, Ros M, Green JG. Composite versus single transportable carbohydrate solution enhances race and laboratory cycling performance. Appl Physiol Nutr Metab. 2012;37(3):425-36. Epub 20120403. doi: 10.1139/h2012-013. PubMed PMID: 22468766.

55. Jeacocke NA, Burke LM. Methods to standardize dietary intake before performance testing. Int J Sport Nutr Exerc Metab. 2010;20(2):87-103. doi: 10.1123/ijsnem.20.2.87. PubMed PMID: 20479482.

56. Gaskell SK, Snipe RMJ, Costa RJS. Test-Retest reliability of a modified visual analog scale assessment tool for determining incidence and severity of gastrointestinal symptoms in response to exercise stress. Int J Sport Nutr Exerc Metab. 2019;29(4):411–9. Epub 20190701. doi: 10.1123/ijsnem.2018-0215. PubMed PMID: 30632417.

57. Baur DA, Vargas Fde C, Bach CW, Garvey JA, Ormsbee MJ. Slow-absorbing modified starch before and during prolonged cycling increases fat oxidation and gastrointestinal distress without changing performance. Nutrients. 2016;8(7). Epub 20160625. doi: 10.3390/nu8070392. PubMed PMID: 27347999; PubMed Central PMCID: PMCPMC4963868.

58. Sutehall S, Galloway SDR, Bosch A, Pitsiladis Y. Addition of an alginate hydrogel to a carbohydrate beverage enhances gastric emptying. Med Sci Sports Exerc. 2020;52(8):1785-92. doi: 10.1249/mss.0000000000002301. PubMed PMID: 32079920.

59. George M, Abraham TE. pH sensitive alginate-guar gum hydrogel for the controlled delivery of protein drugs. Int J Pharm. 2007;335(1-2):123-9. Epub 20061107. doi: 10.1016/j.ijpharm.2006.11.009. PubMed PMID: 17147980.

60. Lee KY, Mooney DJ. Alginate: properties and biomedical applications. Prog Polym Sci. 2012;37(1):106-26. doi: 10.1016/j.progpolymsci.2011.06.003. PubMed PMID: 22125349; PubMed Central PMCID: PMCPMC3223967.

61. Gaskell SK, Burgell R, Wiklendt L, Dinning P, Costa RJS. Does exertional heat stress impact gastrointestinal function and symptoms? J Sci Med Sport. 2022;25(12):960-7. Epub 20221017. doi: 10.1016/j.jsams.2022.10.008. PubMed PMID: 36347748.

62. Gaskell SK, Burgell R, Wiklendt L, Dinning PG, Costa RJS. Impact of exercise duration on gastrointestinal function and symptoms. J Appl Physiol (1985). 2023;134(1):160-71. Epub 20221208. doi: 10.1152/japplphysiol.00393.2022. PubMed PMID: 36476157.

63. McCubbin AJ, Zhu A, Gaskell SK, Costa RJS. Hydrogel carbohydrate-electrolyte beverage does not improve glucose availability, substrate oxidation, gastrointestinal symptoms or exercise performance, compared with a concentration and nutrient-matched placebo. Int J Sport Nutr Exerc Metab. 2020;30(1):25-33. doi: 10.1123/ijsnem.2019-0090. PubMed PMID: 31629348.

64. King AJ, Rowe JT, Burke LM. Carbohydrate hydrogel products do not improve performance or gastrointestinal distress during moderate-intensity endurance exercise. Int J Sport Nutr Exerc Metab. 2020;30(5):305-14. Epub 20200723. doi: 10.1123/ijsnem.2020-0102. PubMed PMID: 32707564.

65. Rowe JT, King R, King AJ, Morrison DJ, Preston T, Wilson OJ, et al. Glucose and fructose hydrogel enhances running performance, exogenous carbohydrate oxidation, and gastrointestinal tolerance. Med Sci Sports Exerc. 2022;54(1):129-40. doi: 10.1249/mss.0000000000002764. PubMed PMID: 34334720.

66. Mears SA, Worley J, Mason GS, Hulston CJ, James LJ. Addition of sodium alginate and pectin to a carbohydrate-electrolyte solution does not influence substrate oxidation, gastrointestinal comfort, or cycling performance. Appl Physiol Nutr Metab. 2020;45(6):675-8. Epub 20200122. doi: 10.1139/apnm-2019-0802. PubMed PMID: 31967853.

67. Boekema PJ, Samsom M, van Berge Henegouwen GP, Smout AJ. Coffee and gastrointestinal function: facts and fiction. A review. Scand J Gastroenterol Suppl. 1999;230:35-9. doi: 10.1080/003655299750025525. PubMed PMID: 10499460.

68. Duncanson KR, Talley NJ, Walker MM, Burrows TL. Food and functional dyspepsia: a systematic review. J Hum Nutr Diet. 2018;31(3):390-407. Epub 20170915. doi: 10.1111/jhn.12506. PubMed PMID: 28913843.

69. Nehlig A. Effects of coffee on the gastro-intestinal tract: A narrative review and literature update. Nutrients. 2022;14(2). Epub 20220117. doi: 10.3390/nu14020399. PubMed PMID: 35057580; PubMed Central PMCID: PMCPMC8778943.

70. Van Nieuwenhoven MA, Brummer RM, Brouns F. Gastrointestinal function during exercise: comparison of water, sports drink, and sports drink with caffeine. J Appl Physiol (1985). 2000;89(3):1079-85. doi: 10.1152/jappl.2000.89.3.1079. PubMed PMID: 10956354.

71. Scott AT, O'Leary T, Walker S, Owen R. Improvement of 2000-m rowing performance with caffeinated carbohydrate-gel ingestion. Int J Sports Physiol Perform. 2015;10(4):464-8. Epub 20141029. doi: 10.1123/ijspp.2014-0210. PubMed PMID: 25365032.

72. van Nieuwenhoven MA, Brouns F, Kovacs EM. The effect of two sports drinks and water on GI complaints and performance during an 18-km run. Int J Sports Med. 2005;26(4):281-5. doi: 10.1055/s-2004-820931. PubMed PMID: 15795812.

73. Yeo SE, Jentjens RL, Wallis GA, Jeukendrup AE. Caffeine increases exogenous carbohydrate oxidation during exercise. J Appl Physiol (1985). 2005;99(3):844-50. Epub 20050414. doi: 10.1152/japplphysiol.00170.2005. PubMed PMID: 15831802.

74. McKenzie YA, Bowyer RK, Leach H, Gulia P, Horobin J, O'Sullivan NA, et al. British Dietetic Association systematic review and evidence-based practice guidelines for the dietary management of irritable bowel syndrome in adults (2016 update). J Hum Nutr Diet. 2016;29(5):549-75. Epub 20160608. doi: 10.1111/jhn.12385. PubMed PMID: 27272325.

75. Peart DJ, Hensby A, Shaw MP. Coconut water does not improve markers of hydration during sub-maximal exercise and performance in a subsequent time trial compared with water alone. Int J Sport Nutr Exerc Metab. 2017;27(3):279-84. Epub 20161021. doi: 10.1123/ijsnem.2016-0121. PubMed PMID: 27768399.

76. Haakonssen EC, Ross ML, Cato LE, Nana A, Knight EJ, Jenkins DG, et al. Dairy-based preexercise meal does not affect gut comfort or time-trial performance in female cyclists. Int J Sport Nutr Exerc Metab. 2014;24(5):553-8. Epub 20140714. doi: 10.1123/ijsnem.2014-0069. PubMed PMID: 25029702.

77. Gaskell SK, Taylor B, Muir J, Costa RJS. Impact of 24-h high and low fermentable oligo-, di-, monosaccharide, and polyol diets on markers of exercise-induced gastrointestinal syndrome in response to exertional heat stress. Appl Physiol Nutr Metab. 2020;45(6):569-80. Epub 20191025. doi: 10.1139/apnm-2019-0187. PubMed PMID: 31652404.

78. Scrivin R, Slater GJ, Mika A, Rauch C, Young P, Martinez I, et al. The impact of 48-h high carbohydrate diets with high and low FODMAP content on gastrointestinal status and symptoms in response to endurance exercise, and subsequent endurance performance. Appl Physiol Nutr Metab. 2024. Epub 20240215. doi: 10.1139/apnm-2023-0508. PubMed PMID: 38359412.

79. Costa RJS, Camões-Costa V, Snipe RMJ, Dixon D, Russo I, Huschtscha Z. The impact of a dairy milk recovery beverage on bacterially stimulated neutrophil function and gastrointestinal tolerance in response to hypohydration inducing exercise stress. Int J Sport Nutr Exerc Metab. 2020;30(4):237-48. Epub 20200526. doi: 10.1123/ijsnem.2019-0349. PubMed PMID: 32460239.

80. Russo I, Della Gatta PA, Garnham A, Porter J, Burke LM, Costa RJS. Assessing overall exercise recovery processes using carbohydrate and carbohydrate-protein containing recovery

beverages. Front Physiol. 2021;12:628863. Epub 20210204. doi: 10.3389/fphys.2021.628863. PubMed PMID: 33613323; PubMed Central PMCID: PMCPMC7890126.

81. Russo I, Della Gatta PA, Garnham A, Porter J, Burke LM, Costa RJS. Does the nutritional composition of dairy milk based recovery beverages influence post-exercise gastrointestinal and immune status, and subsequent markers of recovery optimisation in response to high intensity interval exercise? Front Nutr. 2020;7:622270. Epub 20210114. doi: 10.3389/fnut.2020.622270. PubMed PMID: 33521041; PubMed Central PMCID: PMCPMC7840831.

82. Russo I, Della Gatta PA, Garnham A, Porter J, Burke LM, Costa RJS. The effects of an acute "train-low" nutritional protocol on markers of recovery optimization in endurance-trained male athletes. Int J Sports Physiol Perform. 2021;16(12):1764-76. Epub 20210527. doi: 10.1123/ijspp.2020-0847. PubMed PMID: 34044369.
