# Supplementary material for: Sports Dietitians Australia and Ultra Sports Science Foundation Joint Position Statement: A Practitioner Guide to the Prevention and Management of Exercise-Associated Gastrointestinal Perturbations and Symptoms
Source: Sports Med. 2025 Apr 7;55(5):1097–134. doi: 10.1007/s40279-025-02186-6 (PMC12106582; doi:10.1007/s40279-025-02186-6)
Supplement: Supplementary file 2 — Supplementary file1 (PDF 320 KB) [file 40279_2025_2186_MOESM2_ESM.pdf]

Sports Medicine

Supplementary File 2

Sports Dietitians Australia and Ultra Sports Science Foundation Joint Position Statement: A practitioner guide to the prevention and management of exercise-associated gastrointestinal perturbations and symptoms.

Ricardo J.S. Costa[1], Stephanie Gaskell[1], Kayla Henningsen[1], Nikki Jeacocke[2], Isabel Martinez[1], Alice Mika[1], Volker Scheer[3], Rachel Scrivin[4,5], Rhiannon Snipe[6], Alice Wallett[2], Pascale Young[1].

[1] Department of Nutrition Dietetics & Food, Monash University, Notting Hill, Victoria, Australia; [2] AIS Performance, Bruce, Canberra, Australia; [3] Ultra Sports Science Foundation, Pierre-Benite, France; [4] University of the Sunshine Coast, Sippy Downs, Queensland, Australia; [5] Toi Ohomai Institute of Technology, Tauranga, New Zealand; [6] School of Exercise and Nutrition Sciences, Deakin University, Burwood, Victoria, Australia.

Other strategies

Pharmaceutical administration

Athletes commonly use pharmaceutical agents in an attempt to mitigate Ex-GIS, especially in the endurance and ultra-endurance competitive scene.[1] Considering the plethora of pharmaceutical agents currently used in the management of gastrointestinal inflammatory and functional diseases/disorders in the clinical setting, with similar pathophysiological alterations as EIGS, it is not surprising that some pharmaceutical agents (i.e., antiemetics and antacids) have been explored for their potential to mitigate EIGS and Ex-GIS. The antiemetic Ondansetron, a central and peripheral serotonin 5-HT3 receptor antagonist, is commonly used as a treatment for nausea and vomiting in clinical practice.[2, 3] Anecdotally, its administration (e.g., 4 mg sublingual dissolvable tables) during ultramarathon running events has been reported to improve nausea and vomiting incidence and severity in event participants.[4] In a recent field-based study, ultramarathon runners, who reported previous history of nausea and vomiting during running, competing in either a 55 km, 80 km, or 160 km trail running event, and were randomly assigned to a 4 mg Ondansetron or placebo capsule intervention (plus, additional doses as required during the event).[5] No symptomatic improvement difference was observed between Ondansetron intervention and placebo. It is however, important to note the lack of Ex-GIS confounder control synonymous with field-based experimental designs.[6] The fact that the intervention only started with symptom onset, indicating a compromised gastrointestinal tract, questions the standardisation of pharmacokinetics and/or pharmacodynamics of the prescribed pharmaceutical agent. Therefore, without laboratory-controlled trials and adequate control of Ex-GIS confounding factors, the potential beneficial effect of Ondansetron is still unknown. Moreover, such pharmaceutical antiemetic activity can only act as an acute ‘band-aid’ to ‘mask’ symptoms, and does not resolve the underlying causal mechanisms of the nausea and/or vomiting.

Prophylactic gastric acid suppressant administration (i.e., antacids: H_2 receptor blockers/antagonists and proton-pump inhibitors), and its clinical role in reducing ulceration (i.e., mucosal and epithelial erosion injury) along parts of the gastrointestinal tract,[7, 8] may theoretically provide a protective effect in athletes exposed to circulatory-gastrointestinal pathophysiology of EIGS (i.e., gastric acid secretion and exposure within a scenario of compromised gastrointestinal epithelial tissue). An ultra-endurance focused field-study investigated the effect of H_2 receptor antagonist Cimetidine administration (800 mg) 1 h before and mid-point during a 100-mile running competition on Ex-GIS and faecal blood presence (i.e., indicative of mucosal and epithelial tissue erosion injury).[9] Ultramarathon runners allocated to Cimetidine administration presented lower nausea and vomiting, and faecal blood presence, compared with runners allocated to placebo.[9] Conversely, a follow-up marathon competition field-study reported no difference in Ex-GIS and faecal blood presence in runners allocated to 800 mg Cimetidine administration 2 h before a competition, compared with runners allocated to placebo.[10] In respect to proton pump inhibitors, runners that received a daily dose of Pantoprazole (20 mg/day) during the Spartathlon ultramarathon (246 km) presented lower incidence of faecal blood presence in response to the ultramarathon participation, compared with the placebo group.[11] Despite the scarcity of proton pump inhibitor research in managing gastrointestinal issues on athlete populations, administration has been used effectively in several animal-athlete (i.e., sled dog racing and equine sports) studies, by which Omeprazole administration interventions have shown reduce exercise-associated gastritis, gastrointestinal ulceration, and/or aligned faecal blood presence.[12-16] It is not withstanding, that normal stomach acid secretions and suppressing standard biological function may lead to an imbalance in gastrointestinal function during an already compromised gastrointestinal state;[17-19] plus, an array of side effects with antacid administration may outweigh any benefits for individuals exposure to EIGS and Ex-GIS.[7] Despite H_2 receptor antagonist and proton-pump inhibitor focused studies presenting some potentially beneficial effects on Ex-GIS and/or mucosal/epithelial erosion, these field-based studies are synonymous with methodological concerns in regards to current knowledge of confounder control,[6]

and such pharmaceutical antacid activity does not resolve the underlying causal factors of the reported nausea, vomiting, and faecal blood presence.

Grade of evidence: IV-V

Physical manoeuvres

One study to date has investigated the impact of physical manoeuvres on Ex-GIS. Seven male college graduates who reported suffering frequently from exercise-related transient abdominal pain for 15 min at an estimated maximal intensity after consuming 14 mL/kg decarbonated coca cola.[20] Contraction of the abdominal muscles, modified breathing, and tightening of an abdominal belt for 30 sec were effective physical manoeuvres that significantly decreased the intensity of exercise-related transient abdominal pain for 30-60 sec after the manoeuvre. Findings from this study are limited due to the inability to blind athletes to the physical manoeuvres, small number of athletes, and the short duration of the reduction in exercise-related transient abdominal pain.

Grade of evidence: IV

Compression socks

One study to date has investigated the impact of compression sock use during a marathon competition on markers of EIGS.[21] Forty-six men and women marathon entrants were recruited and allocated to either control or compression sock groups. Participants within the sock group were required to wear a pair of provided compression socks with an estimated pressure of 25 mmHg exerted at the ankle, prior to, during, and until after the post marathon venous blood draw. While the study reported a significant reduction in plasma I-FABP concentration in the sock group compared to control, it is worth noting that food (e.g., carbohydrates) and fluid (hydration status) intake during the marathon was not controlled, monitored, and/or measured to any rigorous degree during the study (Sections 3, 4 and 7); which are key strategies that have been highlighted to impact splanchnic blood flow, and subsequent EIGS biomarkers, including I-FABP.[22-28] It therefore cannot be concluded that

compression socks are effective in mitigating intestinal injury, as further research is required in laboratory settings with adequate confounder control to assess the effect of compression socks alone.[6]

Grade of evidence: IV

Other considerations

- Feeding tolerance: Section 4 highlighted the protective role of macronutrient pre- and during-exercise on gastrointestinal integrity and systemic markers, especially carbohydrate provisions. However, such nutritional intake pre- and during-exercise are notorious for promoting Ex-GIS,[23, 28] due to feeding intolerance of various aspects of the provisions (e.g., volume, concentration, dose and frequency, texture, type, and/or inclusion of other caloric or non-caloric nutrition), which is seen to be highly individualised.[29] Supplementary file 1 discusses the impact of various aspects of feeding tolerance on Ex-GIS.

- Sodium intake: Section 7 has discussed evidence suggesting that starting exercise euhydrated and maintaining euhydration within feeding tolerance levels may lower the risk of EIGS and Ex-GIS. Therefore, increasing daily dietary sodium chloride intake, and subsequent promotion towards increasing retention of ingested water, may enhance body water content and provide a pre-exercise hyperhydration status.[30] Moreover, sodium chloride ingestion during submaximal exercise increases thirst and *ad libitum* fluid intake,[31] which could result in attenuating the fall in plasma volume over multiple hours of exercise, better contributing to euhydration maintenance. However, the focused euhydration efforts of sodium do not translate into exerting any effect on core body temperature, a key EIGS external exacerbation factor, independent of water intake.[32, 33] Despite plausibility, the increase in dietary sodium chloride intake prior to a prolonged bout of exercise and/or sodium chloride intake during endurance exercise does not exert any substantial influence on EIGS outcomes and/or incidence or severity of Ex-GIS.[6, 30, 34, 35] Moreover, from a GIS perspective,

a recent systematic literature review focusing on hyperhydration interventions,[36] reported no additional GIS (e.g., abdominal bloating or nausea) instigation with sodium chloride supplementation versus no treatment,[37] did not increase GIS compared with glycerol supplementation intervention,[38-40] and GIS reporting did not differ between sodium supplementation form (i.e., solution versus dissolvable tablets) and type (i.e., sodium bicarbonate and sodium citrate),[39, 41] within tolerable doses. However, prevalence of diarrhoea increased with sodium chloride dosage increase,[42] and sodium bicarbonate supplementation applied as an acidity buffer for short duration high intensity anaerobic exercise bouts is synonymous with GIS that may impact performance outcomes.[43, 44]

- Glycerol: Glycerol supplementation interventions in the attempt to increase water retention to aid hyperhydration may lead to greater Ex-GIS. [45, 46] A recent systematic literature review on this topic,[36] reported GIS (i.e., abdominal bloating, diarrhoea, vomiting, and/or nausea) during and following hyperhydration interventions using glycerol supplementation, with some reports of withdrawal from participation due to GIS.[47-51] It is important to note that other studies reported no GIS as a result of glycerol intervention.[36] Discrepancy may be due methodological differences in experimental design, including to the wide array of subjective GIS assessment tools employed, which included tools with and without validation and reliability tested, and/or participant education of tool.[6]

- Medium chain triglycerides (MCT): Studies that have investigated Ex-GIS with MCT supplementation, with or without carbohydrate in adjunct, prior to and during prolonged endurance-based exercise protocols (e.g., 2-3 h 55-60% W_{max} or $\dot{V}O_{2max}$, plus time trial) have predominantly reported exacerbated GIS (i.e., belching, abdominal bloating and cramping, gastric reflux, and nausea) with MCT inclusion compared with the respective placebo or control.[52-55] The link between MCT and Ex-GIS may likely be associated with some form of gastric, intestinal, and/or

accessory organ burden induced by the MCT dose alongside the exercise-associated compromise of the gastrointestinal tract. Therefore, consensus for practical application would be to avoid MCT around exercise to decrease the risk of Ex-GIS.

Recovery nutrition considerations

As a result of the circulatory- and neuroendocrine-gastrointestinal pathway disturbances, and/or mechanical strain aspects, of EIGS, alongside feeding tolerance issues (i.e., intake disinterest linked to low appetite), it is likely that the digestive and absorptive functions of the gastrointestinal tract will be compromised in the post-exercise period. At the recovery stage of exercise activities, the application of recovery nutrition (e.g., carbohydrate, protein, and water) is imperative to aid fuel replacement (i.e., muscle glycogen resynthesis), tissue repair and adaptations (i.e., protein muscle synthesis), and rehydration (i.e., return to euhydration).[58] In addition, post-exercise recovery nutrition appears important for the restoration or maintenance of immune functional responses (e.g., avoidance of immunodepression) linked with tissue debris and pathogenic clearance, namely, neutrophil degranulation and oral-mucosal anti-microbial agents, consistently observed to be altered after exercise, compared with pre-exercise resting homeostasis.[24, 59-62]

Considering the compromised gastrointestinal tract as a result of exercise stress, a set of recent recovery optimisation exploratory studies showed that the provisions of recovery nutrition via a variety of recovery beverages trials (i.e., standard carbohydrate-electrolyte sports drink, chocolate flavoured dairy milk beverage, low carbohydrate and higher protein chocolate flavoured dairy milk beverage, and dairy based powder supplement beverage) results in carbohydrate malabsorption of all tested beverages, with the magnitude of malabsorption dependant on the amount of carbohydrates provided (Supplementary file 2- Figure 1).[63-65] These outcomes translated into symptom manifestations with high nutritional provision and malabsorption resulting in proportionally greater gut discomfort and total-GIS, as a result of greater lower-GIS (i.e., lower abdominal bloating and

pain, urge to defecate and abnormal defecation including watery stools and diarrhoea) (Supplementary file 2- Figure 2). From a practical translational perspective, to optimise the bioavailability of recovery nutrition, and avoid post-exercise GIS, linked to the overwhelming of the gastrointestinal tract during a period of compromised status, it is proposed that the consumption of the generalised recovery nutrition guidelines and/or recommendation (e.g., 1.2 g/kg carbohydrate, 0.4 g/kg protein, and x1.5 body water losses, starting immediately upon exercise cessation and along the 2 h post-exercise recovery period); but with the added care of '*small and frequent*' intake along the exercise recovery period, within an individual's identified and established tolerance (i.e., via gastrointestinal assessment- Supplementary file 3, clinical practice: supporting the individual athlete).[17, 18, 57, 58, 66, 67] For example, even volume doses every 15 min over the 2 h post-exercise recovery period. Any nutritional addition above such recommendations is likely to be malabsorbed (i.e., loss of bioavailability), and increase the risk of GIS, which will further compromise subsequent longer-term recovery nutrition optimisation through disturbing the individual's routine food and fluid intake.[68] However, it is important to highlight that the exact prescriptive dosage and timing of '*small and frequent*' intake over the 2 h acute recovery period still warrants further investigation.

Supplementary file 2- Figure 1. Carbohydrate malabsorption in response to the post-exercise intake of isovolumetric recovery beverages of differing nutritional compositions. Reconstituted dairy powder supplement beverage (black squares: 2.2 g/kg body mass (BM) carbohydrate and 0.8 g/kgBM protein), dairy milk beverage (black circles: 1.2 g/kgBM carbohydrate and 0.4 g/kgBM protein), carbohydrate electrolyte beverage (white squares: 0.76 g/kgBM and 0 g/kgBM protein), and low carbohydrate dairy milk beverage (white circles: 0.35 g/kgBM carbohydrate and 0.5 g/kgBM protein).[63-65]

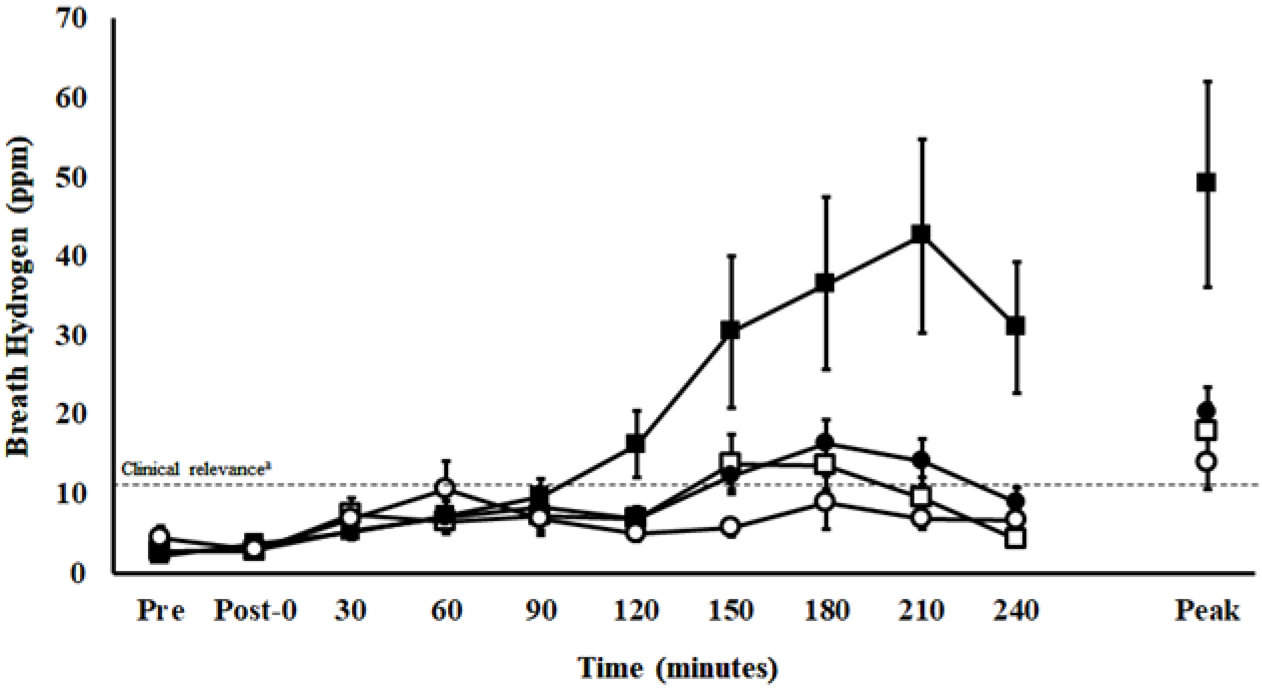

Supplementary file 2- Figure 2. Gut discomfort, total-, upper-, and lower-gastrointestinal symptoms (GIS) in response to the post-exercise intake of isovolumetric recovery beverages of differing nutritional compositions. Reconstituted dairy powder supplement beverage (RDPS: 2.2 g/kg body mass (BM) carbohydrate and 0.8 g/kgBM protein), dairy milk beverage (DM: 1.2 g/kgBM carbohydrate and 0.4 g/kgBM protein), carbohydrate electrolyte beverage (CEB: 0.76 g/kgBM and 0.0 g/kgBM protein), and low carbohydrate dairy milk beverage (LCDM: 0.35 g/kgBM carbohydrate and 0.5 g/kgBM protein).[63-65]

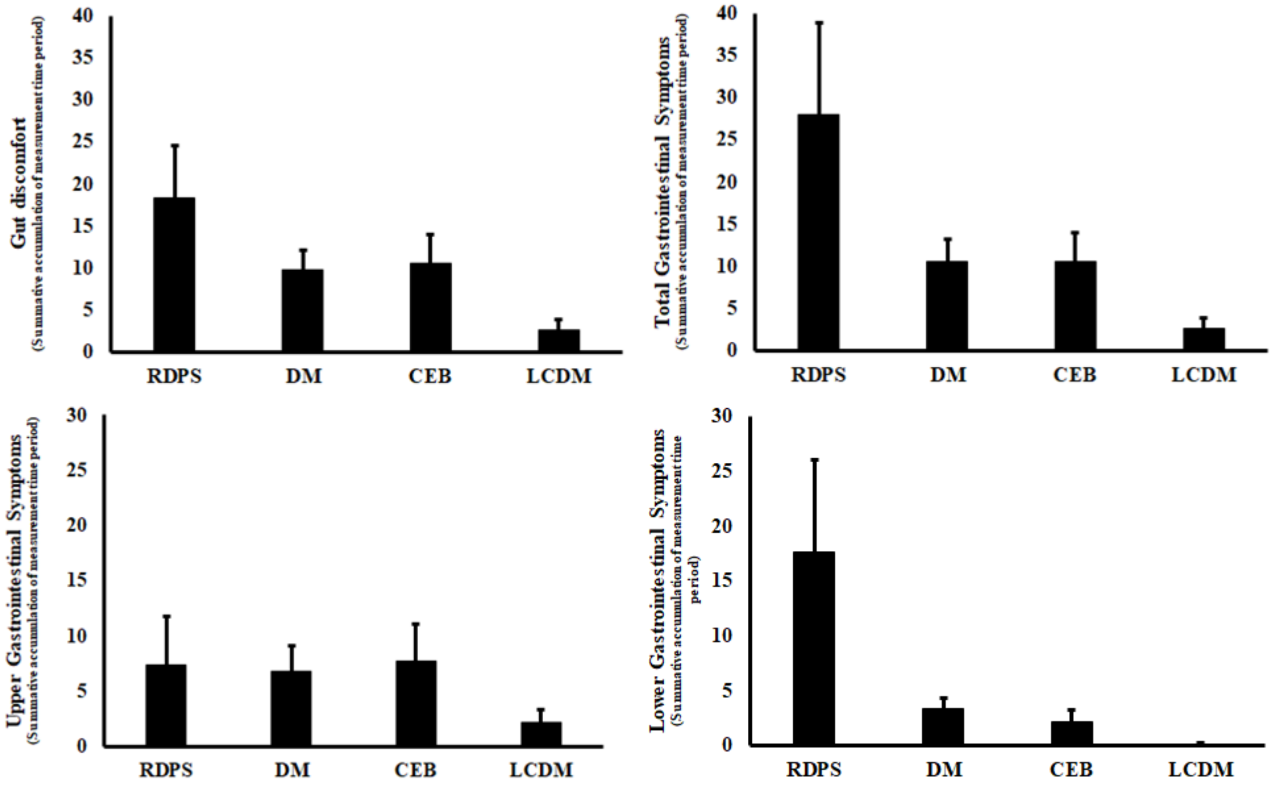

References

1. Scrivin R, Costa RJS, Pelly F, Lis D, Slater G. An exploratory study of the management strategies reported by endurance athletes with exercise-associated gastrointestinal symptoms. Front Nutr. 2022;9:1003445. Epub 20221109. doi: 10.3389/fnut.2022.1003445. PubMed PMID: 36438762; PubMed Central PMCID: PMCPMC9691682.

2. Canziani BC, Uestuener P, Fossali EF, Lava SAG, Bianchetti MG, Agostoni C, et al. Clinical practice: Nausea and vomiting in acute gastroenteritis: physiopathology and management. Eur J Pediatr. 2018;177(1):1-5. Epub 20170930. doi: 10.1007/s00431-017-3006-9. PubMed PMID: 28963679.

3. Golembiewski J, Chernin E, Chopra T. Prevention and treatment of postoperative nausea and vomiting. Am J Health Syst Pharm. 2005;62(12):1247-60; quiz 61-2. doi: 10.1093/ajhp/62.12.1247. PubMed PMID: 15947124.

4. Pasternak A. Use of ondansetron for nausea and vomiting during an ultra-endurance run. International Journal of Sports Physiology and Performance. 2018;13:6.

5. Pasternak AV, Fiore D, Islas A, Toti S, Hoffman MD. Treatment with oral ondansetron for ultramarathon-associated nausea: The TOO FUN study. Sports (Basel). 2021;9(3). Epub 20210303. doi: 10.3390/sports9030035. PubMed PMID: 33802399; PubMed Central PMCID: PMCPMC8001581.

6. Costa RJS, Young P, Gill SK, Snipe RMJ, Gaskell S, Russo I, et al. Assessment of exercise-associated gastrointestinal perturbations in research and practical settings: methodological concerns and recommendations for best practice. Int J Sport Nutr Exerc Metab. 2022;32(5):387-418. Epub 20220813. doi: 10.1123/ijsnem.2022-0048. PubMed PMID: 35963615.

7. Abraham NS. Proton pump inhibitors: potential adverse effects. Curr Opin Gastroenterol. 2012;28(6):615-20. doi: 10.1097/MOG.0b013e328358d5b9. PubMed PMID: 23010681.

8. Moses FM. Gastrointestinal bleeding and the athlete. Am J Gastroenterol. 1993;88(8):1157-9. PubMed PMID: 8338081.

9. Baska RS, Moses FM, Deuster PA. Cimetidine reduces running-associated gastrointestinal bleeding. A prospective observation. Dig Dis Sci. 1990;35(8):956-60. doi: 10.1007/bf01537243. PubMed PMID: 2384041.

10. Moses FM, Baska RS, Peura DA, Deuster PA. Effect of cimetidine on marathon-associated gastrointestinal symptoms and bleeding. Dig Dis Sci. 1991;36(10):1390-4. doi: 10.1007/bf01296804. PubMed PMID: 1914760.

11. Thalmann M, Sodeck GH, Kavouras S, Matalas A, Skenderi K, Yannikouris N, et al. Proton pump inhibition prevents gastrointestinal bleeding in ultramarathon runners: a randomised, double blinded, placebo controlled study. Br J Sports Med. 2006;40(4):359-62; discussion 62. doi: 10.1136/bjsm.2005.024463. PubMed PMID: 16556794; PubMed Central PMCID: PMCPMC2577540.

12. Davis MS, Willard MD, Nelson SL, McCullough SM, Mandsager RE, Roberts J, et al. Efficacy of omeprazole for the prevention of exercise-induced gastritis in racing Alaskan sled dogs. J Vet Intern Med. 2003;17(2):163-6. doi: 10.1111/j.1939-1676.2003.tb02428.x. PubMed PMID: 12683615.

13. Mabry K, Davis MS, Gould E, Gogal RM, Steiner JM, Tolbert MK, et al. Assessment of gastrointestinal health in racing Alaskan sled dogs using capsule endoscopy and inflammatory cytokines. J Small Anim Pract. 2023;64(9):574-80. Epub 20230521. doi: 10.1111/jsap.13627. PubMed PMID: 37211353.

14. Ricord M, Andrews FM, Yñiguez FJM, Keowen M, Garza F, Jr., Paul L, et al. Impact of concurrent treatment with omeprazole on phenylbutazone-induced equine gastric ulcer syndrome (EGUS). Equine Vet J. 2021;53(2):356-63. Epub 20200818. doi: 10.1111/evj.13323. PubMed PMID: 32697849.

15. Williamson KK, Willard MD, McKenzie EC, Royer CM, Payton ME, Davis MS. Efficacy of famotidine for the prevention of exercise-induced gastritis in racing Alaskan sled dogs. J Vet Intern Med. 2007;21(5):924-7. doi: 10.1892/0891-6640(2007)21[924:eofftp]2.0.co;2. PubMed PMID: 17939544.

16. Williamson KK, Willard MD, Payton ME, Davis MS. Efficacy of omeprazole versus high-dose famotidine for prevention of exercise-induced gastritis in racing Alaskan sled dogs. J Vet Intern Med. 2010;24(2):285-8. doi: 10.1111/j.1939-1676.2009.0454.x. PubMed PMID: 20102495.

17. Gaskell SK, Burgell R, Wiklendt L, Dinning P, Costa RJS. Does exertional heat stress impact gastrointestinal function and symptoms? J Sci Med Sport. 2022;25(12):960-7. Epub 20221017. doi: 10.1016/j.jsams.2022.10.008. PubMed PMID: 36347748.

18. Gaskell SK, Burgell R, Wiklendt L, Dinning PG, Costa RJS. Impact of exercise duration on gastrointestinal function and symptoms. J Appl Physiol (1985). 2023;134(1):160-71. Epub 20221208. doi: 10.1152/japplphysiol.00393.2022. PubMed PMID: 36476157.

19. Horner KM, Schubert MM, Desbrow B, Byrne NM, King NA. Acute exercise and gastric emptying: a meta-analysis and implications for appetite control. Sports Med. 2015;45(5):659-78. doi: 10.1007/s40279-014-0285-4. PubMed PMID: 25398225.

20. Plunkett BT, Hopkins WG. Investigation of the side pain "stitch" induced by running after fluid ingestion. Med Sci Sports Exerc. 1999;31(8):1169-75. doi: 10.1097/00005768-199908000-00014. PubMed PMID: 10449020.

21. Zadow EK, Edwards KH, Kitic CM, Fell JW, Adams MJ, Singh I, et al. Compression socks reduce running-induced intestinal damage. J Strength Cond Res. 2022;36(9):2461-4. Epub 20201015. doi: 10.1519/jsc.0000000000003870. PubMed PMID: 33065702.

22. Alcock R, McCubbin A, Camões-Costa V, Costa RJS. Case study: Providing nutritional support to an ultraendurance runner in preparation for a self-sufficient multistage ultramarathon:

rationed versus full energy provisions. Wilderness Environ Med. 2018;29(4):508-20. Epub 20180922. doi: 10.1016/j.wem.2018.06.004. PubMed PMID: 30249353.

23. Costa RJS, Miall A, Khoo A, Rauch C, Snipe R, Camões-Costa V, et al. Gut-training: the impact of two weeks repetitive gut-challenge during exercise on gastrointestinal status, glucose availability, fuel kinetics, and running performance. Appl Physiol Nutr Metab. 2017;42(5):547-57. Epub 20170322. doi: 10.1139/apnm-2016-0453. PubMed PMID: 28177715.

24. Costa RJS, Camões-Costa V, Snipe RMJ, Dixon D, Russo I, Huschtscha Z. Impact of exercise-induced hypohydration on gastrointestinal integrity, function, symptoms, and systemic endotoxin and inflammatory profile. J Appl Physiol (1985). 2019;126(5):1281-91. Epub 20190321. doi: 10.1152/japplphysiol.01032.2018. PubMed PMID: 30896356.

25. Flood TR, Montanari S, Wicks M, Blanchard J, Sharp H, Taylor L, et al. Addition of pectin-alginate to a carbohydrate beverage does not maintain gastrointestinal barrier function during exercise in hot-humid conditions better than carbohydrate ingestion alone. Appl Physiol Nutr Metab. 2020;45(10):1145-55. Epub 20200504. doi: 10.1139/apnm-2020-0118. PubMed PMID: 32365303.

26. Jonvik KL, Lenaerts K, Smeets JSJ, Kolkman JJ, LJC VANL, Verdijk LB. Sucrose but not nitrate ingestion reduces strenuous cycling-induced intestinal injury. Med Sci Sports Exerc. 2019;51(3):436-44. doi: 10.1249/mss.0000000000001800. PubMed PMID: 30299412.

27. Rehrer NJ, Goes E, DuGardeyn C, Reynaert H, DeMeirleir K. Effect of carbohydrate on portal vein blood flow during exercise. Int J Sports Med. 2005;26(3):171-6. doi: 10.1055/s-2004-820957. PubMed PMID: 15776331.

28. Snipe RMJ, Khoo A, Kitic CM, Gibson PR, Costa RJS. Carbohydrate and protein intake during exertional heat stress ameliorates intestinal epithelial injury and small intestine permeability. Appl Physiol Nutr Metab. 2017;42(12):1283-92. Epub 20170804. doi: 10.1139/apnm-2017-0361. PubMed PMID: 28777927.

29. Rauch CE, McCubbin AJ, Gaskell SK, Costa RJS. Feeding tolerance, glucose availability, and whole-body total carbohydrate and fat oxidation in male endurance and ultra-endurance runners in response to prolonged exercise, consuming a habitual mixed macronutrient diet and carbohydrate feeding during exercise. Front Physiol. 2021;12:773054. Epub 20220104. doi: 10.3389/fphys.2021.773054. PubMed PMID: 35058795; PubMed Central PMCID: PMCPMC8764139.

30. McCubbin AJ, Lopez MB, Cox GR, Caldwell Odgers JN, Costa RJS. Impact of 3-day high and low dietary sodium intake on sodium status in response to exertional-heat stress: a double-blind randomized control trial. Eur J Appl Physiol. 2019;119(9):2105-18. Epub 20190803. doi: 10.1007/s00421-019-04199-2. PubMed PMID: 31377851.

31. Del Coso J, González-Millán C, Salinero JJ, Abián-Vicén J, Areces F, Lledó M, et al. Effects of oral salt supplementation on physical performance during a half-ironman: A randomized controlled trial. Scand J Med Sci Sports. 2016;26(2):156-64. Epub 20150214. doi: 10.1111/sms.12427. PubMed PMID: 25683094.

32. Earhart EL, Weiss EP, Rahman R, Kelly PV. Effects of oral sodium supplementation on indices of thermoregulation in trained, endurance athletes. J Sports Sci Med. 2015;14(1):172-8. Epub 20150301. PubMed PMID: 25729305; PubMed Central PMCID: PMCPMC4306770.

33. Henningsen K, Mika A, Alcock R, Gaskell SK, Parr A, Rauch C, et al. The increase in core body temperature in response to exertional-heat stress can predict exercise-induced gastrointestinal syndrome. Temperature. 2023:1-20. doi: 10.1080/23328940.2023.2213625.

34. McCubbin AJ, da Costa RJS. Effect of personalized sodium replacement on fluid and sodium balance and thermophysiological strain during and after ultraendurance running in the heat. Int J Sports Physiol Perform. 2024;19(2):105-15. Epub 20231109. doi: 10.1123/ijspp.2023-0295. PubMed PMID: 37944507.

35. Costa RJS, Mika AS, McCubbin AJ. The impact of exercise modality on exercise-induced gastrointestinal syndrome and associated gastrointestinal symptoms. J Sci Med Sport. 2022;25(10):788-93. Epub 20220712. doi: 10.1016/j.jsams.2022.07.003. PubMed PMID: 35868987.

36. Jardine WT, Aisbett B, Kelly MK, Burke LM, Ross ML, Condo D, et al. The effect of pre-exercise hyperhydration on exercise performance, physiological outcomes and gastrointestinal symptoms: A systematic review. Sports Med. 2023;53(11):2111-34. Epub 20230725. doi: 10.1007/s40279-023-01885-2. PubMed PMID: 37490269; PubMed Central PMCID: PMCPMC10587316.

37. Gigou PY, Dion T, Asselin A, Berrigan F, Goulet ED. Pre-exercise hyperhydration-induced bodyweight gain does not alter prolonged treadmill running time-trial performance in warm ambient conditions. Nutrients. 2012;4(8):949-66. Epub 20120813. doi: 10.3390/nu4080949. PubMed PMID: 23016126; PubMed Central PMCID: PMCPMC3448081.

38. Goulet EDB, De La Flore A, Savoie FA, Gosselin J. Salt + glycerol-induced hyperhydration enhances fluid retention more than salt- or glycerol-induced hyperhydration. Int J Sport Nutr Exerc Metab. 2018;28(3):246-52. Epub 20180517. doi: 10.1123/ijsnem.2017-0310. PubMed PMID: 29140136.

39. Savoie FA, Asselin A, Goulet ED. Comparison of sodium chloride tablets-induced, sodium chloride solution-induced, and glycerol-induced hyperhydration on fluid balance responses in healthy men. J Strength Cond Res. 2016;30(10):2880-91. doi: 10.1519/jsc.0000000000001371. PubMed PMID: 26849790.

40. Savoie FA, Dion T, Asselin A, Goulet ED. Sodium-induced hyperhydration decreases urine output and improves fluid balance compared with glycerol- and water-induced hyperhydration. Appl Physiol Nutr Metab. 2015;40(1):51-8. doi: 10.1139/apnm-2014-0243. PubMed PMID: 25494972.

41. Siegler JC, Carr AJ, Jardine WT, Convit L, Cross R, Chapman D, et al. The hyperhydration potential of sodium bicarbonate and sodium citrate. Int J Sport Nutr Exerc Metab. 2022;32(2):74-81. Epub 20211207. doi: 10.1123/ijsnem.2021-0179. PubMed PMID: 34875625.

42. Sugihara A, Fujii N, Tsuji B, Watanabe K, Niwa T, Nishiyasu T. Hypervolemia induced by fluid ingestion at rest: effect of sodium concentration. Eur J Appl Physiol. 2014;114(10):2139-45. Epub 20140625. doi: 10.1007/s00421-014-2933-7. PubMed PMID: 24962003.

43. Kahle LE, Kelly PV, Eliot KA, Weiss EP. Acute sodium bicarbonate loading has negligible effects on resting and exercise blood pressure but causes gastrointestinal distress. Nutr Res. 2013;33(6):479-86. Epub 20130517. doi: 10.1016/j.nutres.2013.04.009. PubMed PMID: 23746564; PubMed Central PMCID: PMCPMC3680785.

44. Lancha Junior AH, Painelli Vde S, Saunders B, Artioli GG. Nutritional strategies to modulate intracellular and extracellular buffering capacity during high-intensity exercise. Sports Med. 2015;45 Suppl 1:S71-81. doi: 10.1007/s40279-015-0397-5. PubMed PMID: 26553493; PubMed Central PMCID: PMCPMC4672007.

45. Goulet ED, Aubertin-Leheudre M, Plante GE, Dionne IJ. A meta-analysis of the effects of glycerol-induced hyperhydration on fluid retention and endurance performance. Int J Sport Nutr Exerc Metab. 2007;17(4):391-410. doi: 10.1123/ijsnem.17.4.391. PubMed PMID: 17962713.

46. Murray R, Eddy DE, Paul GL, Seifert JG, Halaby GA. Physiological responses to glycerol ingestion during exercise. J Appl Physiol (1985). 1991;71(1):144-9. doi: 10.1152/jappl.1991.71.1.144. PubMed PMID: 1917736.

47. Coutts A, Reaburn P, Mummery K, Holmes M. The effect of glycerol hyperhydration on olympic distance triathlon performance in high ambient temperatures. Int J Sport Nutr Exerc Metab. 2002;12(1):105-19. doi: 10.1123/ijsnem.12.1.105. PubMed PMID: 11993618.

48. Dini M, Corbianco S, Rossi B, Lucacchini A. Hyperhydrating with glycerol: Effects on thermoregulation, hydration and athletic performance during specific exergonic exercise in a warm-humid environment. Sport Sciences for Health. 2007;2(1):1-7. doi: 10.1007/s11332-007-0031-5.

49. Latzka WA, Sawka MN, Montain SJ, Skrinar GS, Fielding RA, Matott RP, et al. Hyperhydration: Thermoregulatory effects during compensable exercise-heat stress. J Appl Physiol (1985). 1997;83(3):860-6. doi: 10.1152/jappl.1997.83.3.860. PubMed PMID: 9292474.

50. Lyons TP, Riedesel ML, Meuli LE, Chick TW. Effects of glycerol-induced hyperhydration prior to exercise in the heat on sweating and core temperature. Med Sci Sports Exerc. 1990;22(4):477-83. PubMed PMID: 2402207.

51. Polyviou TP, Pitsiladis YP, Lee WC, Pantazis T, Hambly C, Speakman JR, et al. Thermoregulatory and cardiovascular responses to creatine, glycerol and alpha lipoic acid in trained cyclists. J Int Soc Sports Nutr. 2012;9(1):29. Epub 20120622. doi: 10.1186/1550-2783-9-29. PubMed PMID: 22726625; PubMed Central PMCID: PMCPMC3459729.

52. Goedecke JH, Elmer-English R, Dennis SC, Schloss I, Noakes TD, Lambert EV. Effects of medium-chain triaclyglycerol ingested with carbohydrate on metabolism and exercise performance. Int J Sport Nutr. 1999;9(1):35-47. doi: 10.1123/ijsn.9.1.35. PubMed PMID: 10036340.

53. Jeukendrup AE, Thielen JJ, Wagenmakers AJ, Brouns F, Saris WH. Effect of medium-chain triacylglycerol and carbohydrate ingestion during exercise on substrate utilization and subsequent cycling performance. Am J Clin Nutr. 1998;67(3):397-404. doi: 10.1093/ajcn/67.3.397. PubMed PMID: 9497182.

54. Thorburn MS, Vistisen B, Thorp RM, Rockell MJ, Jeukendrup AE, Xu X, et al. Attenuated gastric distress but no benefit to performance with adaptation to octanoate-rich esterified oils in well-trained male cyclists. J Appl Physiol (1985). 2006;101(6):1733-43. Epub 20060713. doi: 10.1152/japplphysiol.00393.2006. PubMed PMID: 16840580.

55. Vistisen B, Nybo L, Xu X, Høy CE, Kiens B. Minor amounts of plasma medium-chain fatty acids and no improved time trial performance after consuming lipids. J Appl Physiol (1985). 2003;95(6):2434-43. Epub 20030815. doi: 10.1152/japplphysiol.00118.2003. PubMed PMID: 12923114.

56. Rehrer NJ, van Kemenade M, Meester W, Brouns F, Saris WH. Gastrointestinal complaints in relation to dietary intake in triathletes. Int J Sport Nutr. 1992;2(1):48-59. doi: 10.1123/ijsn.2.1.48. PubMed PMID: 1338583.

57. Gaskell SK, Rauch CE, Costa RJS. Gastrointestinal assessment and therapeutic intervention for the management of exercise-associated gastrointestinal symptoms: A case series translational and professional practice approach. Front Physiol. 2021;12:719142. Epub 20210907. doi: 10.3389/fphys.2021.719142. PubMed PMID: 34557109; PubMed Central PMCID: PMCPMC8452991.

58. Russo I, Camões-Costa, V., Gaskell, S.K., Porter, J., Burke, L.M., Costa, R.J.S. Systematic literature review: The effect of dairy milkon markers of recovery optimisation in response to endurance exercise. International Journal of Sports Science. 2019;9(4):69-85.

59. Costa RJ, Oliver SJ, Laing SJ, Waiters R, Bilzon JL, Walsh NP. Influence of timing of postexercise carbohydrate-protein ingestion on selected immune indices. Int J Sport Nutr Exerc Metab. 2009;19(4):366-84. doi: 10.1123/ijsnem.19.4.366. PubMed PMID: 19827462.

60. Costa RJ, Walters R, Bilzon JL, Walsh NP. Effects of immediate postexercise carbohydrate ingestion with and without protein on neutrophil degranulation. Int J Sport Nutr Exerc Metab. 2011;21(3):205-13. doi: 10.1123/ijsnem.21.3.205. PubMed PMID: 21719901.

61. Costa RJ, Fortes MB, Richardson K, Bilzon JL, Walsh NP. The effects of postexercise feeding on saliva antimicrobial proteins. Int J Sport Nutr Exerc Metab. 2012;22(3):184-91. doi: 10.1123/ijsnem.22.3.184. PubMed PMID: 22693239.

62. Costa RJS, Camões-Costa V, Snipe RMJ, Dixon D, Russo I, Huschtscha Z. The impact of a dairy milk recovery beverage on bacterially stimulated neutrophil function and gastrointestinal tolerance in response to hypohydration inducing exercise stress. Int J Sport Nutr Exerc Metab. 2020;30(4):237-48. Epub 20200526. doi: 10.1123/ijsnem.2019-0349. PubMed PMID: 32460239.

63. Russo I, Della Gatta PA, Garnham A, Porter J, Burke LM, Costa RJS. Does the nutritional composition of dairy milk based recovery beverages influence post-exercise gastrointestinal and immune status, and subsequent markers of recovery optimisation in response to high intensity interval exercise? Front Nutr. 2020;7:622270. Epub 20210114. doi: 10.3389/fnut.2020.622270. PubMed PMID: 33521041; PubMed Central PMCID: PMCPMC7840831.

64. Russo I, Della Gatta PA, Garnham A, Porter J, Burke LM, Costa RJS. The effects of an acute "train-low" nutritional protocol on markers of recovery optimization in endurance-trained male athletes. Int J Sports Physiol Perform. 2021;16(12):1764-76. Epub 20210527. doi: 10.1123/ijspp.2020-0847. PubMed PMID: 34044369.

65. Russo I, Della Gatta PA, Garnham A, Porter J, Burke LM, Costa RJS. Assessing overall exercise recovery processes using carbohydrate and carbohydrate-protein containing recovery beverages. Front Physiol. 2021;12:628863. Epub 20210204. doi: 10.3389/fphys.2021.628863. PubMed PMID: 33613323; PubMed Central PMCID: PMCPMC7890126.

66. Burke LM, Castell LM, Casa DJ, Close GL, Costa RJS, Desbrow B, et al. International Association of Athletics Federations consensus statement 2019: Nutrition for athletics. Int J Sport Nutr Exerc Metab. 2019;29(2):73-84. Epub 20190405. doi: 10.1123/ijsnem.2019-0065. PubMed PMID: 30952204.

67. Thomas DT, Erdman KA, Burke LM. American College of Sports Medicine joint position statement. Nutrition and athletic performance. Med Sci Sports Exerc. 2016;48(3):543-68. doi: 10.1249/mss.0000000000000852. PubMed PMID: 26891166.

68. Costa RJ, Snipe R, Camões-Costa V, Scheer V, Murray A. The impact of gastrointestinal symptoms and dermatological injuries on nutritional intake and hydration status during ultramarathon events. Sports Med Open. 2016;2:16. Epub 20160105. doi: 10.1186/s40798-015-0041-9. PubMed PMID: 26767151; PubMed Central PMCID: PMCPMC4701764.
