# Supplementary material for: Sports Dietitians Australia and Ultra Sports Science Foundation Joint Position Statement: A Practitioner Guide to the Prevention and Management of Exercise-Associated Gastrointestinal Perturbations and Symptoms
Source: Sports Med. 2025 Apr 7;55(5):1097–134. doi: 10.1007/s40279-025-02186-6 (PMC12106582; doi:10.1007/s40279-025-02186-6)
Supplement: Supplementary file 3 — Supplementary file2 (PDF 393 KB) [file 40279_2025_2186_MOESM3_ESM.pdf]

Sports Medicine

Supplementary File 3

Sports Dietitians Australia and Ultra Sports Science Foundation Joint Position Statement: A practitioner guide to the prevention and management of exercise-associated gastrointestinal perturbations and symptoms.

Ricardo J.S. Costa[1], Stephanie Gaskell[1], Kayla Henningsen[1], Nikki Jeacocke[2], Isabel Martinez[1], Alice Mika[1], Volker Scheer[3], Rachel Scrivin[4,5], Rhiannon Snipe[6], Alice Wallett[2], Pascale Young[1].

[1] Department of Nutrition Dietetics & Food, Monash University, Notting Hill, Victoria, Australia; [2] AIS Performance, Bruce, Canberra, Australia; [3] Ultra Sports Science Foundation, Pierre-Benite, France; [4] University of the Sunshine Coast, Sippy Downs, Queensland, Australia; [5] Toi Ohomai Institute of Technology, Tauranga, New Zealand; [6] School of Exercise and Nutrition Sciences, Deakin University, Burwood, Victoria, Australia.

Translation to practice

Assessment and intervention procedures for clinical practice- Supporting the individual athlete:

Considering the dynamic and multifactorial nature of EIGS and Ex-GIS,[1] an individualised gastrointestinal exercise assessment to determine the main causal pathway(s) of EIGS and exacerbator factors that are instigating Ex-GIS is recommended. Results from the assessment will then provide information to decide the most appropriate prevention and management strategy of EIGS and Ex-GIS. Recently a four-phase exercise gastrointestinal assessment and intervention protocol has been developed and tested (Supplementary 3- Figure 1), which includes: 1) comprehensive clinical assessment using retrospective exploration; 2) an individual tailored gastrointestinal assessment during exercise using valid and reliable gastrointestinal assessment and analysis methods and techniques; 3) a therapeutic intervention management plan based on outcomes of the assessment and analysis acquired information; and 4) monitoring outcomes of the therapeutic management plan in training and competition, and adjustments made as necessary.[2, 3]

This four-phase approach has been shown to be effective in providing usable data to inform therapeutic intervention management plans, subsequently resulting in a reduction of Ex-GIS in a cohort of endurance and ultra-endurance athletes.[2, 3] The case-series cohort presenting with EIGS and Ex-GIS, underwent a tailored gastrointestinal assessment during exercise informed by their clinical assessment, implemented an individualised therapeutic management plan, and were able to reduce their incidence and severity of Ex-GIS in real-world training and/or competition. Not including two athletes who exhibited substantial intestinal epithelial injury, no substantial perturbations were detected in the circulatory-gastrointestinal pathway of EIGS within the case-series cohort. Notably, there was disturbance to the neuroendocrine-

gastrointestinal pathway of EIGS, with all athletes presenting a hindered orocaecal transit time.[3] The outcomes of these cases suggest the likely reasons for Ex-GIS were due to functional gastrointestinal disturbances; therefore, interventions needed to focus on treating functional issues, rather than epithelial integrity and systemic response issues. Nevertheless, due to the multifaceted and multilayers of EIGS and Ex-GIS, individualised assessment during exercise is fundamental in deciding the most effective prevention and management strategy for each individual, with '*one-size fits all*' and '*trial and error*' approaches consistently failing to be effective. The financial costs of such adherence to a four-phase approach consists of a bare minimum of a practitioner time fee (e.g., ~2-3 h; Sports Dietitians and/or Exercise Physiologist or Sports Medic/Physician with exercise gastroenterology specialisation) that could advise and interpret a basic feeding-challenge during exercise (i.e., tolerance to pre-, during-, and post-competition feeding strategy/plan). Or, a full laboratory exploration that should include physiological and biochemical assessment and analysis, with estimated costs of ~AU$1000. Nevertheless, such costs are minimal considering the acute and longer-term time, equipment and consumable, medical management and clinical support burden and associated costs that EIGS and Ex-GIS make cause in training and competition.

Application in professional sports- Supporting elite athletes within a sports institute:

The majority of athletes in professional, Olympic, and Paralympic sports do not fall into the traditionally known endurance or ultra-endurance category that present the majority of Ex-GIS cases; and as such, are not well represented in current literature.[4] Examples of these sports include all football codes, combat sports, ball sports, swimming and rowing. However, in clinical practice there are many compounding factors within these athletes that induce EIGS and debilitating Ex-GIS, which subsequently translate to direct (i.e., real-time competition) or indirect (i.e., ineffective training load) performance impairment. It is therefore, imperative that

individualised assessment and management of Ex-GIS occurs in these athlete groups. Previous studies have demonstrated the accumulative effect of exercise intensity, duration, environmental factors, and hydration impacting EIGS and Ex-GIS (Main manuscript- Table 1). There may also be additional factors within these sports that, when combined, further exacerbate the extent of Ex-GIS onset and severity (e.g., mechanical strain pathophysiology of EIGS). This may have implications for a wider range of athletes competing in sports of shorter duration including those who train for up to 2.5 h at a time (e.g., rowing and swimming) and other sports (e.g., Australian football, football (soccer), rugby league, field hockey, and/or rugby union) where total time of competition may be ~2 h with consideration for warm-up, competition time, and potential over time/extra time.

When working with athletes within these sports, individualised nutrition support is required to identify, assess, and manage any EIGS and Ex-GIS; such as, following the four-phase exercise gastrointestinal assessment and management protocol discussed above. During an initial sports nutrition consultation, practitioners (e.g., Sport & Exercise Dietitians or Nutritionists, Exercise Physiologist, and/or Sports Medics/Physicians) should include a clinical assessment of the athlete's gastrointestinal function and identify any EIGS and Ex-GIS. Depending on the outcomes of the assessment, referral for medical review may be warranted; as may a referral to a medical practitioner with specific gastrointestinal specialty and/or further assessment at a specialist laboratory. In accordance with such medical management requirements, there is also a paucity of literature investigating other possible situations that promote the onset of EIGS and Ex-GIS as a result of clinical manifestation. For example, the impact of predisposition or established gastrointestinal diseases/disorders,[5] and/or chronic low energy availability (LEA) and subsequent relative energy deficiency in sport (REDs).[6] Additionally, disordered eating (DE) or eating disorders (EDs) have wide ranging implications on the gastrointestinal system

and its function,[6, 7] and individualised management of these conditions including GIS is required. The inter-connection between LEA, REDs, DE and EDs has previously been discussed, and it is highlighted that these conditions can occur together or in isolation, and that assessment of one necessitates the assessment of the other.[8] For example, if an athlete is being assessed for LEA, then a DE/EDs assessment should be conducted simultaneously. The same concept can be applied to gastrointestinal assessment. If an athlete is being assessed initially for a gastrointestinal concern, LEA, REDs, DE or an EDs; the other conditions should also be investigated.

Despite not predominantly being prolonged endurance or ultra-endurance in nature, further exploration in general professional and elite athlete cohorts would be useful to investigate the clinically observed occurrences of the accumulative effects on EIGS and subsequent performance impacting Ex-GIS, as well as the potential accumulative effect of other clinical conditions (e.g., gastrointestinal diseases/disorders, LEA/REDs, and/or DE/EDs. Until then, an individualised approach to assessment and management of professional and elite athletes is warranted.

Application in sports medicine and event medical directorate- Supporting event participants in the field:

GIS (e.g., nausea, vomiting, abdominal pain, and diarrhoea) are often the most commonly reported complaint in competitive and recreational sports; including mass gatherings, long distance running events, international athletic championships, cycling, swimming, and Olympic Games,[4, 9-15] resulting in compromised nutritional intake, performance decrements, and competition withdrawal.[9, 16, 17] GIS during exercise are generally benign; however, occasionally they can be an indication of potentially life-threatening illnesses.[18-22] For example, exercise-associated hyponatraemia, heat stroke, cardiac illness, altitude

illness, and Boerhavaave's syndrome, which need to be treated appropriately by the medical team (Supplementary Figure 2). Outbreaks of infective gastrointestinal illness (e.g., Norovirus outbreaks or other infective pathogens) can cause health risks at sporting events, especially when hygiene levels are reduced, or when performing exercise in contaminated environments (e.g., swimming in lakes or canals or mud splashes to the face in mountain bike races).[11, 13, 18, 19] These may further exacerbate EIGS and Ex-GIS, leading to greater impact on performance decrements and/or clinical outcomes. Thus, preventative measures when planning for sporting events should be considered, such as: planning of location, quality of water supply, preparing and handling of food and beverages, personal hygiene, availability of hand sanitisers at aid stations and toilets, and education on cleanliness behaviours.

In regard to sports medical crew intervention to mitigate or manage EIGS and Ex-GIS, staff can provide supportive care for athletes during and after the event, by educating them on EIGS pathophysiology and exacerbation factors. Antiemetic medications (e.g., cyclizine, metoclopramide, and ondansetron) are often used empirically with some anecdotal evidence; however, in research models, no clear evidence exists that. For example, as described in Supplementary file 2, Ondansetron administration may reduce nausea and vomiting in ultra-endurance athletes during competition.[23] In cases of severe dehydration imposing substantial hypohydration status (Section 7), intravenous fluid substitution may be indicated mid- or post-competition, but current anti-doping regulations need to be followed and application of a therapeutic use exemption (e.g., TUE Physician Guidelines- Intravenous infusion, World Anti-Doping Agency). Loperamide is often used for the symptomatic treatment of diarrhoea and antibiotic treatment for diarrhoea if a specific pathogen is isolated or suspected.[22] Medical event cover can minimise patients' presentation to local health care services, decreasing the burden on local health services and the health infrastructure of the host community.[15] This

approach requires significant planning and cooperation among the race and medical directors and other key players.[24] First aid stations and mobile medical teams may be necessary for properly catering for the needs of the event, including providing first aid, and rapid stabilisation and transport of the athlete within acute life-threatening injuries. Effective communication between the team members and a pre-event medical plan and emergency medical protocols are also recommended.

Supplementary 3- Figure 1. Schematic illustration of four-phase exercise gastrointestinal assessment and intervention protocol with consideration for other clinical manifestations that may instigate or exacerbate EIGS and Ex-GIS.[2, 3, 5, 8] Created in BioRender. Gaskell, S. (2024) https://BioRender.com/z05u680.

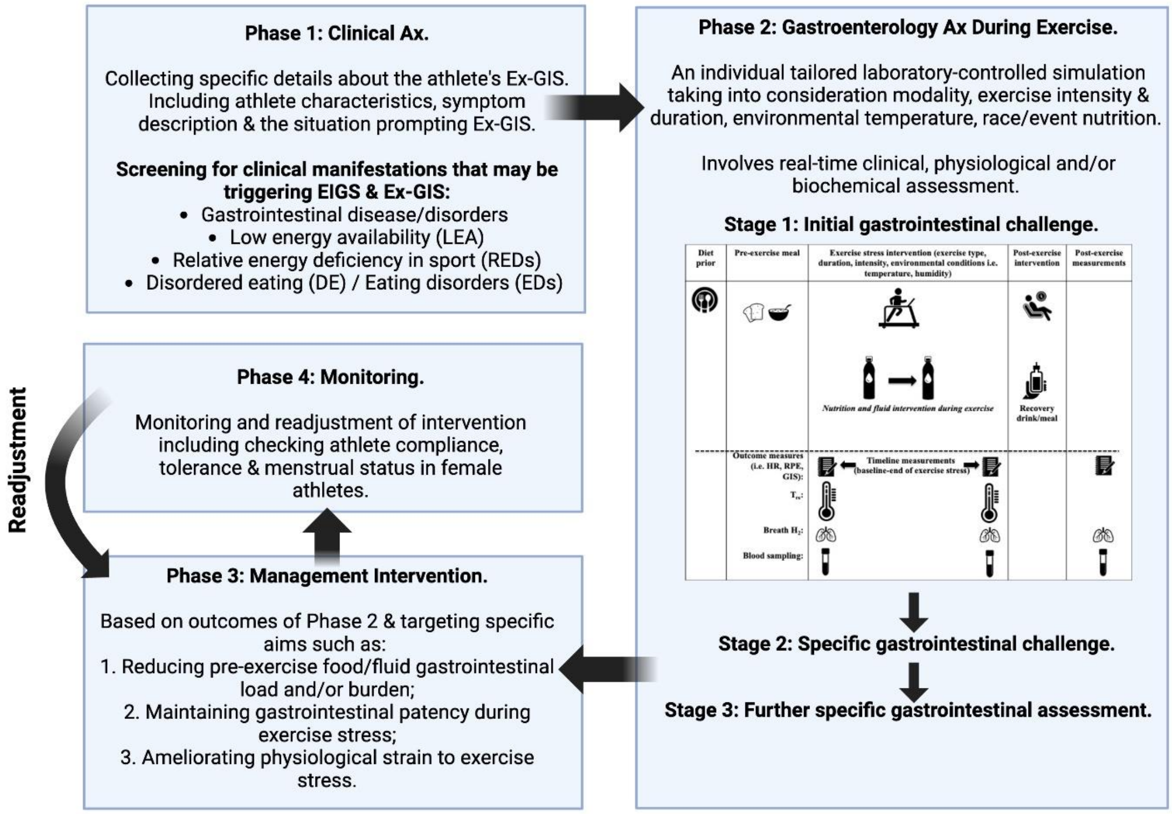

Supplementary 3- Figure 2. Schematic illustration on the evaluation of common causes of nausea and vomiting in endurance sports- field medicine approach.[20, 22]

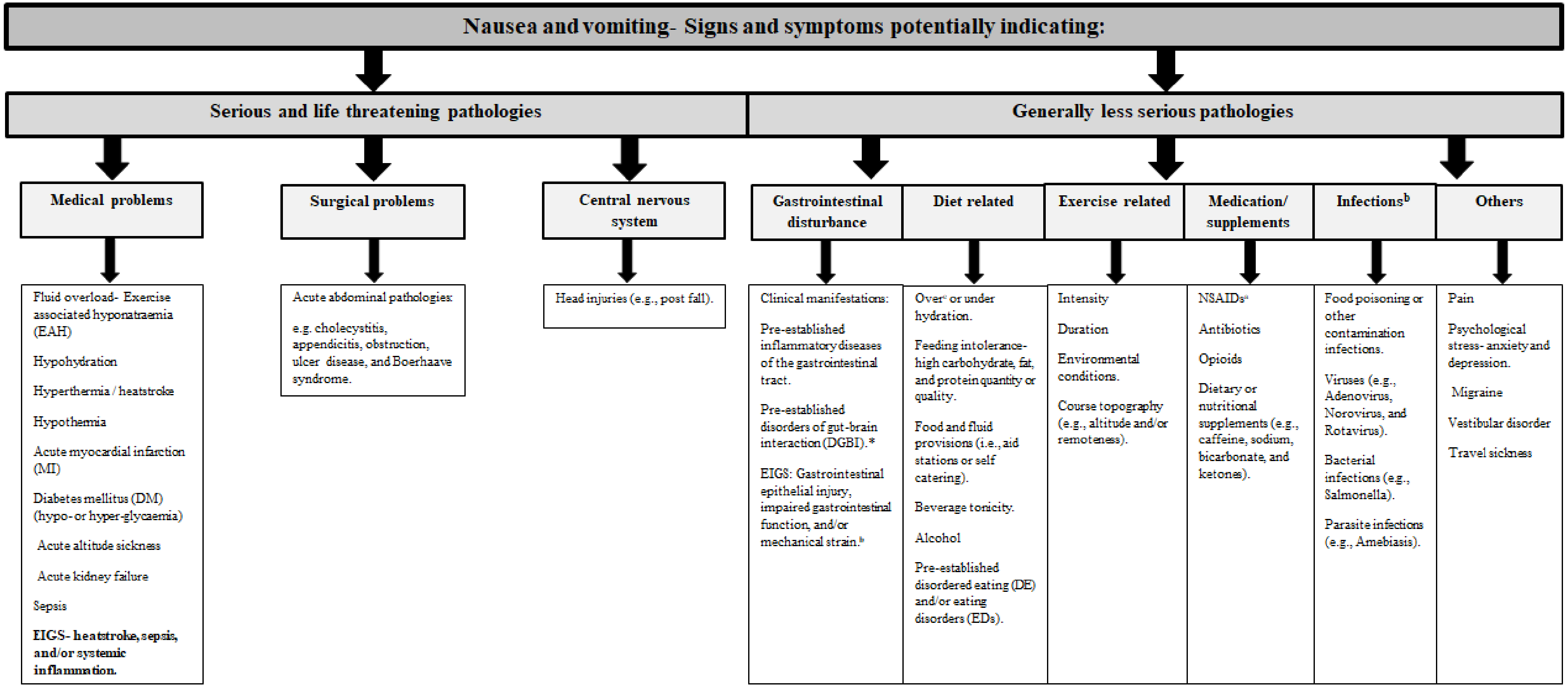

Considerations for in competition assessment

(Potentially) serious/life threatening pathologies:	(Generally) less serious pathologies:
- History and clinical examination.<br>- High index of suspicion for serious pathologies.<br>- Depending on availability of resources and/or remoteness of competition, use of assessment and analysis technologies (e.g., ECG to exclude acute MI risk or onset, blood pressure and heart rate monitoring, point of care blood sampling and analysis (e.g., sodium levels for EAH, glucose for DM, and/or renal function), and/or rectal temperature for hypo- or hyper-thermia risk monitoring).<br>- Treatment strategies depending on symptoms and diagnosis may require urgent evacuation to medical facility.	- Exclusion of serious/life threatening pathologies.<br>- Based on history and clinical examination, symptoms are generally mild and can be treated symptomatically (e.g., reducing exercise intensity, appropriate drinking strategies, cooling or heating strategies).<br>- Dietary intervention/s and/or pharmaceutical intervention/s (e.g., by a license practitioner) may be appropriate. If infective origin is suspected antibiotic treatment may be indicated and consider withdrawal from competition.<br>- Athletes should be monitored for incidence of symptoms and progression of symptoms, and the medical team should remain a high index of suspicion of potentially serious pathologies.

[a] Non-steroidal anti-inflammatory drugs (NSAID) promote acute kidney failure and exercise-associated hyponatraemia (EAH), denoting risk factors for serious pathologies. [b] Bacterial originating infections and EIGS can progress to sepsis. [c] Overhydration is a risk factor for EAH.* Formaly known as functional gastrointestinal disorders. DM: diabetes mellitus, EAH: exercise-associated hyponatraemia, ECG: electrocardiogram, MI: myocardial infarction, NSAID: non-steroidal anti-inflammatory drugs.

References

1. Costa RJS, Young P, Gill SK, Snipe RMJ, Gaskell S, Russo I, et al. Assessment of exercise-associated gastrointestinal perturbations in research and practical settings: Methodological concerns and recommendations for best practice. Int J Sport Nutr Exerc Metab. 2022;32(5):387-418. Epub 20220813. doi: 10.1123/ijsnem.2022-0048. PubMed PMID: 35963615.

2. Gaskell S.K. RC, Costa R.J.S. . Gastrointestinal assessment and management procedures for exercise-associated gastrointestinal symptoms. Aspetar Sports Medicine Journal. 2021;10:36-44.

3. Gaskell SK, Rauch CE, Costa RJS. Gastrointestinal assessment and therapeutic intervention for the management of exercise-associated gastrointestinal symptoms: A case series translational and professional practice approach. Front Physiol. 2021;12:719142. Epub 20210907. doi: 10.3389/fphys.2021.719142. PubMed PMID: 34557109; PubMed Central PMCID: PMCPMC8452991.

4. Engebretsen L, Soligard T, Steffen K, Alonso JM, Aubry M, Budgett R, et al. Sports injuries and illnesses during the London Summer Olympic Games 2012. Br J Sports Med. 2013;47(7):407-14. Epub 20130320. doi: 10.1136/bjsports-2013-092380. PubMed PMID: 23515712.

5. Costa RJS, Snipe RMJ, Kitic CM, Gibson PR. Systematic review: exercise-induced gastrointestinal syndrome-implications for health and intestinal disease. Aliment Pharmacol Ther. 2017;46(3):246-65. Epub 20170607. doi: 10.1111/apt.14157. PubMed PMID: 28589631.

6. Mountjoy M, Ackerman KE, Bailey DM, Burke LM, Constantini N, Hackney AC, et al. 2023 International Olympic Committee's (IOC) consensus statement on Relative Energy

Deficiency in Sport (REDs). Br J Sports Med. 2023;57(17):1073-97. doi: 10.1136/bjsports-2023-106994. PubMed PMID: 37752011.

7. Ackerman KE, Holtzman B, Cooper KM, Flynn EF, Bruinvels G, Tenforde AS, et al. Low energy availability surrogates correlate with health and performance consequences of Relative Energy Deficiency in Sport. Br J Sports Med. 2019;53(10):628-33. Epub 20180602. doi: 10.1136/bjsports-2017-098958. PubMed PMID: 29860237.

8. Wells KR, Jeacocke NA, Appaneal R, Smith HD, Vlahovich N, Burke LM, et al. The Australian Institute of Sport (AIS) and National Eating Disorders Collaboration (NEDC) position statement on disordered eating in high performance sport. Br J Sports Med. 2020;54(21):1247-58. Epub 20200713. doi: 10.1136/bjsports-2019-101813. PubMed PMID: 32661127; PubMed Central PMCID: PMCPMC7588409.

9. Costa RJ, Snipe R, Camões-Costa V, Scheer V, Murray A. The impact of gastrointestinal symptoms and dermatological injuries on nutritional intake and hydration status during ultramarathon events. Sports Med Open. 2016;2:16. Epub 20160105. doi: 10.1186/s40798-015-0041-9. PubMed PMID: 26767151; PubMed Central PMCID: PMCPMC4701764.

10. Edouard P, Depiesse F, Branco P, Alonso JM. Analyses of Helsinki 2012 European Athletics Championships injury and illness surveillance to discuss elite athletes risk factors. Clin J Sport Med. 2014;24(5):409-15. doi: 10.1097/jsm.0000000000000052. PubMed PMID: 24326930.

11. Mexia R, Vold L, Heier BT, Nygård K. Gastrointestinal disease outbreaks in cycling events: are preventive measures effective? Epidemiol Infect. 2013;141(3):517-23. Epub 20120516. doi: 10.1017/s0950268812000817. PubMed PMID: 22591923; PubMed Central PMCID: PMCPMC9151891.

12. Morimura N, Mizobata Y, Sugita M, Takeda S, Kiyozumi T, Shoko T, et al. Medicine at mass gatherings: current progress of preparedness of emergency medical services and disaster medical response during 2020 Tokyo Olympic and Paralympic Games from the perspective of the Academic Consortium (AC2020). Acute Med Surg. 2021;8(1):e626. Epub 20210202. doi: 10.1002/ams2.626. PubMed PMID: 33552526; PubMed Central PMCID: PMCPMC7852170.

13. Parkkali S, Joosten R, Fanoy E, Pijnacker R, J VANB, Brandwagt D, et al. Outbreak of diarrhoea among participants of a triathlon and a duathlon on 12 July 2015 in Utrecht, the Netherlands. Epidemiol Infect. 2017;145(10):2176-84. Epub 20170517. doi: 10.1017/s0950268817001017. PubMed PMID: 28511732; PubMed Central PMCID: PMCPMC9203431.

14. Todkill D, Hughes HE, Elliot AJ, Morbey RA, Edeghere O, Harcourt S, et al. An observational study using english syndromic surveillance data collected during the 2012 London Olympics - What did syndromic surveillance show and what can we learn for future mass-gathering events? Prehosp Disaster Med. 2016;31(6):628-34. Epub 20160919. doi: 10.1017/s1049023x16000923. PubMed PMID: 27641930.

15. Turris SA, Lund A, Bowles RR, Camporese M, Green T. Patient presentations and medical logistics at full and half ironman distance triathlons. Curr Sports Med Rep. 2017;16(3):137-43. doi: 10.1249/jsr.0000000000000367. PubMed PMID: 28498220.

16. Jeukendrup AE, Vet-Joop K, Sturk A, Stegen JH, Senden J, Saris WH, et al. Relationship between gastro-intestinal complaints and endotoxaemia, cytokine release and the acute-phase reaction during and after a long-distance triathlon in highly trained men. Clin Sci (Lond). 2000;98(1):47-55. PubMed PMID: 10600658.

17. Stuempfle KJ, Hoffman MD. Gastrointestinal distress is common during a 161-km ultramarathon. J Sports Sci. 2015;33(17):1814-21. Epub 20150226. doi: 10.1080/02640414.2015.1012104. PubMed PMID: 25716739.

18. Hoffman MD, Weiss RH. The presented evidence to support symptomatic hypovolemic-associated EAH is not convincing. Curr Sports Med Rep. 2017;16(6):464-6. doi: 10.1249/jsr.0000000000000420. PubMed PMID: 29135648.

19. Pasternak A, Ellero J, Maxwell S, Cheung V. Boerhaave's syndrome in an ultra-distance runner. BMJ Case Rep. 2019;12(8). Epub 20190808. doi: 10.1136/bcr-2019-230343. PubMed PMID: 31399415; PubMed Central PMCID: PMCPMC6700565.

20. Scheer V, Murray, A. Ultra-marathon running injuries. In: Doral M, Karlsson, J. , editor. Sports injuries: Prevention, diagnosis, treatment and rehabilitation. Berlin, Germany: Springer-Verlag; 2014.

21. Scorza K, Williams A, Phillips JD, Shaw J. Evaluation of nausea and vomiting. Am Fam Physician. 2007;76(1):76-84. PubMed PMID: 17668843.

22. Hoffman MD, Pasternak A, Rogers IR, Khodaee M, Hill JC, Townes DA, et al. Medical services at ultra-endurance foot races in remote environments: medical issues and consensus guidelines. Sports Med. 2014;44(8):1055-69. doi: 10.1007/s40279-014-0189-3. PubMed PMID: 24748459.

23. Pasternak AV, Fiore D, Islas A, Toti S, Hoffman MD. Treatment with oral ondansetron for ultramarathon-associated nausea: The TOO FUN study. Sports (Basel). 2021;9(3). Epub 20210303. doi: 10.3390/sports9030035. PubMed PMID: 33802399; PubMed Central PMCID: PMCPMC8001581.

24. Martinez JM. Medical coverage of cycling events. Curr Sports Med Rep. 2006;5(3):125-30. doi: 10.1097/01.csmr.0000306301.80201.3d. PubMed PMID: 16640947.
